# Supplementary material for: Design and Synthesis of Menthol and Thymol Derived Ciprofloxacin: Influence of Structural Modifications on the Antibacterial Activity and Anticancer Properties
Source: Int J Mol Sci. 2022 Jun 13;23(12):6600. doi: 10.3390/ijms23126600 (PMC9224473; doi:10.3390/ijms23126600)
Supplement: Supplementary file 1 [file ijms-23-06600-s001.zip › ijms-1744115-supplementary.pdf]

Supporting Information

Table of contents:

1. Preparation of menthol and thymol intermediates used in synthesis of ciprofloxacin conjugates. p. 1-6
2. Numbering of signals in 1H NMR for selected ciprofloxacin conjugates. p. 7-10
3. The NMR spectra of menthol and thymol intermediates. p. 11-34
4. The NMR spectra of ciprofloxacin conjugates in order: **1**, **9**; **2-6**; **10-14**; **7-8**; **15-16**. p. 35-66
5. The HRMS spectra of selected ciprofloxacin conjugates: **1**, **9**; **2**, **8**, **10**, **16**. p. 67-72

1. Preparation of menthol and thymol intermediates used in synthesis of ciprofloxacin conjugates.

1.1. Synthesis of menthol ether derivatives

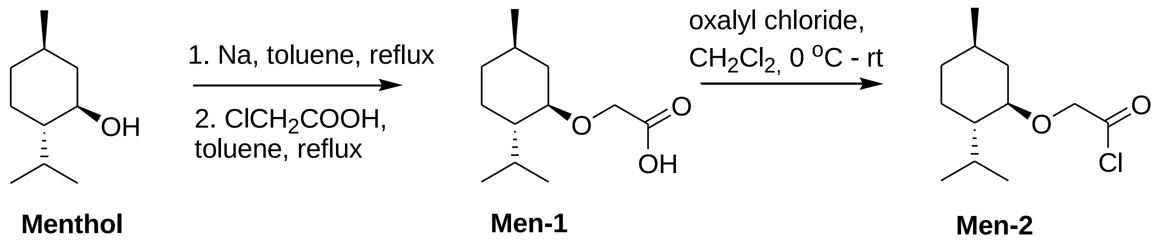

Scheme 1. Synthesis of menthol ether derivative **Men-2**.

1.1.1. (1*R*,2*S*,5*R*)-(2-Isopropyl-5-methyl-cyclohexyloxy)-acetic acid (Men-1)

To 100 ml round bottom flask (-)-menthol (10.0 g, 0.064 mol) and anhydrous toluene (25 mL) were added. To magnetically stirred solution sodium (3.5 g, 0.152 mol) was added and the mixture was refluxed with stirring for 20 h. After cooling to 80 ^{o}C the solution of sodium mentholate was decanted, residual sodium was washed with anhydrous toluene (2 mL) and to hot toluene solution was dropped solution of chloracetic acid (2.36 g, 0.025 mol) in anhydrous toluene (20 mL). The resulting mixture was refluxed with stirring for 28 h. After cooling to 40 ^{o}C to resulting orange paste toluene (25 mL) and water (25 mL) were added and after mixing the phases were separated. The organic phase was additionally extracted with water (2x25 mL). The combined water phases were acidified with 20% HCl_{aq} (20 mL) and extracted with

toluene (3x50 mL). The organic phase was dried with anhydrous MgSO4 and concentrated under reduced pressure. Distillation of residual oil under reduced pressure to gave product as colorless oil; 4.55g (85%), b.p. = 138.5-140.0 ^{o}C/ 3.0-3.5 mbar (114-116 ^{o}C/ 0.35 mmHg)[1].

^{1}H NMR ($CDCl_3$, 300 MHz) δ (ppm): 0.79 (d, *J* = 6.9 Hz, 3H), 0.91 (d, *J* = 7.8 Hz, 3H), 0.93 (d, *J* = 5.4 Hz, 3H), 0.79-1.01 (m, 3H), 1.27-1.39 (m, 2H), 1.62-1.69 (m, 2H), 2.03-2.10 (m, 1H), 2.17-2.228 (m, 1H), 3.31 (dt, *J* = 10.8 Hz, 1H), 4.15 (q, *J* = 16.8 Hz, 2H), 9.93 bs (1H). ^{13}C NMR ($CDCl_3$, 75 MHz) δ (ppm): 16.1, 20.9, 22.2, 23.2, 25.6, 31.5, 34.3, 39.9, 47.9, 65.4, 80.6, 175.0.

1.1.2. (1*R*,2*S*,5*R*)-(2-Isopropyl-5-methyl-cyclohexyloxy)-acetic acid chloride (Men-2)

To magnetically stirred at 0-2 ^{o}C solution of Men-1 (0.30g, 1.40 mmol) in CH_2Cl_2 (4 mL) oxalyl chloride (0.24 mL, 2.80 mmol) was dropped. The resulting solution was stirred at 0-2 ^{o}C for 30 min and then the cooling bath was removed and reaction mixture was stirred and room temperature for 3.5 h. Solvent was evaporated under reduced pressure and the product was used to the next step without further purification.

1.2. Synthesis of thymol ether derivatives

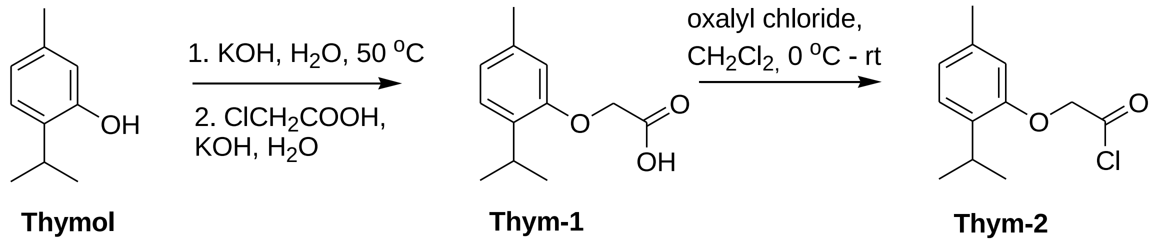

Scheme 2. Synthesis of thymol eter derivative **Thym-2**.

1.2.1. (2-Isopropyl-5-methyl-phenoxy)-acetic acid (Thym-1)

To 50 ml round bottom flask thymol (2.0g, 13.3 mmol), water (15 mL) and solid KOH (1 g, 0.018 mol) were added. This mixture was warmed on a water-bath at 50 ^{o}C until complete solution took place. In the second flask chloracetic acid (1.36 g, 14.3 mmol) was dissolved in water (10 mL) and 2 drops of phenolphthalein was added. Next, to this solution 10% KOH_{aq} solution was added until a permanent pink color was obtained. The two previously obtained

solution were then mixed and refluxed for 20 min. The hot mixture was filtered, the filtrate was cooled and acidified with concentrated hydrochloric acid (3 mL). After few seconds beige solid was precipitated. The solvent was decanted, solid was washed with water (3x10 mL) and solid was dried by toluene addition (100 mL) and evaporation this solvent under reduced pressure. The residual solid was washed with hot hexane (15 mL) and dried under reduced pressure. The product was obtained as white solid, 0.60g (22%), mp = 150.1-151.1 ^{o}C (142-144 ^{o}C)[2].

^{1}H NMR ($CDCl_3$, 300 MHz) δ (ppm): 1.22 (d, *J* = 6.9 Hz, 6H), 2.31 (s, 3H), 3.27-3.41 (m, 1H), 4.69 (s, 2H), 6.59 (bs, 1H), 6.79-6.83 (m, 1H), 7.13 (d, *J* = 7.8 Hz, 1H), 10.43 (bs, 1H).

^{13}C NMR ($CDCl_3$, 75 MHz) δ (ppm): 21.3, 22.8 (2xC), 26.6, 65.2, 112.4, 122.7, 126.4, 134.6, 136.5, 154.6, 174.8.

1.2.2. (2-Isopropyl-5-methyl-phenoxy)-acetic acid chloride (Thym-2)

To magnetically stirred at 0-2 ^{o}C solution of Thym-1 (0.20g, 0.96 mmol) in CH_2Cl_2 (4 mL) oxalyl chloride (0.16 mL, 1.92 mmol) was dropped. The resulting solution was stirred at 0-2 ^{o}C for 30 min and then the cooling bath was removed and reaction mixture was stirred and room temperature for 6 h. Solvent was evaporated under reduced pressure and the product was used to the next step without further purification.

1.3. Synthesis of menthol and thymol ester derivatives

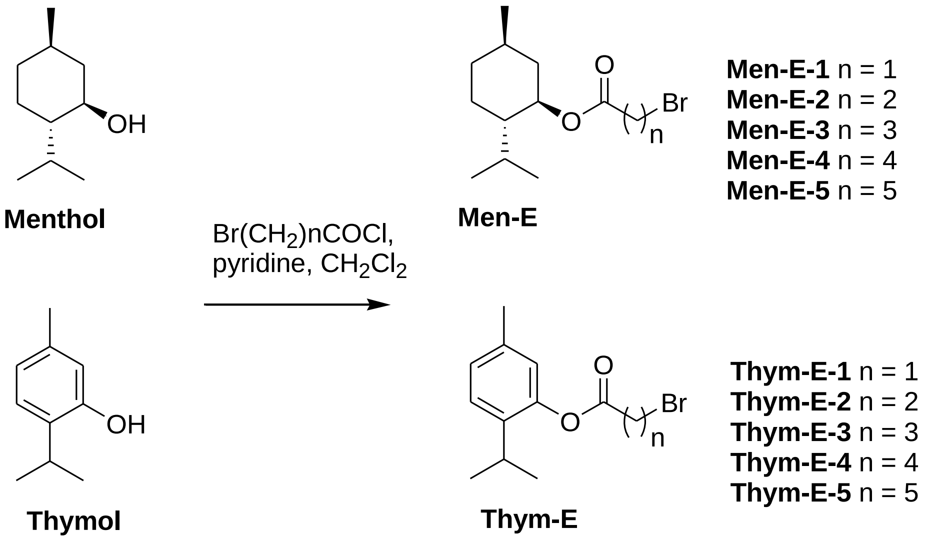

Scheme 3. Synthesis of menthol and thymol ester derivatives.

General procedure for the synthesis of menthol and thymol esters

To a magnetically stirred at 0-2 oC solution of thymol (0.90 g, 6.00 mmol) in CH2Cl2 (40 mL) pyridine (0.73 mL, 9.00 mmol) was added and next solution of appropriate bromoacyl chloride (9.00 mmol) in CH_2Cl_2 (20 mL) was added dropwise. After 45 min the cooling bath was removed and the solution was stirred at room temperature for 4 h. The reaction mixture was washed with water (50 mL), 1% HCl_{aq} solution (50 mL), water (50 mL) and dried over anhydrous Na_2SO_4. After evaporation of the solvent the product was isolated by column chromatography on silica gel (230-400 mesh) using hexane as mobile phase.

1.3.1. Menthyl bromoacetate (Men-E-1)

Colorless oil, 70%.[3]

^{1}H NMR ($CDCl_3$, 300 MHz) δ (ppm): 0.77 (d, *J* = 6.9 Hz, 3H), 0.85-0.96 (m, 1H), 0.90 (d, *J* = 3.6 Hz, 3H), 0.92 (d, *J* = 3.6 Hz, 3H), 1.00-1.12 (m, 2H), 1.38-1.50 (m, 2H), 1.65-1.73 (m, 2H), 1.85-1.92 (m, 1H), 1.98-2.05 (m, 1H), 3.80 (d, *J* = 1.5 Hz, 2H), 4.73 (dt, *J* = 4.5 Hz, 10.8 Hz, 1H). ^{13}C NMR ($CDCl_3$, 75 MHz) δ (ppm): 16.2, 20.7, 22.0, 23.4, 26.2, 26.3, 31.4, 34.1, 40.5, 47.0, 76.4, 166.9.

1.3.2. Menthyl 3-bromopropionate (Men-E-2)

Colorless oil, 80%.

^{1}H NMR ($CDCl_3$, 500 MHz) δ (ppm): 0.76 (d, *J* = 7.0 Hz, 3H), 0.83-0.86 (m, 1H), 0.90 (d, *J* = 6.5 Hz, 3H), 0.92 (d, *J* = 6.0 Hz, 3H), 0.96-1.10 (m, 2H), 1.36-1.42 (m, 1H), 1.45-1.55 (m, 1H), 1.66-1.70 (m, 2H), 1.86-1.92 (m, 1H), 1.99-2.03 (m, 1H), 2.90 (t, *J* = 7.0 Hz, 2H), 3.80 (dt, *J* = 1.5 Hz, 7.0 Hz, 2H), 4.74 (dt, *J* = 4.5 Hz, 10.5 Hz, 1H). ^{13}C NMR ($CDCl_3$, 125 MHz) δ (ppm): 16.3, 20.8, 22.0, 23.4, 26.3 (2xC), 31.4, 34.2, 38.1, 40.9, 47.0, 75.1, 166.9.

1.3.3. Menthyl 4-bromobutyrate (Men-E-3)

Colorless oil, 79%.[4]

^{1}H NMR ($CDCl_3$, 500 MHz) δ (ppm): 0.76 (d, *J* = 7.0 Hz, 3H), 0.83-0.89 (m, 1H), 0.90 (d, *J* = 4.5 Hz, 3H), 0.91 (d, *J* = 4.0 Hz, 3H), 0.94-1.10 (m, 2H), 1.34-1.40 (m, 1H), 1.44-1.51 (m, 1H), 1.65-1.70 (m, 2H), 1.82-1.88 (m, 1H), 1.96-2.00 (m, 1H), 2.14-2.20 (m, 2H), 2.48 (dt, *J* = 1.5 Hz, 7.5 Hz, 2H), 3.46 (dt, *J* = 1.0 Hz, 6.5 Hz, 2H), 4.74 (dt, *J* = 4.5 Hz, 10.5 Hz, 1H). ^{13}C NMR ($CDCl_3$, 125 MHz) δ (ppm): 16.3, 20.8, 22.0, 23.4, 26.3, 27.9, 31.4, 32.7, 32.9, 34.2, 40.9, 47.0, 74.4, 172.1.

1.3.4. Menthyl 5-bromopentanoate (Men-E-4)

Colorless oil, 87%.

^{1}H NMR ($CDCl_3$, 500 MHz) δ (ppm): 0.76 (d, *J* = 7.0 Hz, 3H), 0.83-0.88 (m, 1H), 0.89 (d, *J* = 4.0 Hz, 3H), 0.91 (d, *J* = 3.5 Hz, 3H), 0.93-1.10 (m, 2H), 1.34-1.40 (m, 1H), 1.44-1.54 (m, 1H), 1.65-1.70 (m, 2H), 1.75-1.81 (m, 2H), 1.82-1.87 (m, 1H), 1.87-1.93 (m, 2H), 1.96-2.00 (m, 1H), 2.32 (t, *J* = 7.0 Hz, 2H), 3.42 (t, *J* = 6.5 Hz, 2H), 4.68 (dt, *J* = 4.5 Hz, 11.0 Hz, 1H). ^{13}C NMR ($CDCl_3$, 125 MHz) δ (ppm): 16.3, 20.8, 22.0, 23.4, 23.7, 26.3, 31.4, 32.0, 33.1, 33.7, 34.3, 41.0, 47.0, 74.2, 172.7.

1.3.5. Menthyl 6-bromohexanoate (Men-E-5)

Colorless oil, 94%.

^{1}H NMR ($CDCl_3$, 500 MHz) δ (ppm): 0.76 (d, *J* = 7.0 Hz, 3H), 0.82-0.88 (m, 1H), 0.89 (d, *J* = 4.0 Hz, 3H), 0.91 (d, *J* = 3.5 Hz, 3H), 0.92-1.00 (m, 1H), 1.01-1.10 (m, 1H), 1.34-1.40 (m, 1H), 1.44-1.54 (m, 3H), 1.63-1.70 (m, 4H), 1.83-1.91 (m, 3H), 1.96-2.00 (m, 1H), 2.30 (t, *J* = 7.0 Hz, 2H), 3.41 (t, *J* = 6.5 Hz, 2H), 4.68 (dt, *J* = 4.5 Hz, 11.0 Hz, 1H). ^{13}C NMR ($CDCl_3$, 125 MHz) δ (ppm): 16.3, 20.8, 22.0, 23.4, 24.3, 26.3, 27.7, 31.4, 32.4, 33.5, 34.3, 34.5, 41.0, 47.0, 74.1, 173.0.

1.3.6. Thymol bromoacetate (Thym-E-1)

Colorless oil, 82%.

^{1}H NMR ($CDCl_3$, 300 MHz) δ (ppm): 1.20 (d, *J* = 7.5 Hz, 6H), 2.32 (s, 3H), 2.96-3.01 (m, 1H), 4.05 (s, 2H), 6.83 (bs, 1H), 7.03-7.07 (m, 1H), 7.21 (d, *J* = 7.8 Hz, 1H). ^{13}C NMR ($CDCl_3$, 75 MHz) δ (ppm): 20.8, 23.0 (2xC), 25.3, 26.9, 122.1, 126.6, 127.7, 136.8, 137.0, 147.5, 166.1.

1.3.7. Thymol 3-bromopropionate (Thym-E-2)

Colorless oil, 62%.

^{1}H NMR ($CDCl_3$, 300 MHz) δ (ppm): 1.19 (d, *J* = 7.2 Hz, 6H), 2.31 (s, 3H), 2.93-3.06 (m, 1H), 3.19 (t, *J* = 6.6 Hz, 2H), 3.70 (t, *J* = 6.6 Hz, 2H), 6.82 (bs, 1H), 7.02-7.05 (m, 1H), 7.21 (d, *J* = 7.8 Hz, 1H). ^{13}C NMR ($CDCl_3$, 75 MHz) δ (ppm): 20.8, 23.1 (2xC), 25.7, 27.0, 37.8, 122.5, 126.5, 127.4, 136.6, 137.0, 147.6, 169.3.

1.3.8. Thymol 4-bromobutyrate (Thym-E-3)

Colorless oil, 88%.

^{1}H NMR ($CDCl_3$, 500 MHz) δ (ppm): 1.19 (d, *J* = 7.0 Hz, 6H), 2.27-2.33 (m, 2H), 2.31 (s, 3H), 2.80 (t, *J* = 7.0 Hz, 2H), 2.92-2.98 (m, 1H), 3.55 (t, *J* = 6.5 Hz, 2H), 6.80 (bs, 1H), 7.02 (d, *J* = 8.0 Hz, 1H), 7.19 (d, *J* = 8.0 Hz, 1H). ^{13}C NMR ($CDCl_3$, 125 MHz) δ (ppm): 20.8, 23.0 (2xC), 27.2, 27.6, 32.4, 32.5, 122.6, 126.5, 127.2, 136.6, 136.9, 147.7, 171.3.

1.3.9. Thymol 5-bromopentanoate (Thym-E-4)

Colorless oil, 88%.

^{1}H NMR ($CDCl_3$, 500 MHz) δ (ppm): 1.19 (d, *J* = 7.0 Hz, 6H), 1.92-2.02 (m, 4H), 2.31 (s, 3H), 2.62 (t, *J* = 7.0 Hz, 2H), 2.92-2.98 (m, 1H), 3.46 (t, *J* = 6.5 Hz, 2H), 6.80 (bs, 1H), 7.02 (d, *J* = 8.0 Hz, 1H), 7.19 (d, *J* = 7.5 Hz, 1H). ^{13}C NMR ($CDCl_3$, 125 MHz) δ (ppm): 20.8, 23.0 (2xC), 23.6, 27.1, 32.0, 32.9, 33.4, 122.7, 126.4, 127.2, 136.6, 136.9, 147.8, 171.8.

1.3.10. Thymol 6-bromohexanoate (Thym-E-5)

Colorless oil, 82%.

^{1}H NMR ($CDCl_3$, 500 MHz) δ (ppm): 1.19 (d, *J* = 6.5 Hz, 6H), 1.55-1.61 (m, 2H), 1.77-1.84 (m, 2H), 1.91-1.96 (m, 2H), 2.31 (s, 3H), 2.60 (t, *J* = 7.5 Hz, 2H), 2.93-2.98 (m, 1H), 3.44 (t, *J* = 7.0 Hz, 2H), 6.79 (bs, 1H), 7.02 (d, *J* = 8.0 Hz, 1H), 7.19 (d, *J* = 7.5 Hz, 1H). ^{13}C NMR ($CDCl_3$, 125 MHz) δ (ppm): 20.8, 23.0 (2xC), 24.2, 27.1, 27.7, 32.4, 33.4, 34.1, 122.7, 126.4, 127.1, 136.6, 137.0, 147.8, 172.1.

1. Galpin, D.R., Huitric, A.C., J. Org. Chem., 1968, 33, 2, 921-923.
2. Pawełczyk, A., Sowa-Kasprzyk, A., Olender, D., Zaprutko, L., Molecules, 2018, 23, 2360.
3. Singh, R., Gosh, S. K, Tetrahedron: Asymmetry, 2014, 25, 57-62.
4. Ha, T. H., Suh, K-H., Lee G. S., Bull. Korean Chem. Soc., 2013, 34, 549-552.

2. Numbering of signals in ^{1}H NMR for selected ciprofloxacin conjugates.

*1-cyclopropyl-6-fluoro-7-{4-[2-((1R,2S,5R)-2-isopropyl-5-methylcyclohexyloxy)-acetyl]piperazin-1-yl}-4-oxo-1,4-dihydroquinoline-3-carboxylic acid (**1**)*

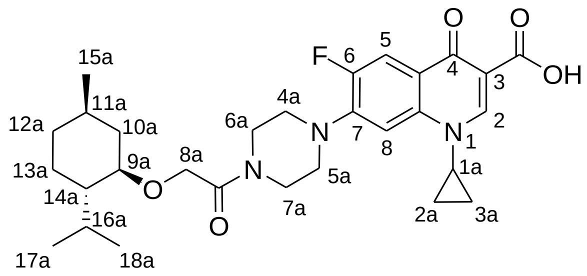

White solid. Yield 92%. Mp = 185.7-189.6 ^{o}C.

^{1}H NMR ($CDCl_3$, 300 MHz) δ (ppm): 0.72 (d, *J* = 6.6 Hz, 3H-15a), 0.89-0.93 (m, 3H-17a, 3H-18a, 1H-10a, 1H-12a, 1H-13a), 1.15-1.34 (m, 2H-2a, 2H-3a, 1H-11a, 1H-14a), 1.53-1.61 (m, 1H-12a, 1H-13a), 2.06-2.17 (m, 1H-10a, 1H-16a), 3.01-3.18 (m, 1H-9a), 3.27-3.30 (m, 4H-6a, 7a), 3.51 (bs, 1H-1a), 3.71-3.81 (m, 4H-4a, 5a), 4.05-4.24 (m, 2H-8a), 7.29 (d, *J* = 7.2 Hz, 1H-8), 7.85 (d, *J* = 12.9 Hz, 1H-5), 8.60 (s, 1H-2), 14.81 (s, 1H-OH).

*1-cyclopropyl-6-fluoro-7-{4-[2-(2-isopropyl-5-methylphenoxy)acetyl]piperazin-1-yl}-4-oxo-1,4-dihydroquinoline-3-carboxylic acid (**9**)*

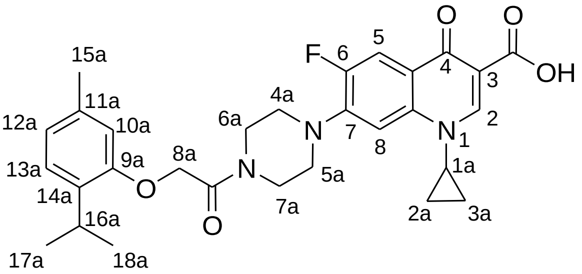

White solid. Yield 81%. Mp = 232.8-234.0 ^{o}C.

^{1}H NMR ($CDCl_3$\CD_3OD, 9:1 mixture, 300 MHz) δ (ppm): 1.18-1.20 (m, 2H-2a), 1.21 (d, *J* = 7.0 Hz, 6H-17a, 18a), 1.39-143 (m, 2H-3a), 2.33 (s, 3H-15a), 3.37-3.93 (m, 4H-6a, 7a, 1H-16a), 3.54-3.61 (m, 1H-1a), 3.90 (t, *J* = 5.1 Hz, 4H-4a, 5a), 4.76 (s, 2H-8a), 6.72 (bs, 1H-10a), 6.82 (d, *J* = 7.8 Hz, 1H-12a), 7.14 (d, *J* = 7.5 Hz, 1H-13a), 7.39 (d, *J* = 6.9 Hz, 1H-8), 8.04 (d, *J* = 12.9 Hz, 1H-5), 8.79 (s, 1H-2).

*1-cyclopropyl-6-fluoro-7-{4-[2-((1R,2S,5R)-2-isopropyl-5-methylcyclohexyloxy)-2-oxoethyl]piperazin-1-yl}-4-oxo-1,4-dihydroquinoline-3-carboxylic acid (**2**)*

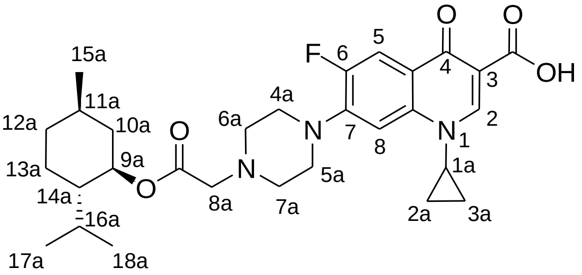

White solid. Yield 63%. Mp = 214.5-215.4 ^{o}C.

^{1}H NMR ($CDCl_3$, 300 MHz) δ (ppm): 0.78 (d, *J* = 6.9 Hz, 3H-15a), 0.84-0.96 (m, 1H-12a), 0.90 (d, *J* = 2.4 Hz, 3H-17a), 0.92 (d, *J* = 2.1 Hz, 3H-18a), 0.96-1.14 (m, 1H-10a, 1H-13a), 1.18-1.23 (m, 2H-2a), 1.37-1.55 (m, 2H-3a, 1H-11a, 1H-14a), 1.69-1.72 (m, 1H-12a, 1H-13a), 1.82-1.89 (m, 1H-10a), 1.99-2.04 (m, 1H-16a), 2.84 (bs, 4H-6a,7a), 3.30(d, *J* = 2.7 Hz, 2H-8a), 3.41 (t, *J* = 4.5 Hz, 4H-4a, 5a), 3.51-3.61 (m, 1H-1a), 4.77(dt, *J* = 4.5 Hz, 10.8 Hz, 1H-9a), 7.37 (d, *J* = 7.2 Hz, 1H-8), 7.95 (d, *J* = 12.9 Hz, 1H-5), 8.72 (s, 1H-2), 15.00 (s, 1H-OH).

*1-cyclopropyl-6-fluoro-7-{4-[2-(2-isopropyl-5-methylphenoxy)acetyl]piperazin-1-yl}-4-oxo-1,4-dihydroquinoline-3-carboxylic acid (**10**)*

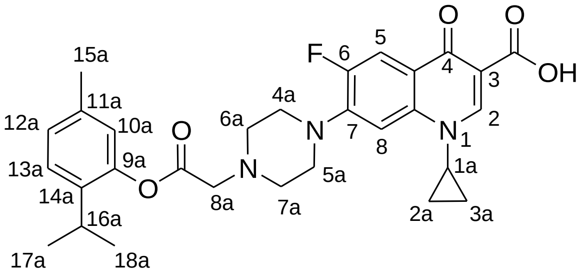

Pale beige solid. Yield 65%. Mp = 240.2-241.8 ^{o}C.

^{1}H NMR ($CDCl_3$\CD_3OD, 9:1 mixture, 500 MHz) δ (ppm): 1.18-1.20 (m, 2H-2a), 1.21 (d, *J* = 7.0 Hz, 6H-17a, 18a), 1.39-143 (m, 2H-3a), 2.33 (s, 3H-15a), 2.95 (t, *J* = 4.0 Hz, 4H-6a, 7a), 3.38-3.40 (m, 1H-16a), 3.45 (t, *J* = 4.5 Hz, 4H-4a, 5a), 3.56-3.60 (m, 1H-1a), 3.63 (s, 2H-8a), 6.83 (bs, 1H-10a), 7.04-7.06 (m, 1H-12a), 7.22 (d, *J* = 8.0 Hz, 1H-13a), 7.40 (d, *J* = 7.0 Hz, 1H-8), 7.98 (d, *J* = 13.0 Hz, 1H-5), 8.76 (s, 1H-2).

*5-((1R,2S,5R)-2-isopropyl-5-methylcyclohexyloxy)-5-oxopentyl-1-cyclopropyl-6-fluoro-7-{4-[5-((1R,2S,5R)-2-isopropyl-5-methylcyclohexyloxy)-5-oxopentyl]piperazin-1-yl}-4-oxo-1,4-dihydroquinoline-3-carboxylate (**7**)*

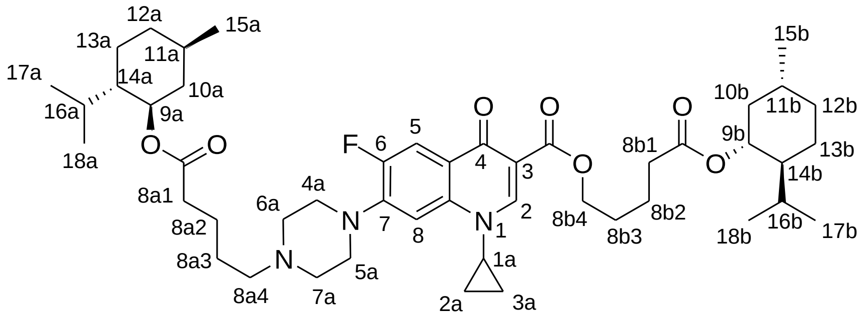

Solidifying oil. Yield 7%.

^{1}H NMR ($CDCl_3$, 300 MHz) δ (ppm): 0.75 (d, *J* = 4.8 Hz, 3H-15b), 0.77 (d, *J* = 4.8 Hz, 3H-15a), 0.84-0.92 (m, 12H-17a, 18a, 17b, 18b, 1H-12a, 1H-12b), 0.95-1.16 (m, 2H-2a, 1H-13a, 1H-13b, 1H-10a, 1H-10b,), 1.28-1.51 (m, 2H-3a, 1H-14a, 1H-14b, 1H-11a, 1H-11b), 1.64-1.72 (m, 4H-8a3, 8b3, 1H-12a, 1H-12b, 1H-13a, 1H-13b), 1.78-1.90 (m, 4H-8a2, 8b2, 1H-10a, 1H-10b), 1.94-2.02 (m, 2H-16a, 16b), 2.31-2.39 (m, 4H-8a1, 8b1), 2.45 (t, *J* = 4.2 Hz, 2H-8a4), 2.65 (t, *J* = 4.8 Hz, 4H-6a, 7a), 3.29 (t, *J* = 4.8 Hz, 4H-4a, 5a), 3.38-3.46 (m, 1H-1a), 4.32 (t, *J* = 6.0 Hz, 2H-8b4), 4.63-4.74 (m, 2H-9a, 9b), 7.27 (d, *J* = 7.2 Hz, 1H-8), 8.04 (d, *J* = 13.5 Hz, 1H-5), 8.52 (s, 1H-2).

*2-(2-isopropyl-5-methylphenoxy)-2-oxoethyl-1-cyclopropyl-6-fluoro-7-{4-[2-(2-isopropyl-5-methylphenoxy)-2-oxoethyl]piperazin-1-yl}-4-oxo-1,4-dihydroquinoline-3-carboxylate (**15**)*

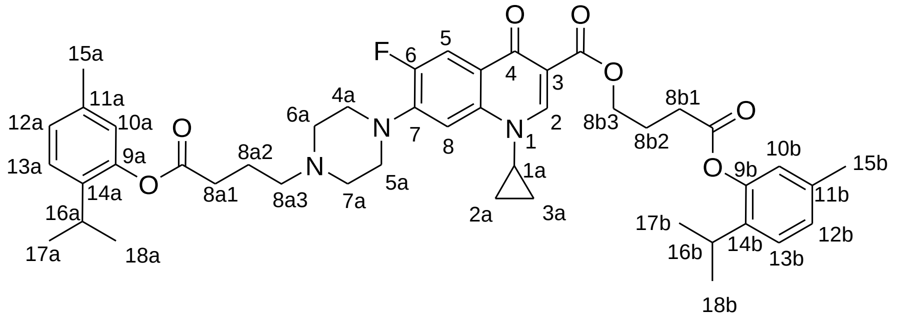

White solid. Yield 21%. Mp = 105.5-107.2 ^{o}C.

^{1}H NMR ($CDCl_3$, 500 MHz) δ (ppm): 1.06-1.09 (m, 2H-2a), 1.16 (d, *J* = 7.0 Hz, 6H-17a, 18a), 1.20 (d, *J* = 7.0 Hz, 6H-17b, 18b), 1.24-1.28 (m, 2H-3a), 1.98-2.04 (m, 2H-8a2), 2.23 (s, 3H-15a), 2.24-2.28 (m, 2H-8b2), 2.31 (s, 3H-15b), 2.56 (t, *J* = 7.0 Hz, 2H-8a1), 2.68 (t, *J* =

7.0 Hz, 2H-8b1), 2.71 (t, *J* = 4.0 Hz, 4H-6a, 7a), 2.83 (t, *J* = 7.5 Hz, 2H-8a1), 2.93-3.01 (m, 2H-16a, 16b), 3.30 (t, *J* = 4.5 Hz, 4H-4a, 5a), 3.33-3.38 (m, 1H-1a), 4.43 (t, *J* = 6.5 Hz, 2H-8b3), 6.78 (s, 1H-10b), 6.82 (s, 1H-10a), 6.99 (d, *J* = 8.0 Hz, 1H-12b), 7.02 (d, *J* = 7.5 Hz, 1H-12a), 7.17 (d, *J* = 7.5 Hz, 1H-13b), 7.20 (d, *J* = 8.0 Hz, 1H-13a), 7.26 (d, *J* = 7.5 Hz, 1H-8), 8.02 (d, *J* = 13.5 Hz, 1H-5), 8.48 (s, 1H-2).

3. The NMR spectra of menhtol and thymol intermediates.

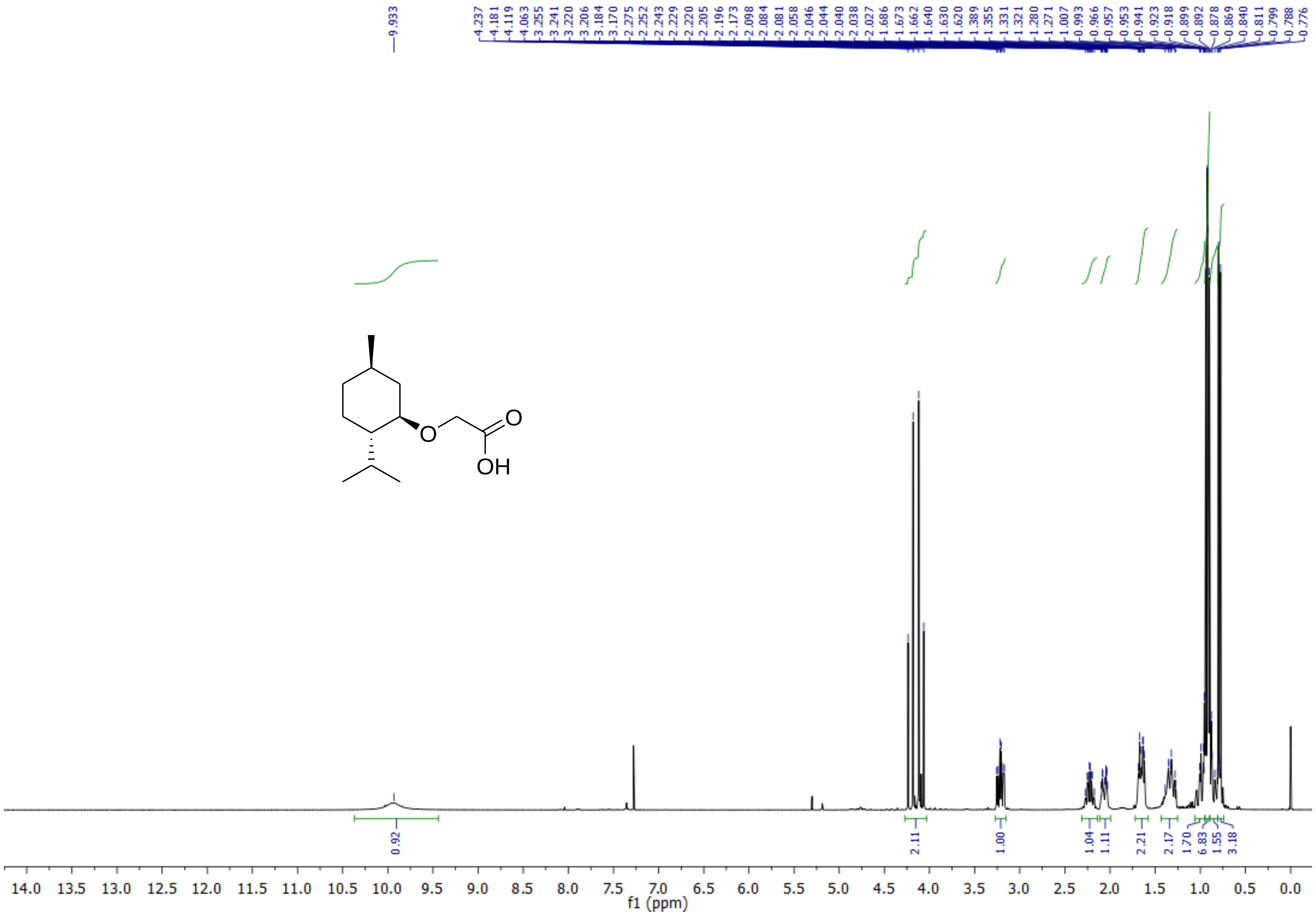

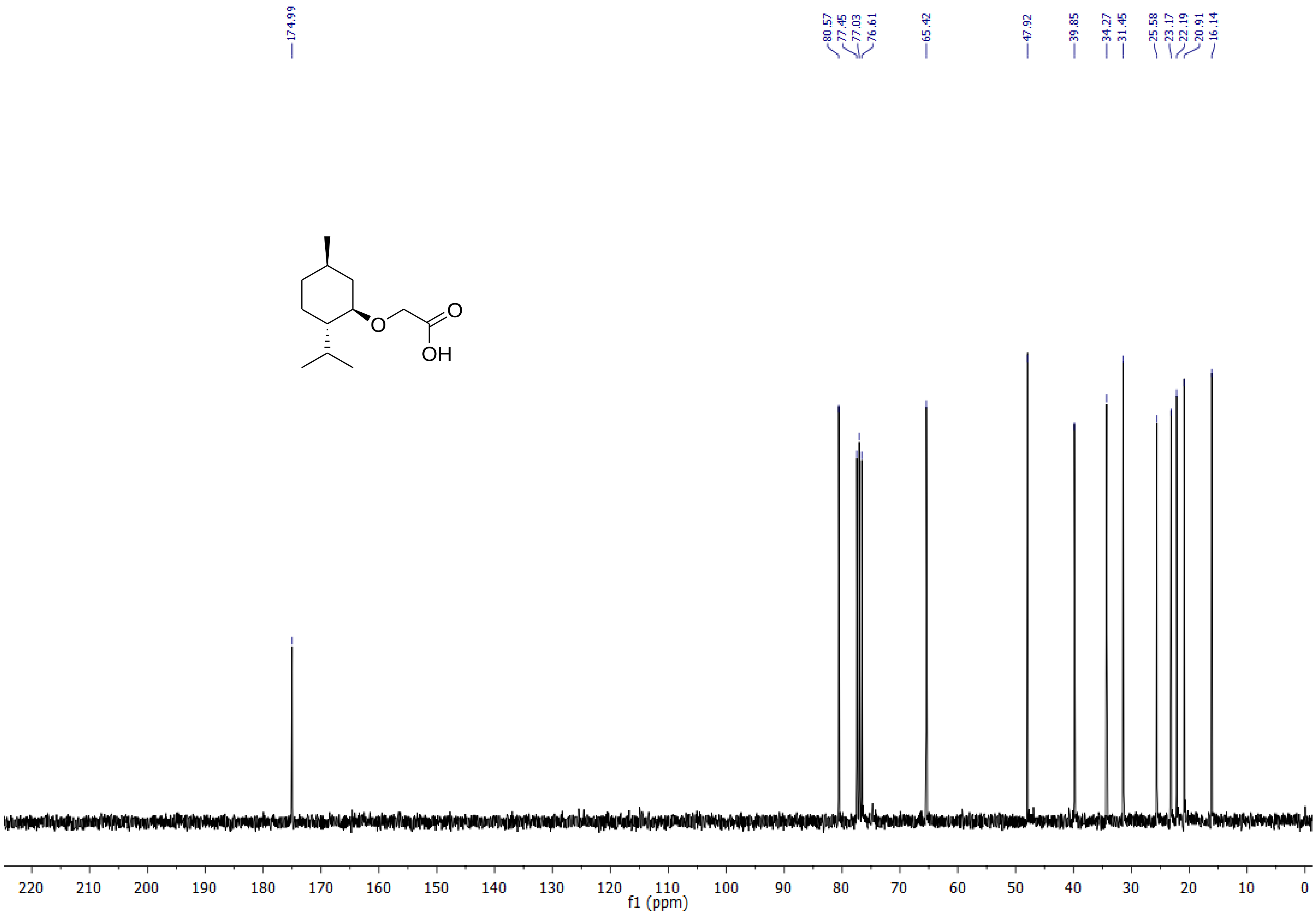
174.99
80.57
77.45
77.03
76.61
65.42
47.92
39.85
34.27
31.45
25.58
23.17
22.19
20.91
16.14
O
O
OH
220
210
200
190
180
170
160
150
140
130
120
110
100
90
80
70
60
50
40
30
20
10
0
f1 (ppm)

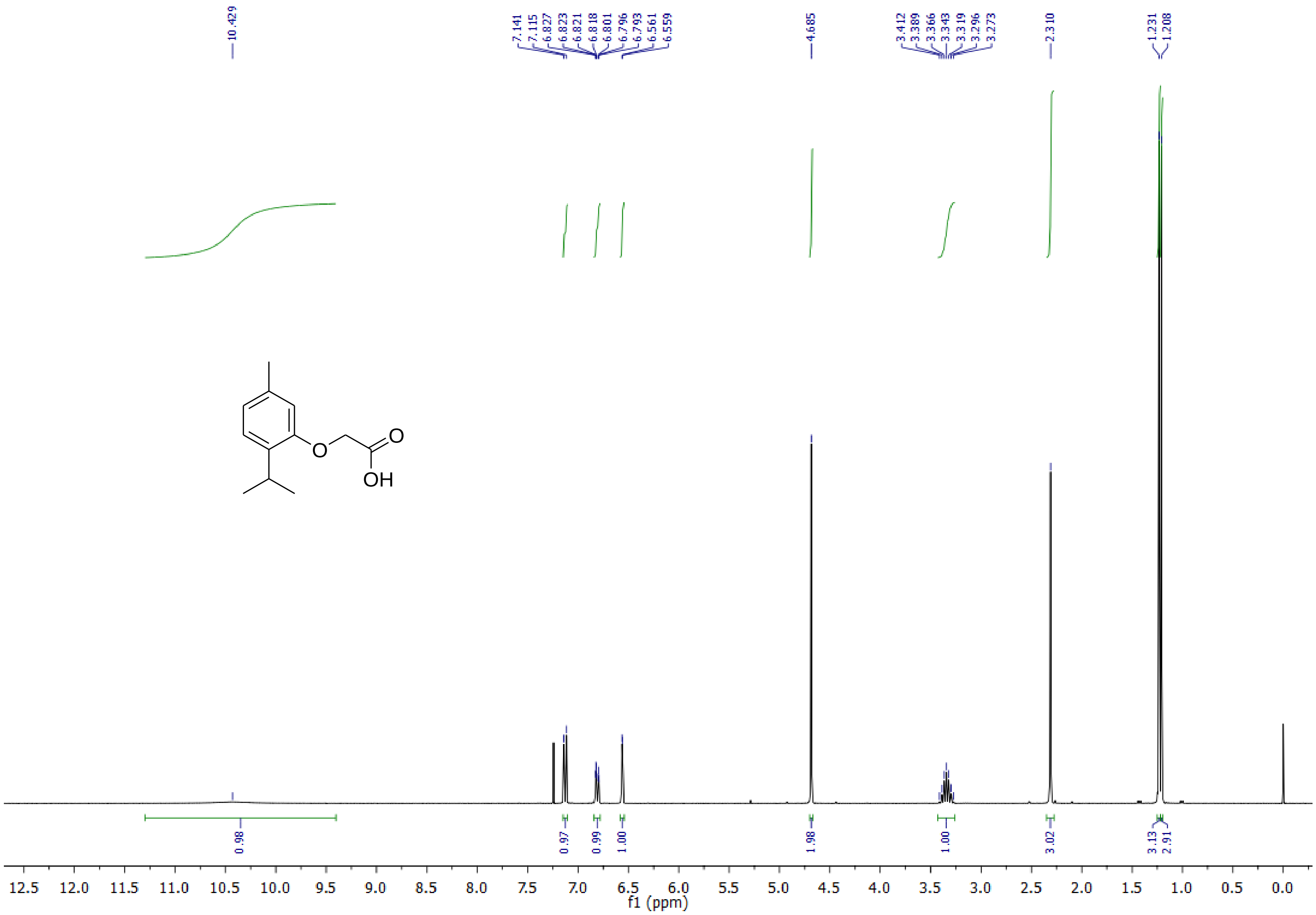
10.429
7.141
7.115
6.827
6.823
6.821
6.818
6.801
6.796
6.793
6.561
6.559
4.685
3.412
3.389
3.366
3.343
3.319
3.296
3.273
2.310
1.231
1.208
O
OH
O
0.98
0.97
0.99
1.00
1.98
1.00
3.02
3.13
2.91
12.5 12.0 11.5 11.0 10.5 10.0 9.5 9.0 8.5 8.0 7.5 7.0 6.5 6.0 5.5 5.0 4.5 4.0 3.5 3.0 2.5 2.0 1.5 1.0 0.5 0.0
f1 (ppm)

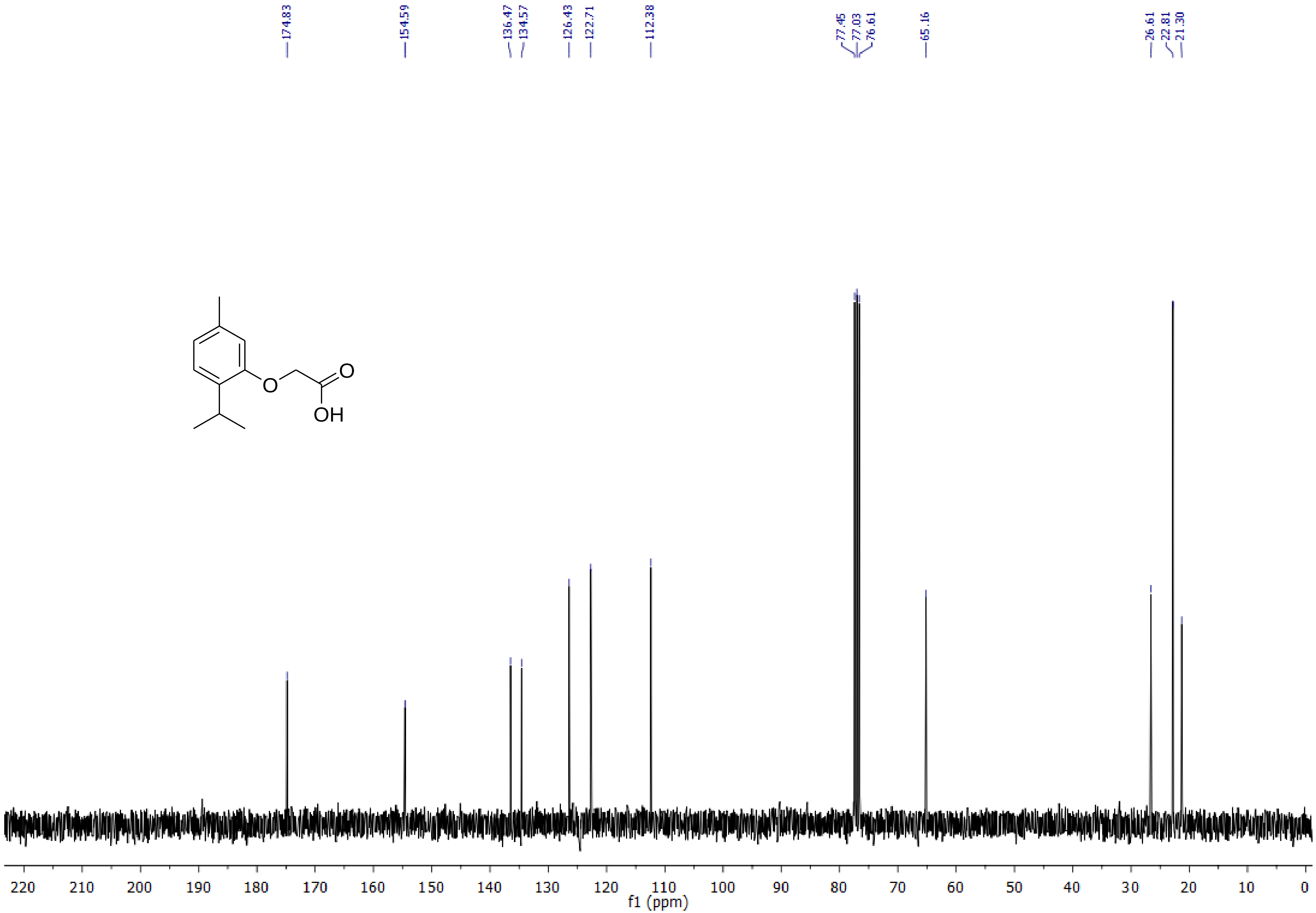
174.83
154.59
136.47
134.57
126.43
122.71
112.38
77.45
77.03
76.61
65.16
26.61
22.81
21.30
O
OH
220 210 200 190 180 170 160 150 140 130 120 110 100 90 80 70 60 50 40 30 20 10 0
f1 (ppm)

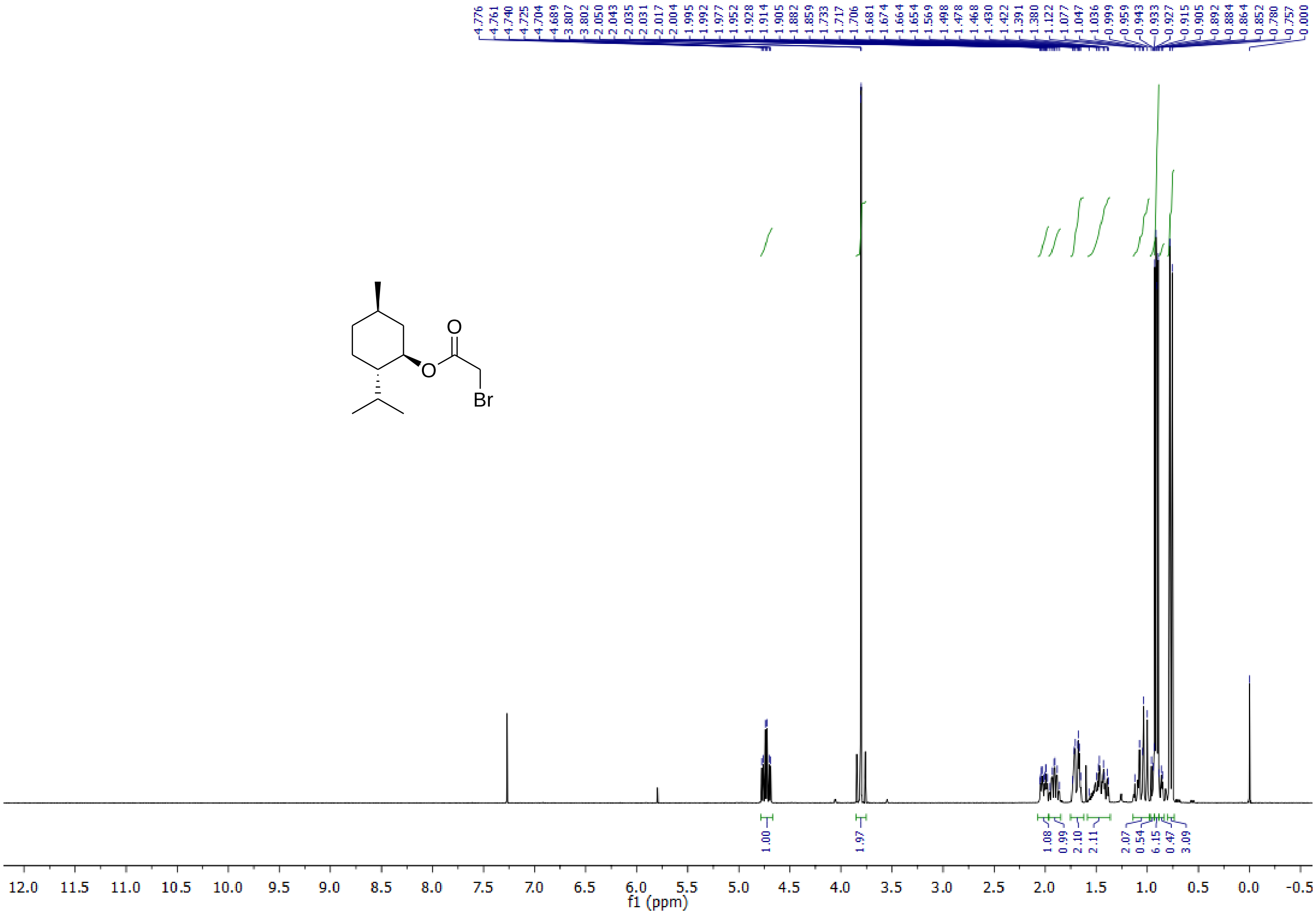
4.776
4.761
4.740
4.725
4.704
4.689
3.807
3.802
2.050
2.043
2.035
2.031
2.017
2.004
1.995
1.992
1.977
1.952
1.928
1.914
1.905
1.882
1.859
1.733
1.717
1.706
1.681
1.674
1.664
1.654
1.569
1.498
1.478
1.468
1.430
1.422
1.391
1.380
1.122
1.077
1.047
1.036
0.999
0.959
0.943
0.933
0.927
0.915
0.905
0.892
0.884
0.864
0.852
0.780
0.757
0.000
O
O
Br
1.00
1.97
1.08
0.99
2.10
2.11
2.07
0.54
6.15
0.47
3.09
12.0 11.5 11.0 10.5 10.0 9.5 9.0 8.5 8.0 7.5 7.0 6.5 6.0 5.5 5.0 4.5 4.0 3.5 3.0 2.5 2.0 1.5 1.0 0.5 0.0 -0.5
f1 (ppm)

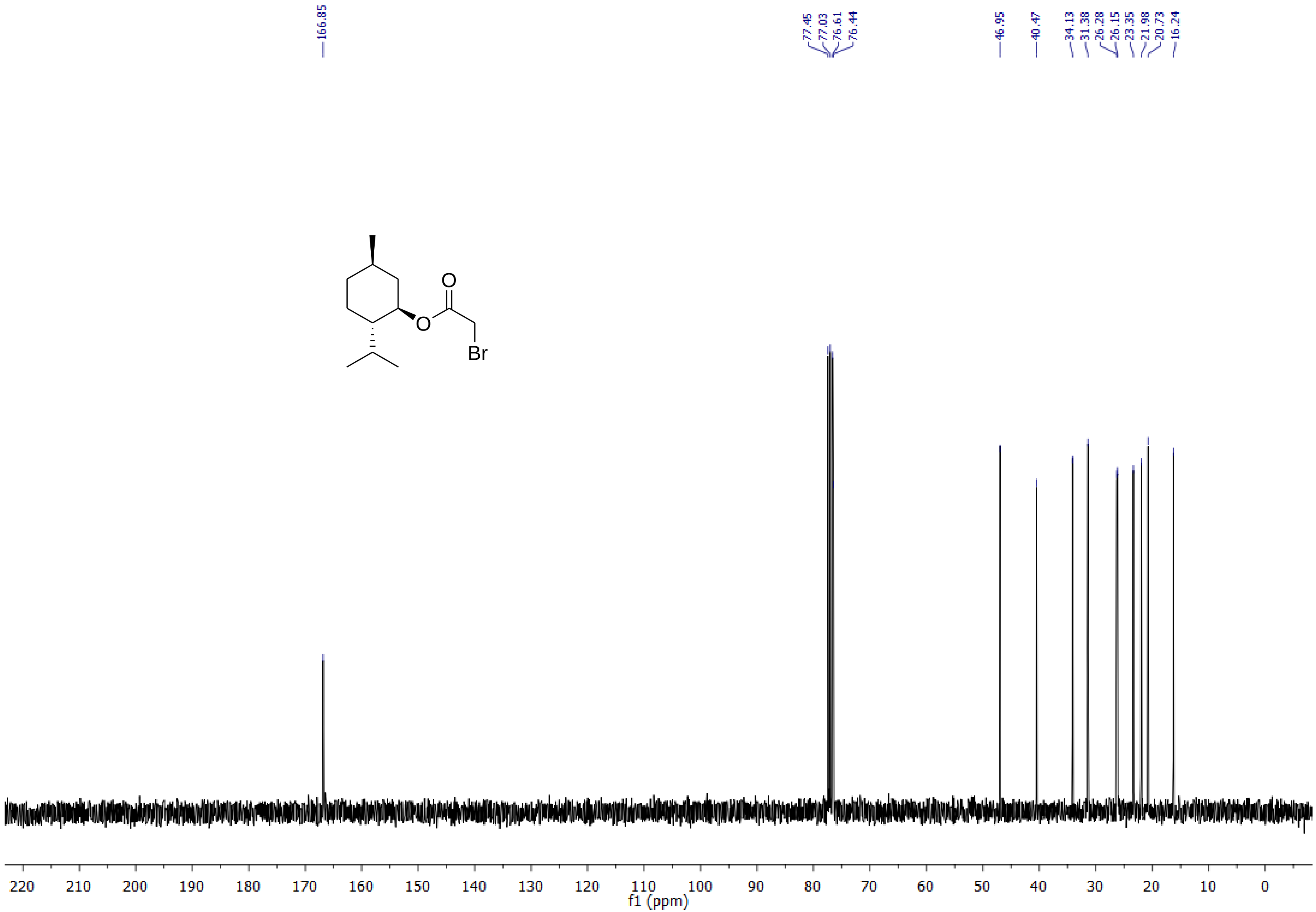
166.85
77.45
77.03
76.61
76.44
46.95
40.47
34.13
31.38
26.28
26.15
23.35
21.98
20.73
16.24
O
O
Br
220 210 200 190 180 170 160 150 140 130 120 110 100 90 80 70 60 50 40 30 20 10 0
f1 (ppm)

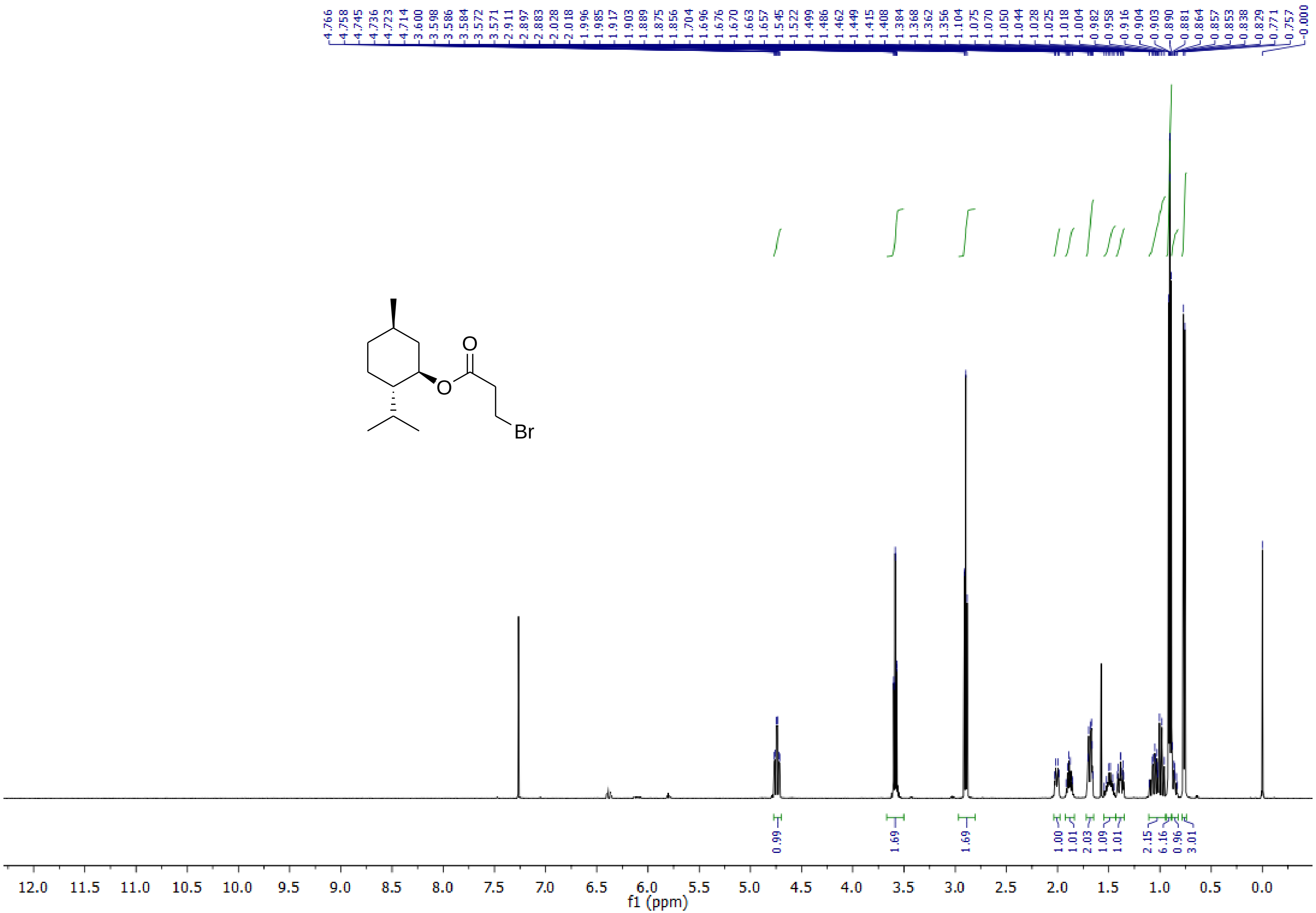
4.766 4.758 4.745 4.736 4.723 4.714 3.600 3.598 3.586 3.584 3.572 3.571 2.911 2.897 2.883 2.028 2.018 1.996 1.985 1.917 1.903 1.889 1.875 1.856 1.704 1.696 1.676 1.670 1.663 1.657 1.545 1.522 1.499 1.486 1.462 1.449 1.415 1.408 1.384 1.368 1.362 1.356 1.104 1.075 1.070 1.050 1.044 1.028 1.025 1.018 1.004 0.982 0.958 0.916 0.904 0.903 0.890 0.881 0.864 0.857 0.853 0.838 0.829 0.771 0.757 -0.000
O
O
Br
0.99
1.69
1.69
1.00
1.01
2.03
1.09
1.01
2.15
6.16
0.96
3.01
12.0 11.5 11.0 10.5 10.0 9.5 9.0 8.5 8.0 7.5 7.0 6.5 6.0 5.5 5.0 4.5 4.0 3.5 3.0 2.5 2.0 1.5 1.0 0.5 0.0
f1 (ppm)

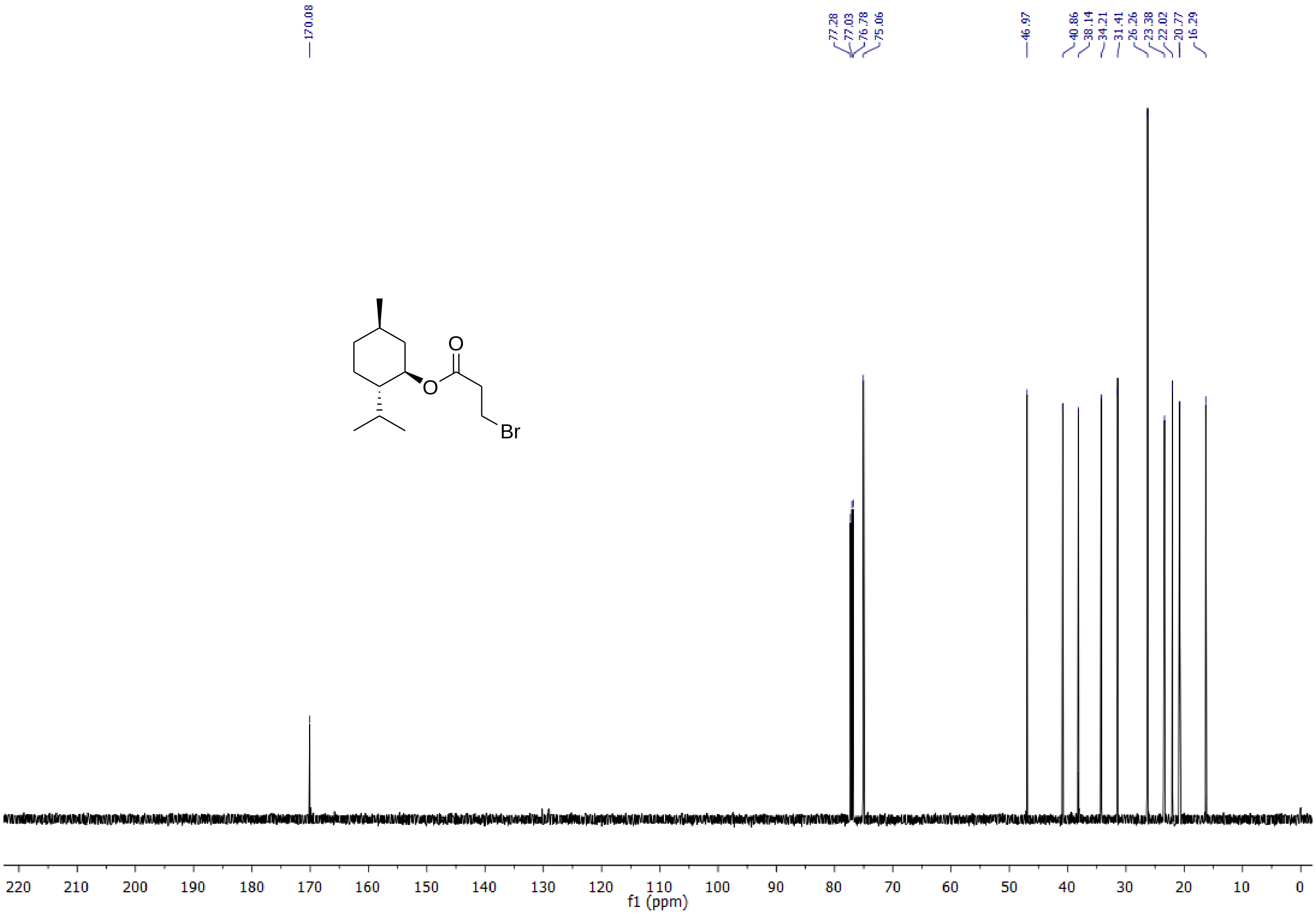

170.08
77.28
77.03
76.78
75.06
46.97
40.86
38.14
34.21
31.41
26.26
23.38
22.02
20.77
16.29
O
O
Br
220
210
200
190
180
170
160
150
140
130
120
110
100
90
80
70
60
50
40
30
20
10
0
f1 (ppm)

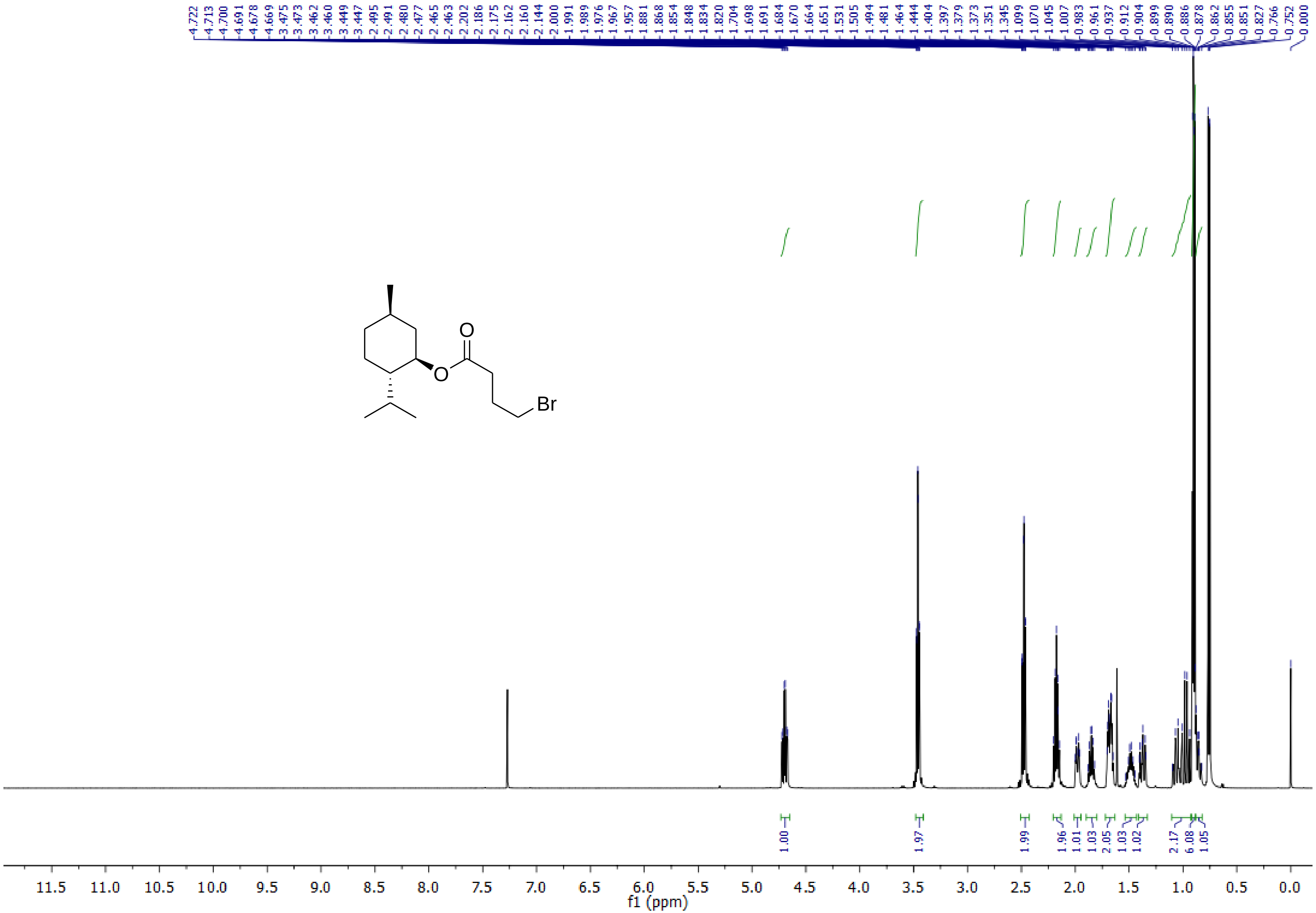

4.722
4.713
4.700
4.691
4.678
4.669
3.475
3.473
3.462
3.460
3.449
3.447
2.495
2.491
2.480
2.477
2.465
2.463
2.202
2.186
2.175
2.162
2.160
2.144
2.000
1.991
1.989
1.976
1.967
1.957
1.881
1.868
1.854
1.848
1.834
1.820
1.704
1.698
1.691
1.684
1.670
1.664
1.651
1.531
1.505
1.494
1.481
1.464
1.444
1.404
1.397
1.379
1.373
1.351
1.345
1.099
1.070
1.045
1.007
0.983
0.961
0.937
0.912
0.904
0.899
0.890
0.886
0.878
0.862
0.855
0.851
0.827
0.766
0.752
0.000
O
O
Br
1.00
1.97
1.99
1.96
1.01
1.03
2.05
1.03
1.02
2.17
6.08
1.05
11.5
11.0
10.5
10.0
9.5
9.0
8.5
8.0
7.5
7.0
6.5
6.0
5.5
5.0
4.5
4.0
3.5
3.0
2.5
2.0
1.5
1.0
0.5
0.0
f1 (ppm)

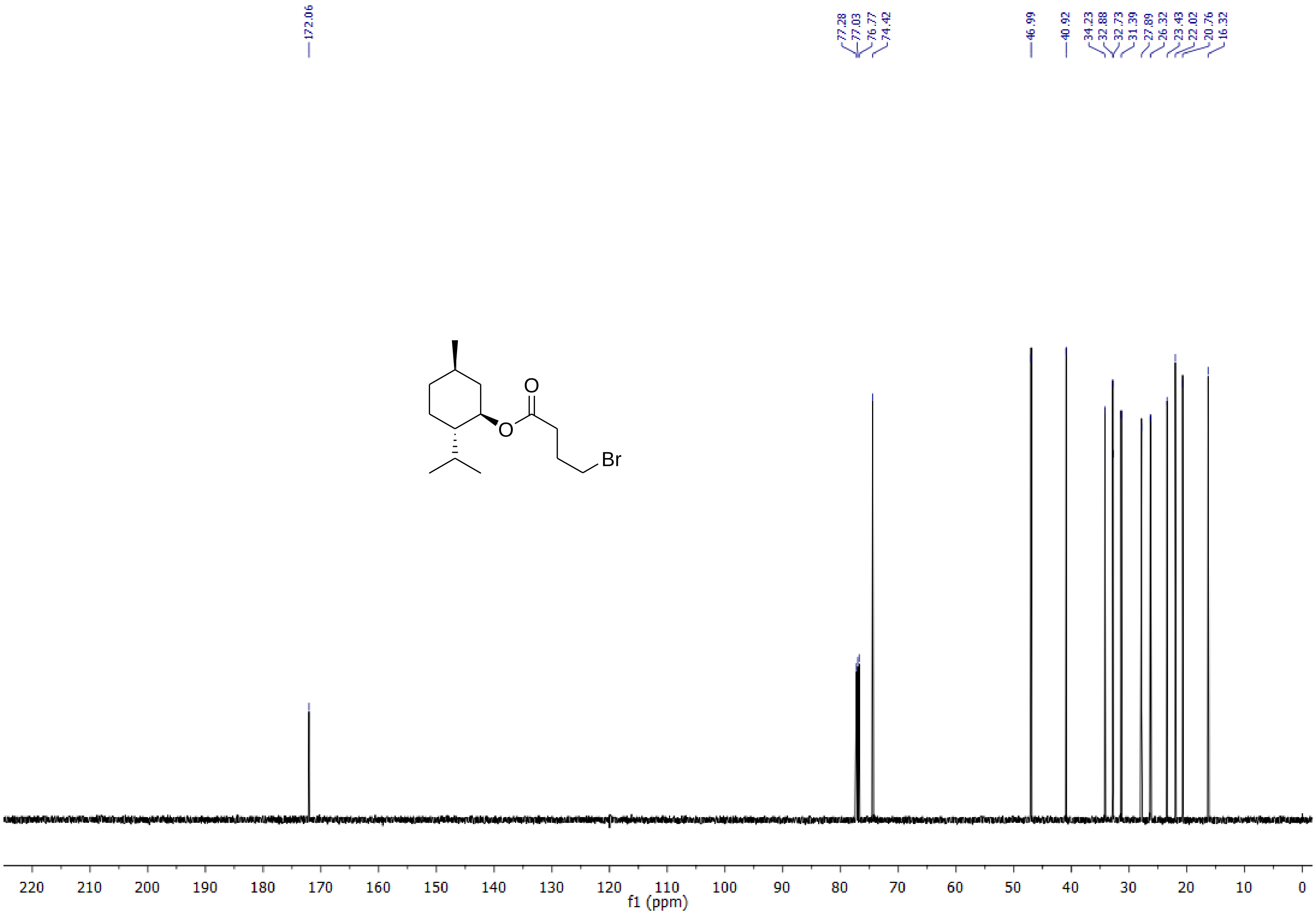

172.06
77.28
77.03
76.77
74.42
46.99
40.92
34.23
32.88
32.73
31.39
27.89
26.32
23.43
22.02
20.76
16.32
O
O
Br
220 210 200 190 180 170 160 150 140 130 120 110 100 90 80 70 60 50 40 30 20 10 0
f1 (ppm)

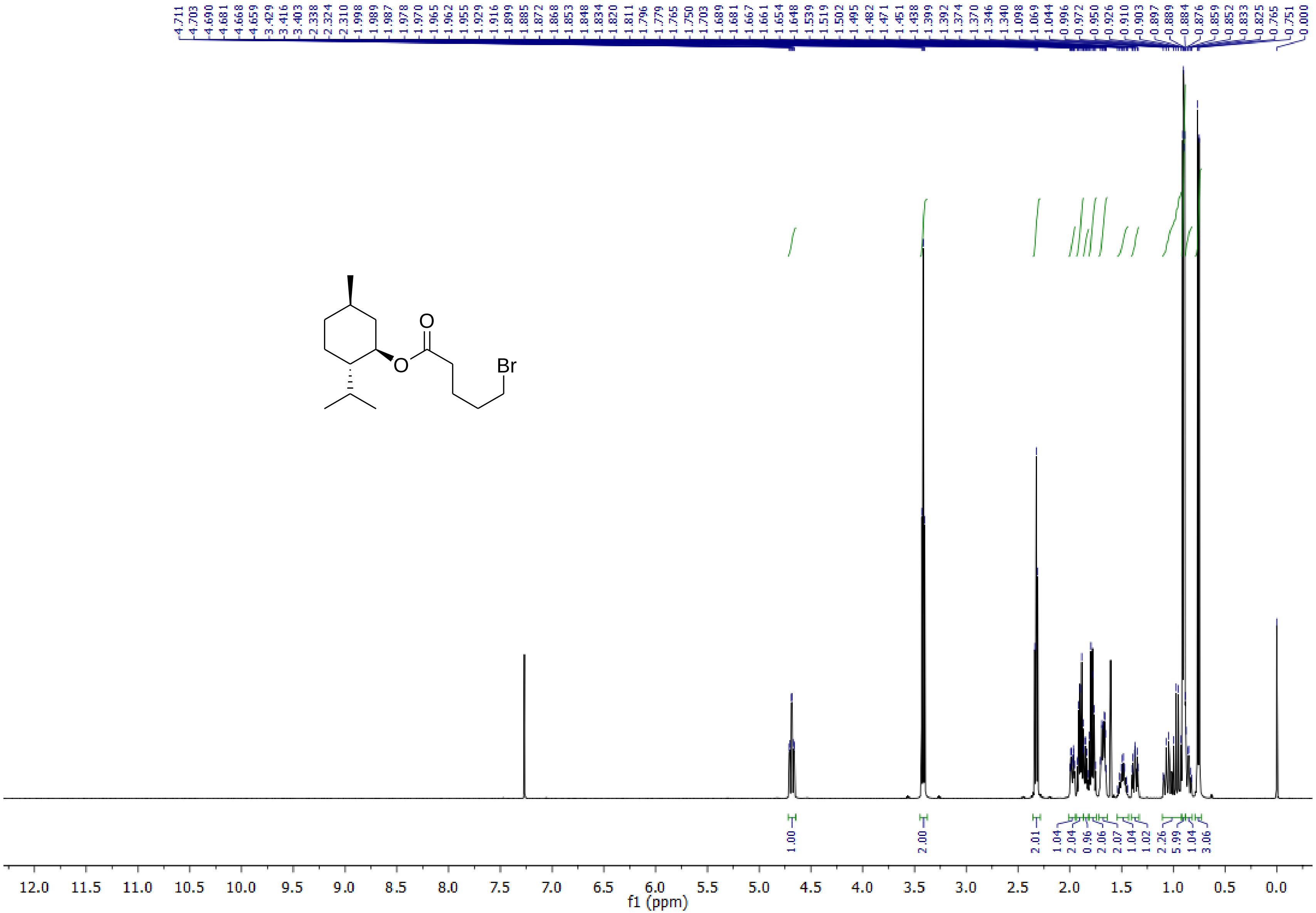
O
O
Br
f1 (ppm)

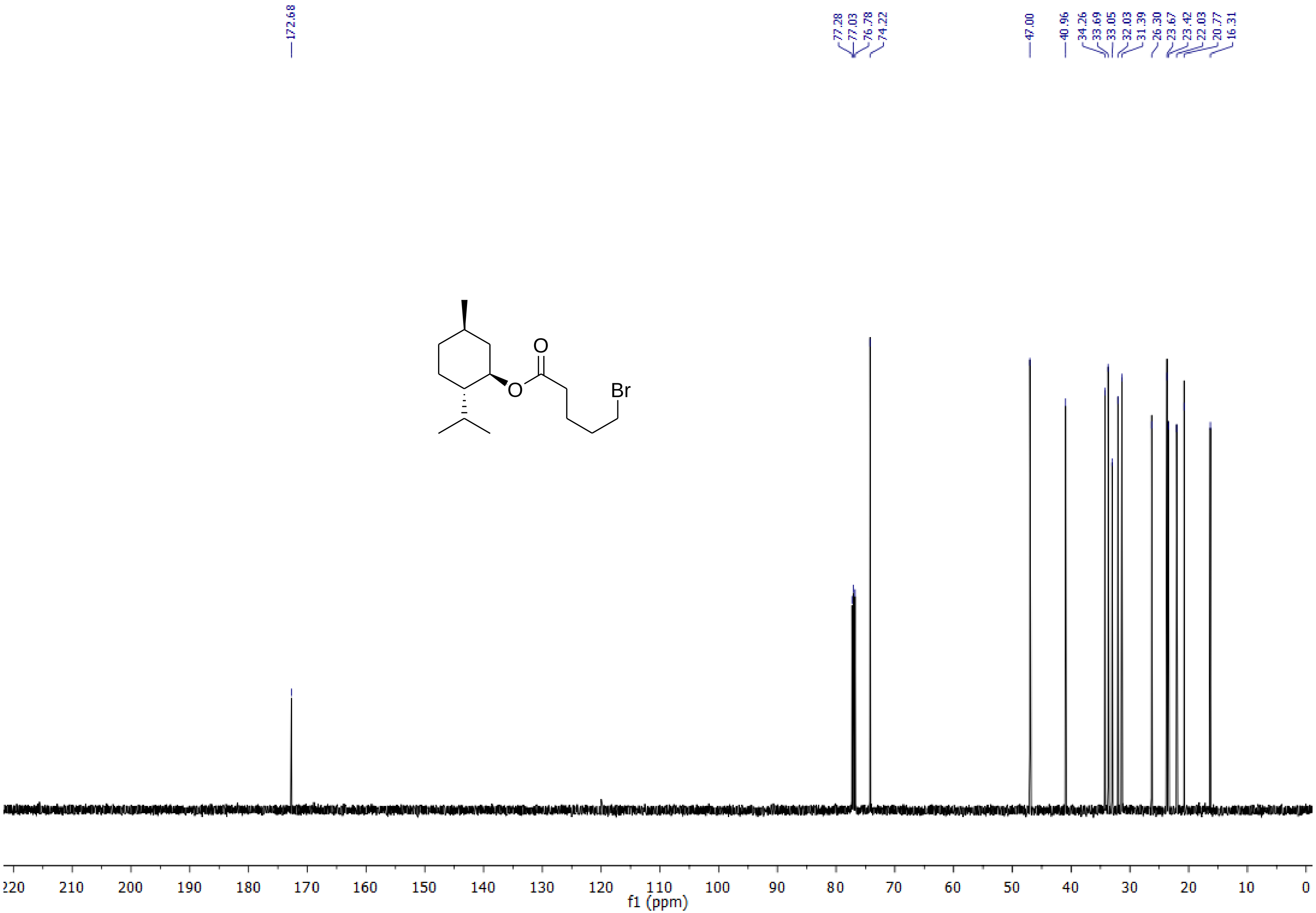
172.68
77.28
77.03
76.78
74.22
47.00
40.96
34.26
33.69
33.05
32.03
31.39
26.30
23.67
23.42
22.03
20.77
16.31
O
Br
220 210 200 190 180 170 160 150 140 130 120 110 100 90 80 70 60 50 40 30 20 10 0
f1 (ppm)

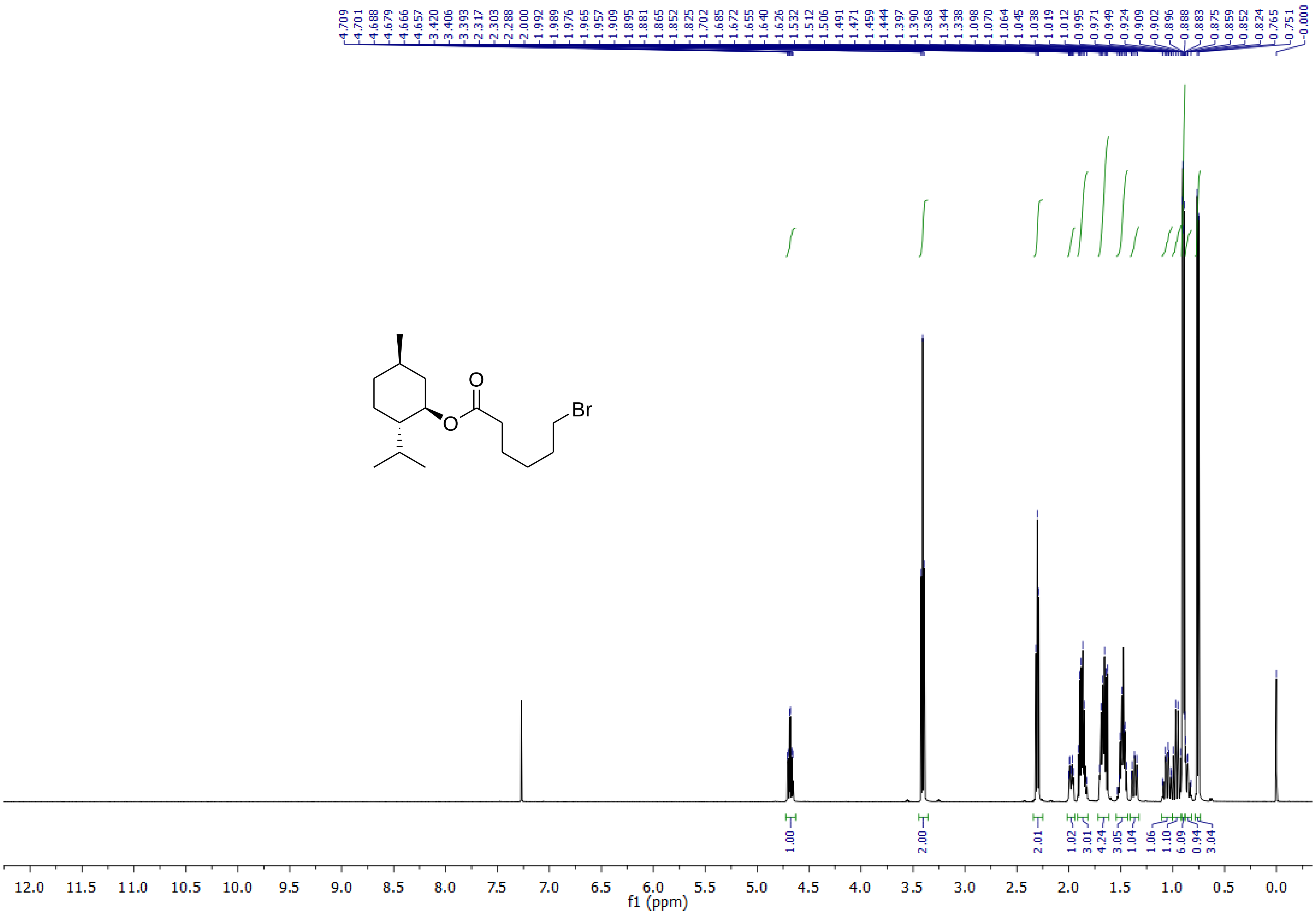
4.709 4.701 4.688 4.679 4.666 4.657 3.420 3.406 3.393 2.317 2.303 2.288 2.000 1.992 1.989 1.976 1.965 1.957 1.909 1.895 1.881 1.865 1.852 1.825 1.702 1.685 1.672 1.655 1.640 1.626 1.532 1.512 1.506 1.491 1.471 1.459 1.444 1.397 1.390 1.368 1.344 1.338 1.098 1.070 1.064 1.045 1.038 1.019 1.012 0.995 0.971 0.949 0.924 0.909 0.902 0.896 0.888 0.883 0.875 0.859 0.852 0.824 0.765 0.751 -0.000
O
O
Br
1.00
2.00
2.01
1.02
3.01
4.24
3.05
1.04
1.06
1.10
6.09
0.94
3.04
12.0 11.5 11.0 10.5 10.0 9.5 9.0 8.5 8.0 7.5 7.0 6.5 6.0 5.5 5.0 4.5 4.0 3.5 3.0 2.5 2.0 1.5 1.0 0.5 0.0
f1 (ppm)

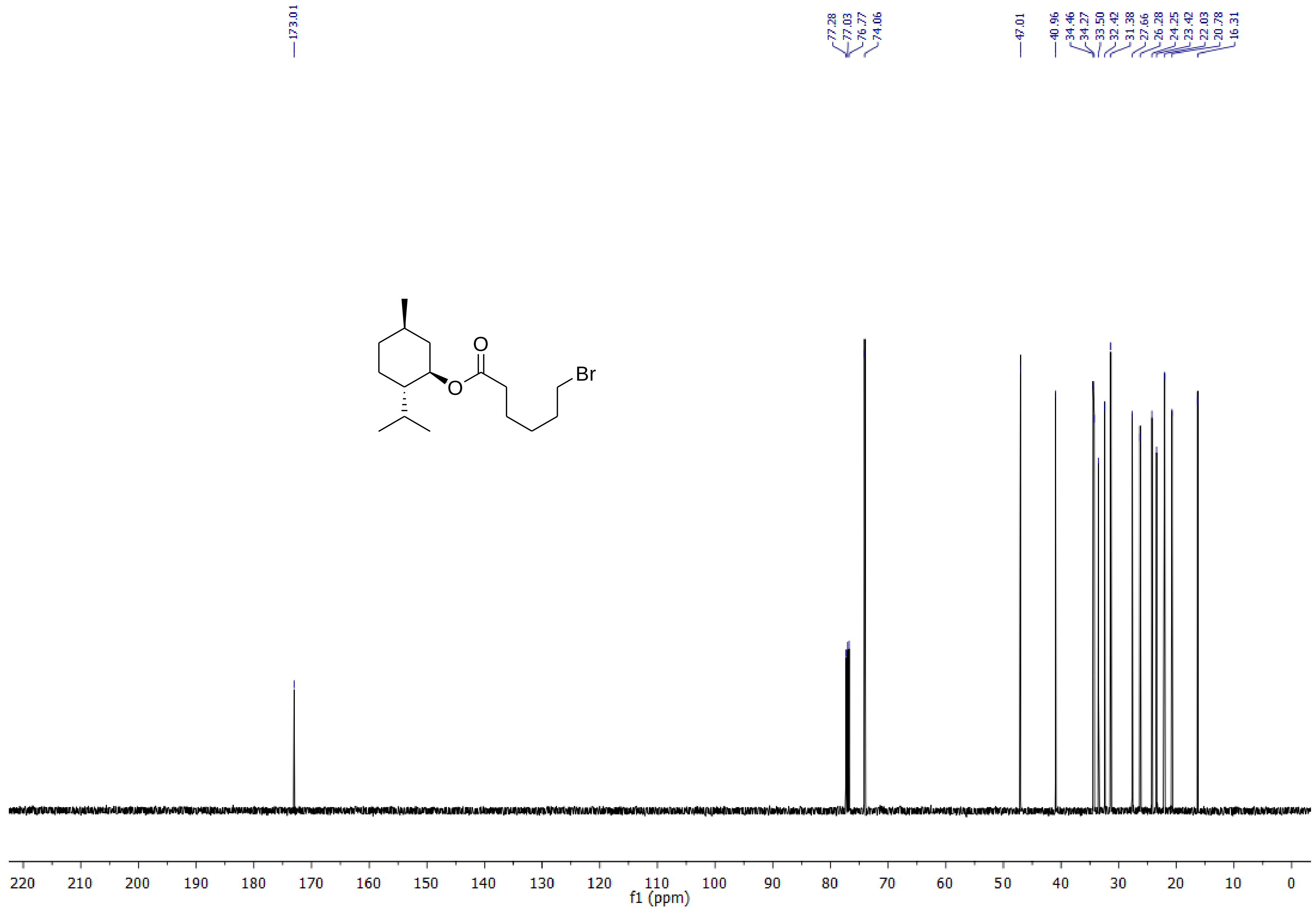
173.01
77.28
77.03
76.77
74.06
47.01
40.96
34.46
34.27
33.50
32.42
31.38
27.66
26.28
24.25
23.42
22.03
20.78
16.31
O
O
Br
220
210
200
190
180
170
160
150
140
130
120
110
100
90
80
70
60
50
40
30
20
10
0
f1 (ppm)

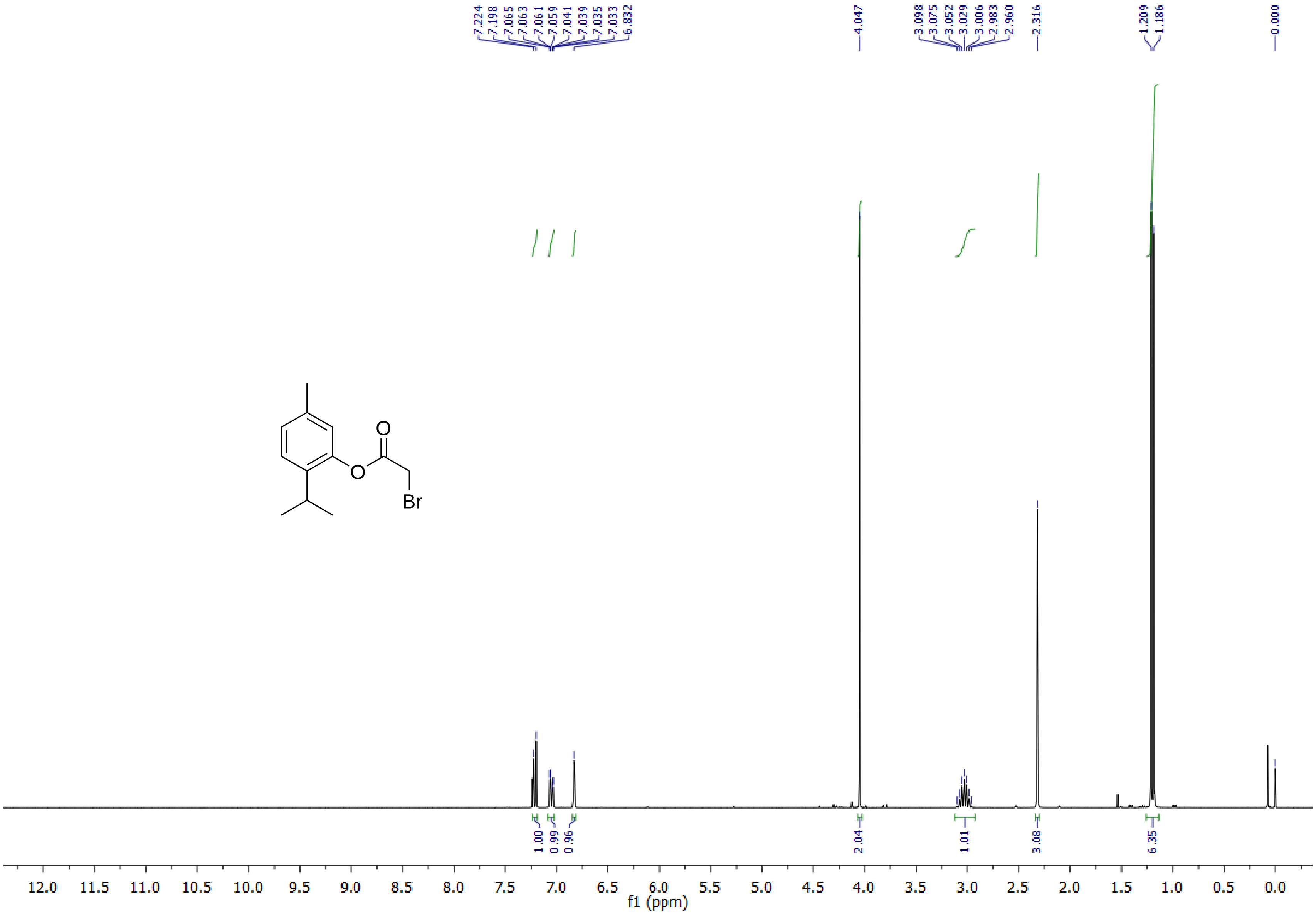
7.224
7.198
7.065
7.063
7.061
7.059
7.041
7.039
7.035
7.033
6.832
4.047
3.098
3.075
3.052
3.029
3.006
2.983
2.960
2.316
1.209
1.186
0.000
O
O
Br
1.00
0.99
0.96
2.04
1.01
3.08
6.35
12.0
11.5
11.0
10.5
10.0
9.5
9.0
8.5
8.0
7.5
7.0
6.5
6.0
5.5
5.0
4.5
4.0
3.5
3.0
2.5
2.0
1.5
1.0
0.5
0.0
f1 (ppm)

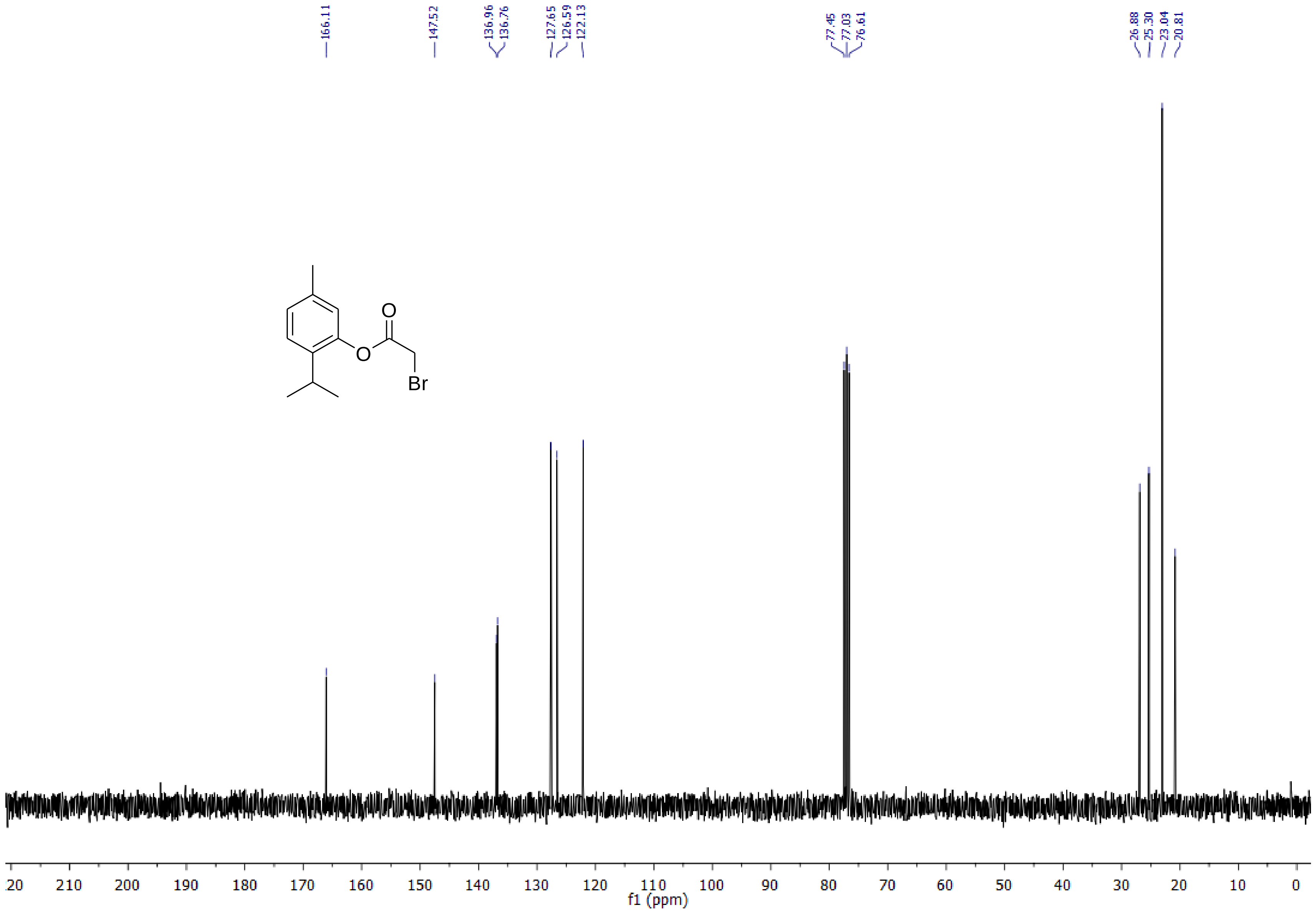

166.11
147.52
136.96
136.76
127.65
126.59
122.13
77.45
77.03
76.61
26.88
25.30
23.04
20.81
O
O
Br
220 210 200 190 180 170 160 150 140 130 120 110 100 90 80 70 60 50 40 30 20 10 0
f1 (ppm)

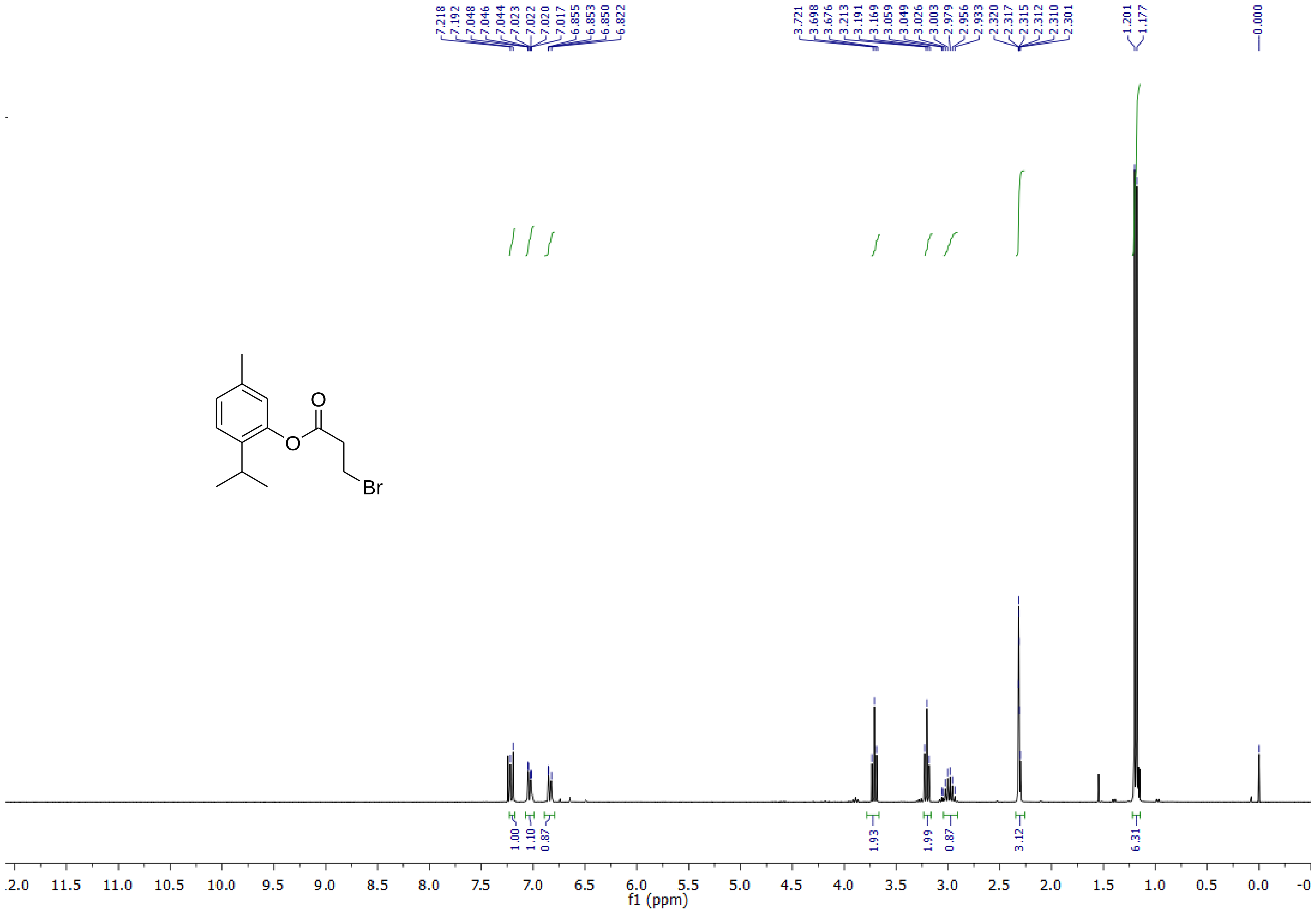
7.218
7.192
7.048
7.046
7.044
7.023
7.022
7.020
7.017
6.855
6.853
6.850
6.822
3.721
3.698
3.676
3.213
3.191
3.169
3.059
3.049
3.026
3.003
2.979
2.956
2.933
2.320
2.317
2.315
2.312
2.310
2.301
1.201
1.177
0.000
O
O
Br
1.00
1.10
0.87
1.93
1.99
0.87
3.12
6.31
f1 (ppm)

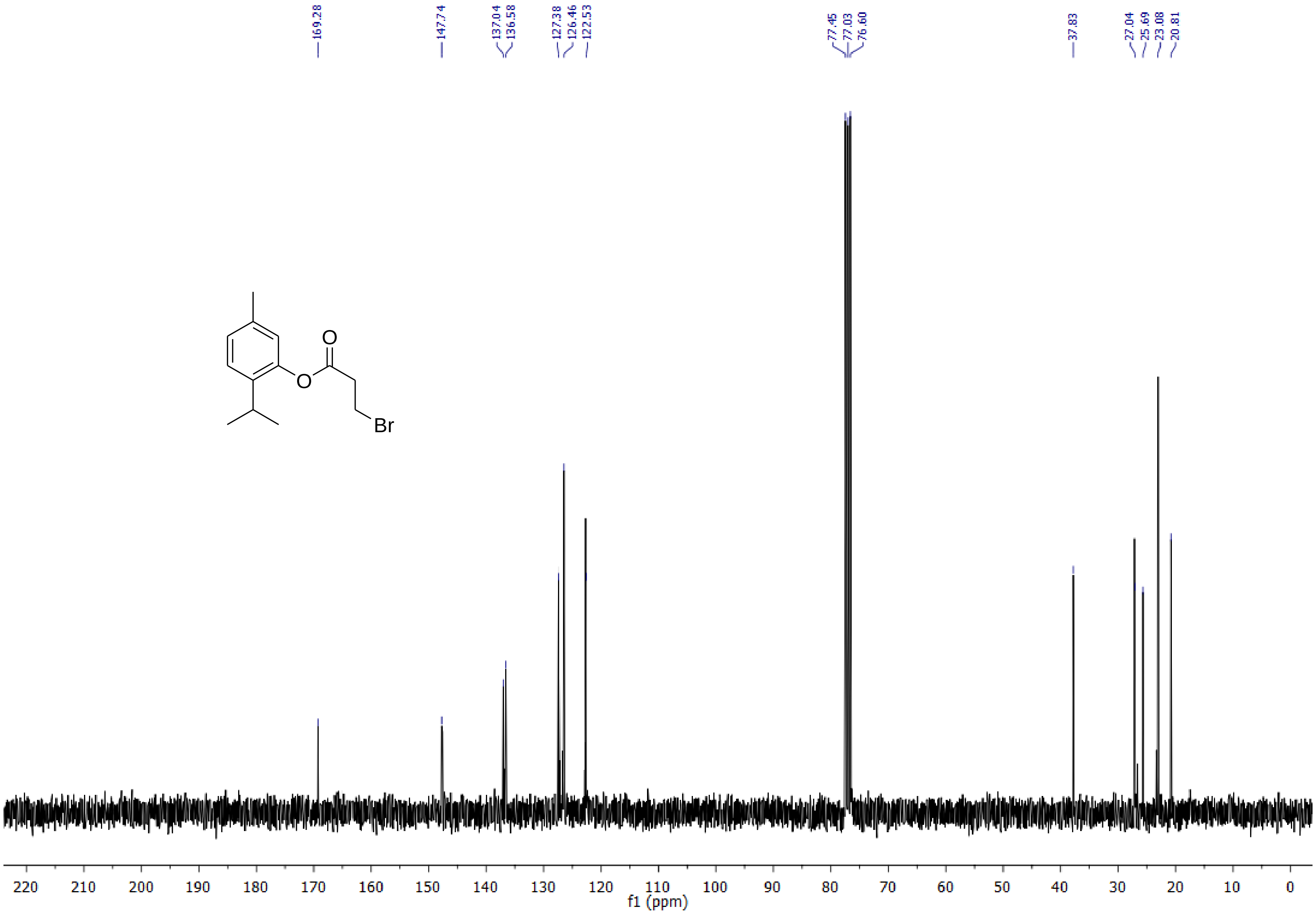
169.28
147.74
137.04
136.58
127.38
126.46
122.53
77.45
77.03
76.60
37.83
27.04
25.69
23.08
20.81
O
O
Br
220
210
200
190
180
170
160
150
140
130
120
110
100
90
80
70
60
50
40
30
20
10
0
f1 (ppm)

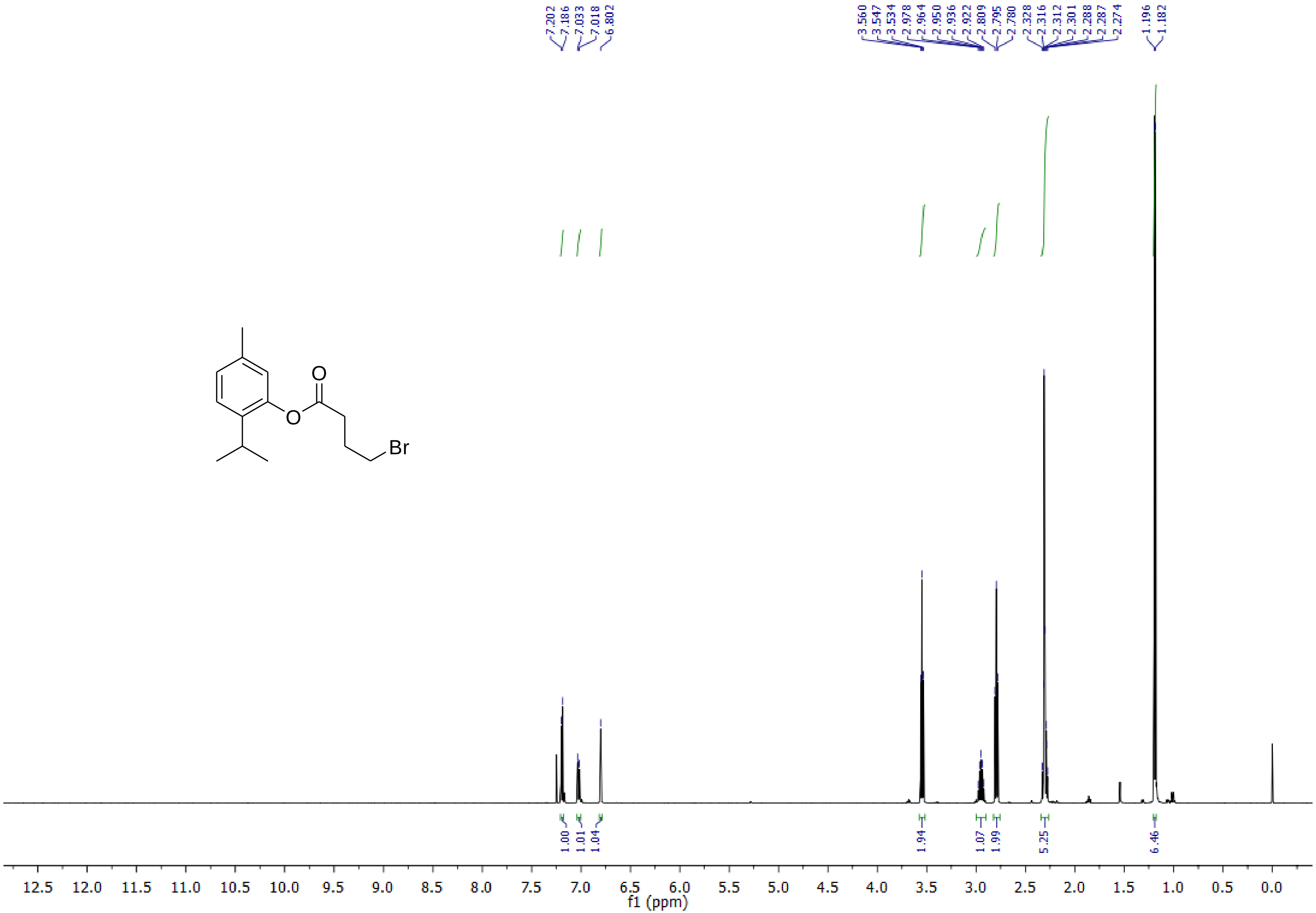

7.202
7.186
7.033
7.018
6.802
3.560
3.547
3.534
2.978
2.964
2.950
2.936
2.922
2.809
2.795
2.780
2.328
2.316
2.312
2.301
2.288
2.287
2.274
1.196
1.182
O
O
Br
1.00
1.01
1.04
1.94
1.07
1.99
5.25
6.46
12.5 12.0 11.5 11.0 10.5 10.0 9.5 9.0 8.5 8.0 7.5 7.0 6.5 6.0 5.5 5.0 4.5 4.0 3.5 3.0 2.5 2.0 1.5 1.0 0.5 0.0
f1 (ppm)

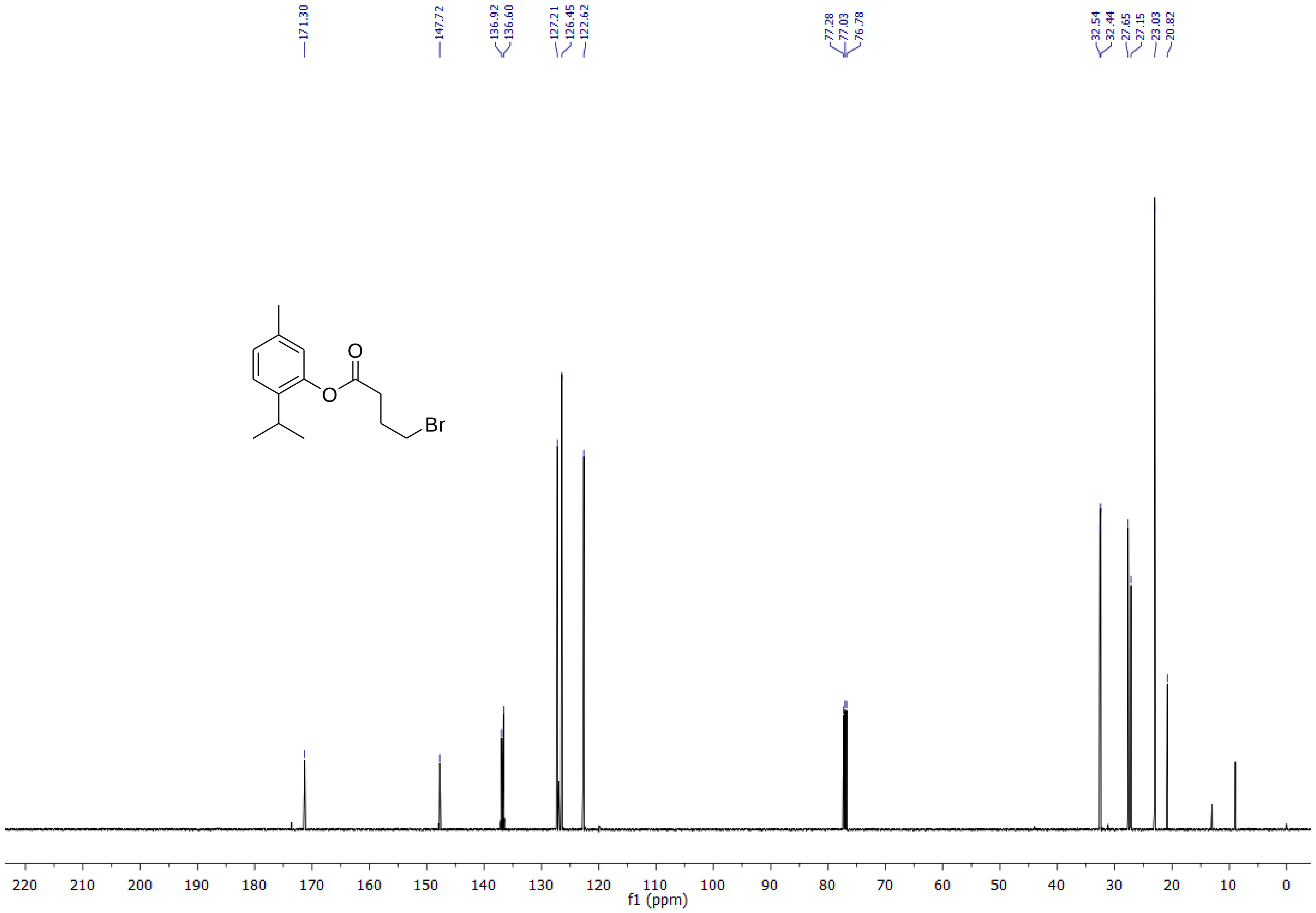
171.30
147.72
136.92
136.60
127.21
126.45
122.62
77.28
77.03
76.78
32.54
32.44
27.65
27.15
23.03
20.82
O
O
Br
220 210 200 190 180 170 160 150 140 130 120 110 100 90 80 70 60 50 40 30 20 10 0
f1 (ppm)

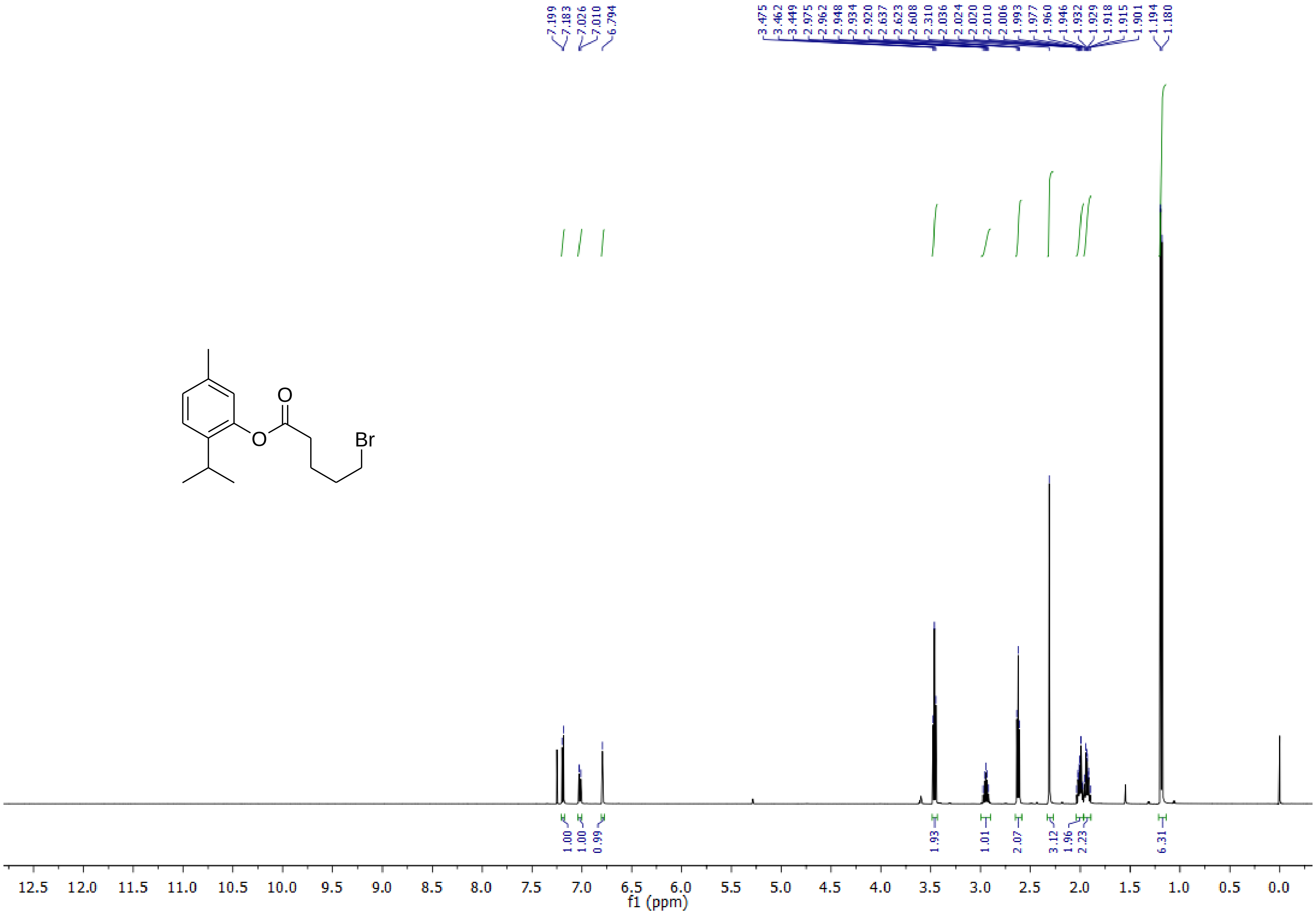
7.199
7.183
7.026
7.010
6.794
3.475
3.462
3.449
2.975
2.962
2.948
2.934
2.920
2.637
2.623
2.608
2.310
2.036
2.024
2.020
2.010
2.006
1.993
1.977
1.960
1.946
1.932
1.929
1.918
1.915
1.901
1.194
1.180
1.00
1.00
0.99
1.93
1.01
2.07
3.12
1.96
2.23
6.31
O
O
Br
f1 (ppm)

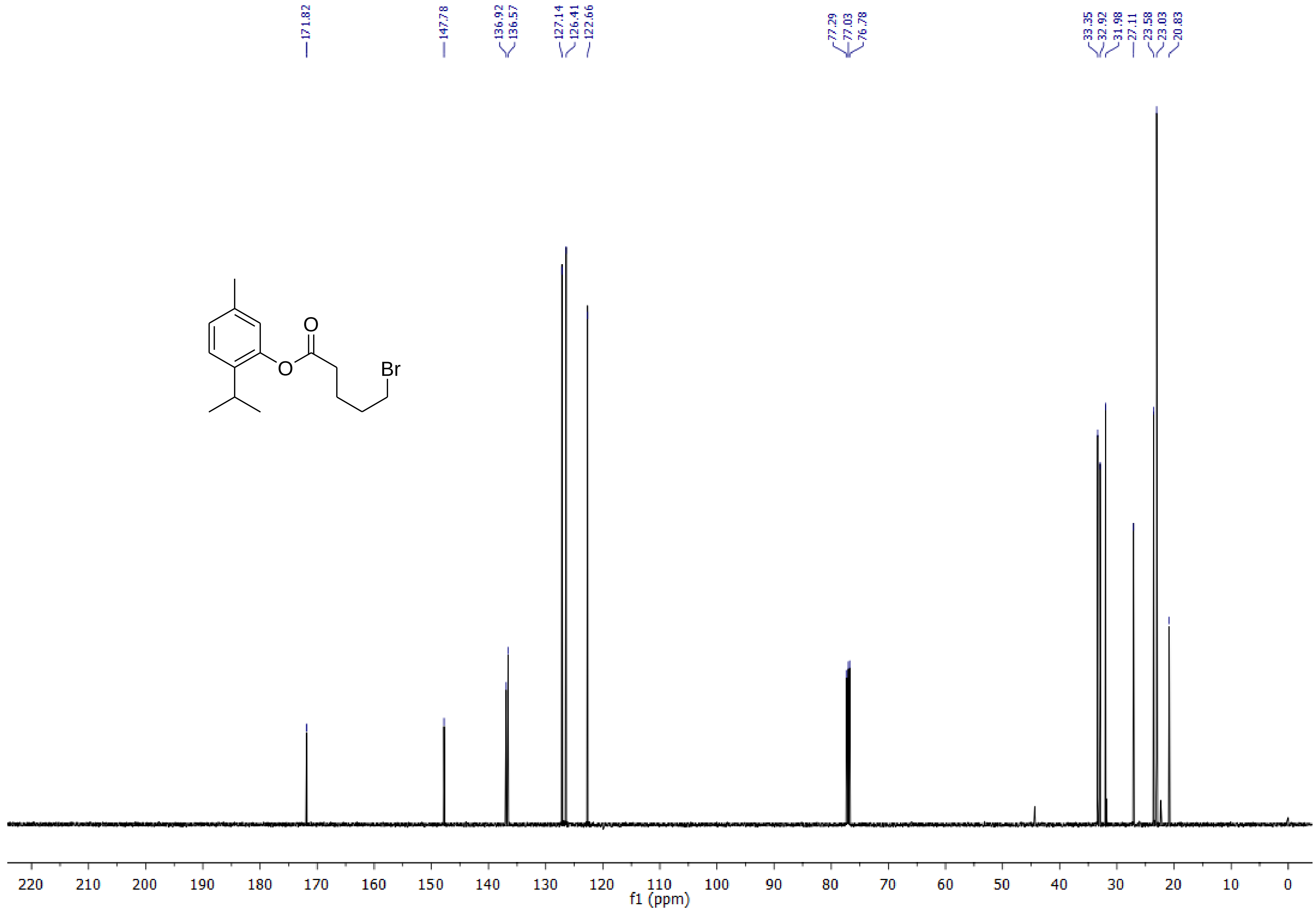
171.82
147.78
136.92
136.57
127.14
126.41
122.66
77.29
77.03
76.78
33.35
32.92
31.98
27.11
23.58
23.03
20.83
O
O
Br
220
210
200
190
180
170
160
150
140
130
120
110
100
90
80
70
60
50
40
30
20
10
0
f1 (ppm)

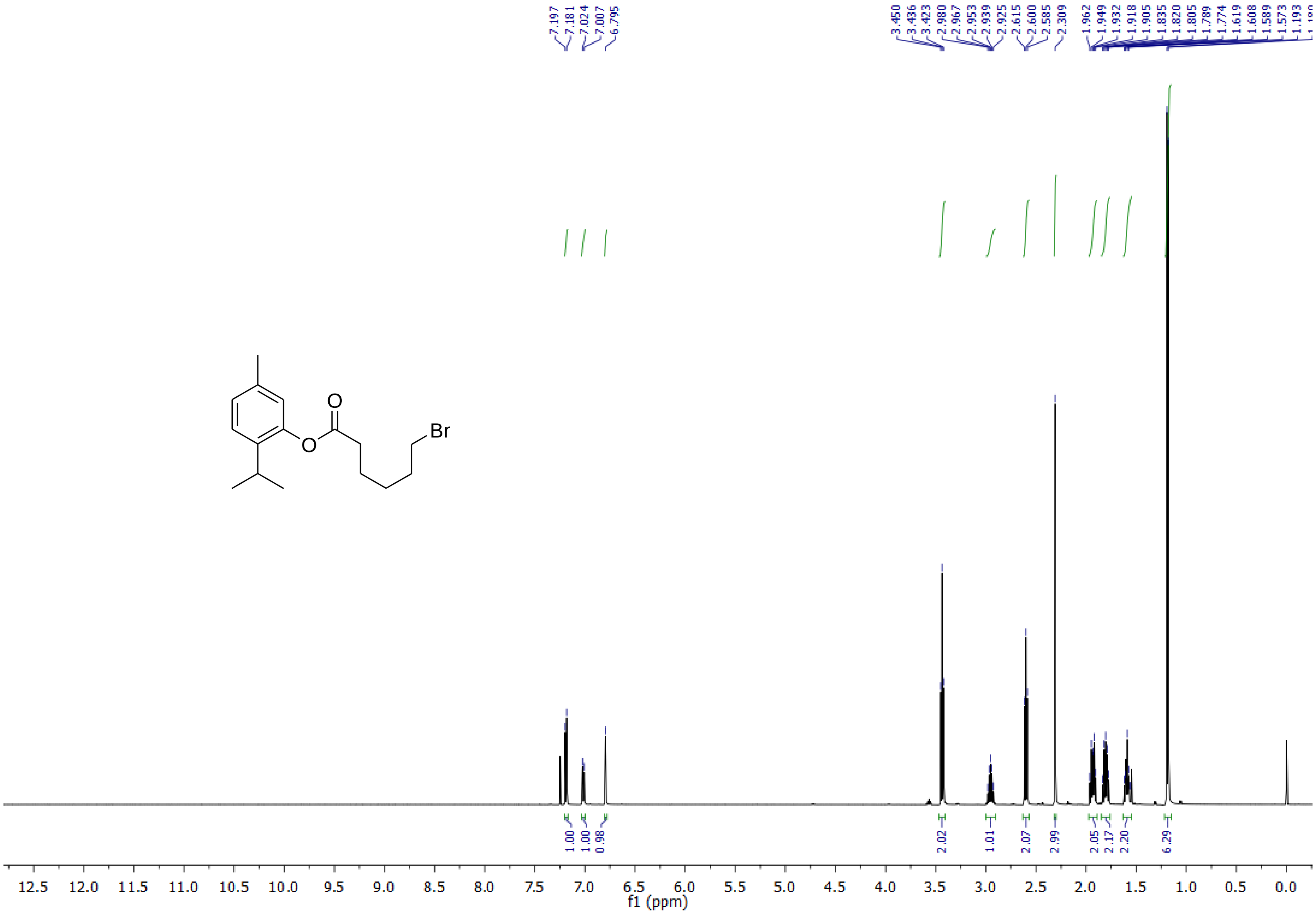

7.197
7.181
7.024
7.007
6.795
3.450
3.436
3.423
2.980
2.967
2.953
2.939
2.925
2.615
2.600
2.585
2.309
1.962
1.949
1.932
1.918
1.905
1.835
1.820
1.805
1.789
1.774
1.619
1.608
1.589
1.573
1.193
O
O
Br
1.00
1.00
0.98
2.02
1.01
2.07
2.99
2.05
2.17
2.20
6.29
12.5 12.0 11.5 11.0 10.5 10.0 9.5 9.0 8.5 8.0 7.5 7.0 6.5 6.0 5.5 5.0 4.5 4.0 3.5 3.0 2.5 2.0 1.5 1.0 0.5 0.0
f1 (ppm)

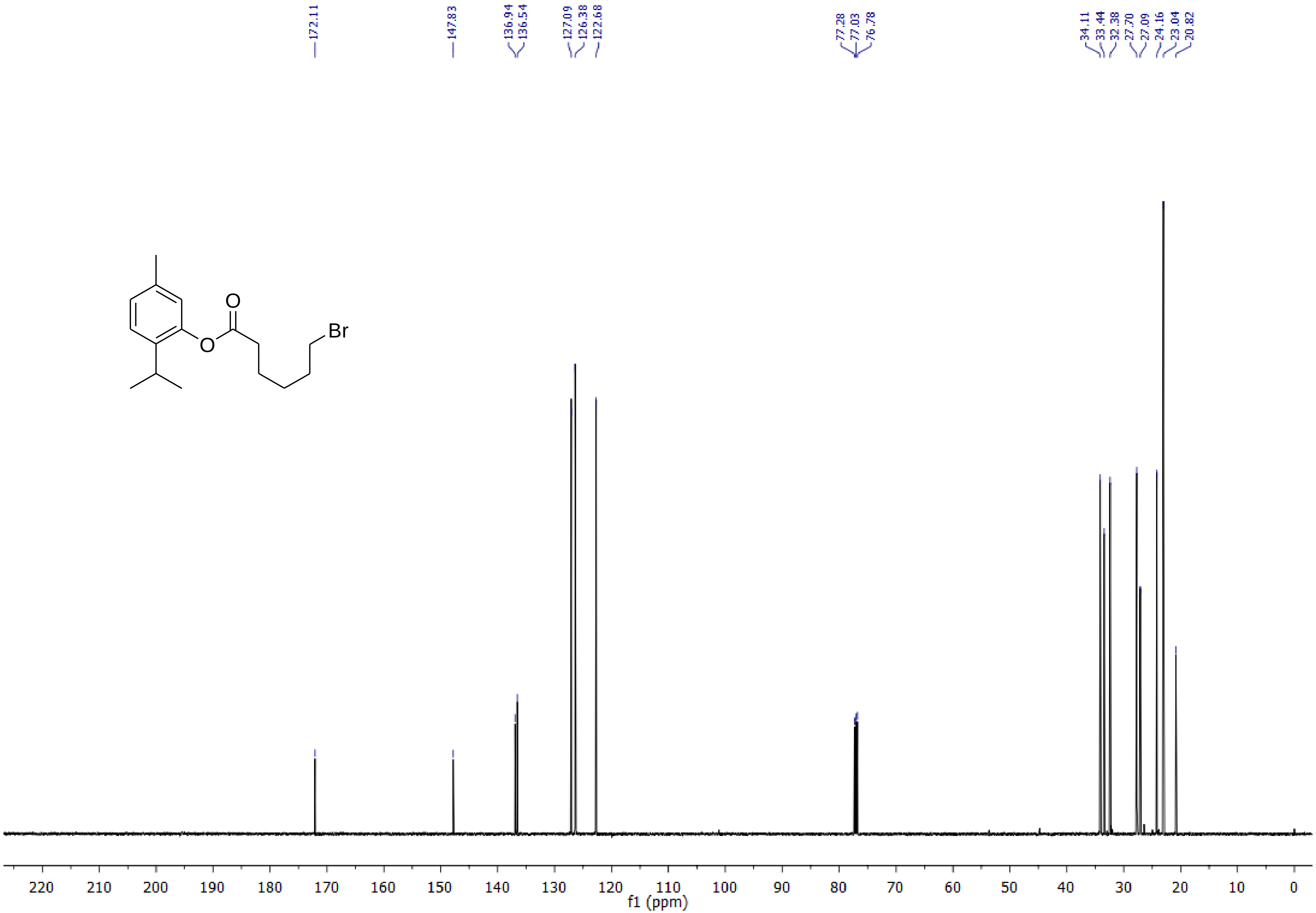

172.11
147.83
136.94
136.54
127.09
126.38
122.68
77.28
77.03
76.78
34.11
33.44
32.38
27.70
27.09
24.16
23.04
20.82
O
O
Br
220
210
200
190
180
170
160
150
140
130
120
110
100
90
80
70
60
50
40
30
20
10
0
f1 (ppm)

4. The NMR spectra of ciprofloxacin conjugates in order: **1**, **9**; **2-6**; **10-14**; **7-8**; **15-16**.

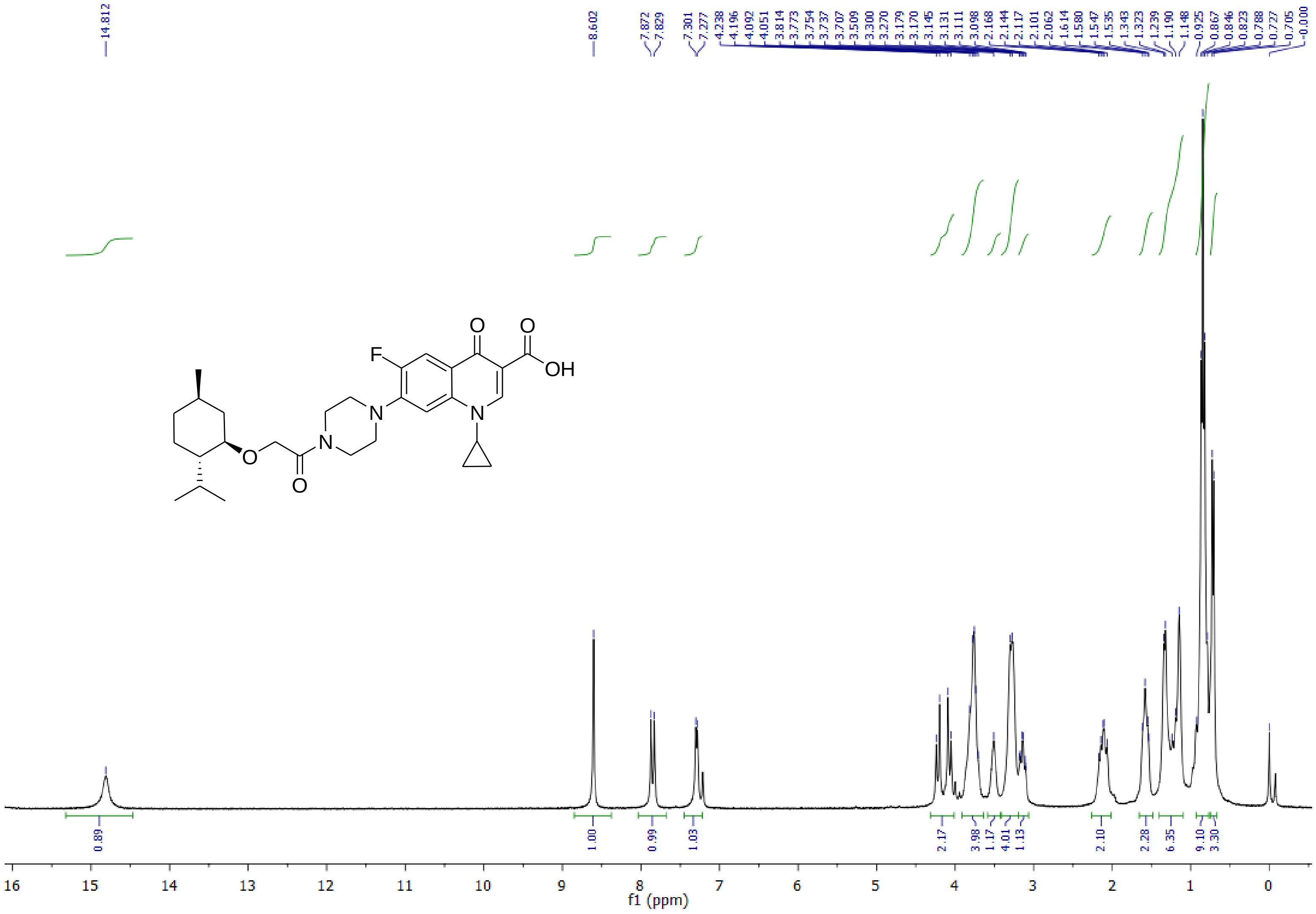

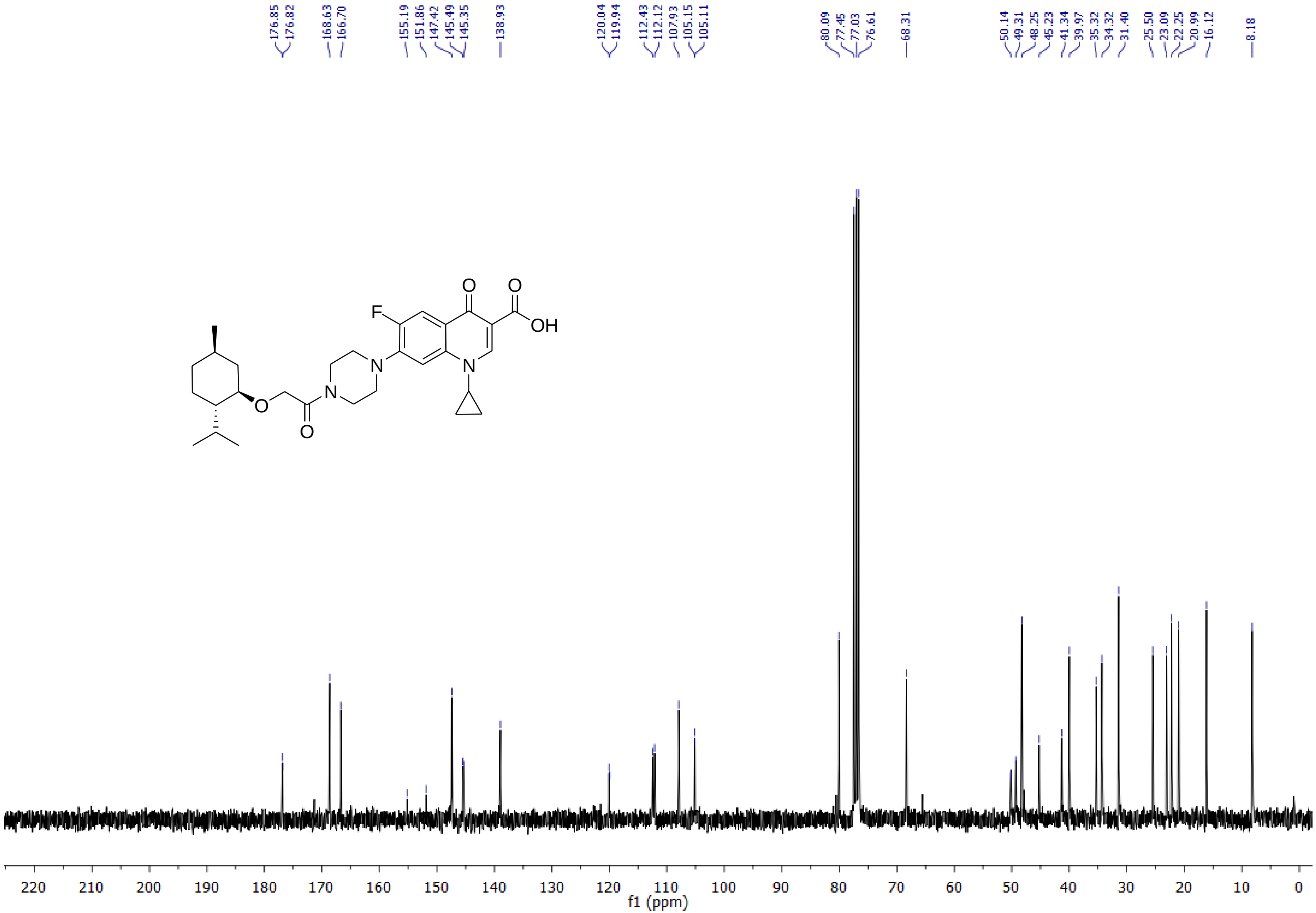

176.85
176.82
168.63
166.70
155.19
151.86
147.42
145.49
145.35
138.93
120.04
119.94
112.43
112.12
107.93
105.15
105.11
80.09
77.45
77.03
76.61
68.31
50.14
49.31
48.25
45.23
41.34
39.97
35.32
34.32
31.40
25.50
23.09
22.25
20.99
16.12
8.18
F
O
O
OH
N
N
N
O
O
220
210
200
190
180
170
160
150
140
130
120
110
100
90
80
70
60
50
40
30
20
10
0
f1 (ppm)

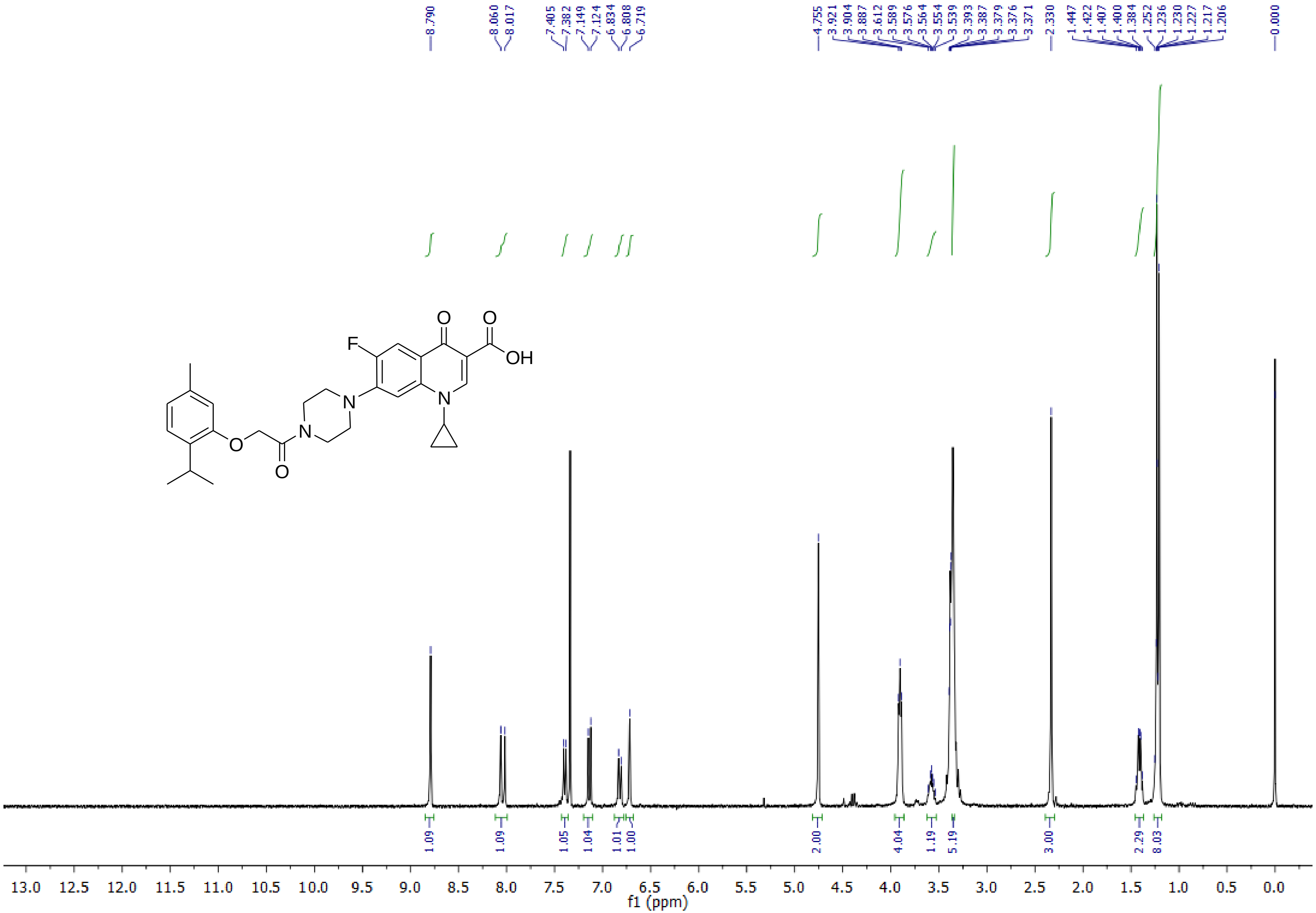
8.790
8.060
8.017
7.405
7.382
7.149
7.124
6.834
6.808
6.719
4.755
3.921
3.904
3.887
3.612
3.589
3.576
3.564
3.554
3.539
3.393
3.387
3.379
3.376
3.371
2.330
1.447
1.422
1.407
1.400
1.384
1.252
1.236
1.230
1.227
1.217
1.206
0.000
1.09
1.09
1.05
1.04
1.01
1.00
2.00
4.04
1.19
5.19
3.00
2.29
8.03
13.0 12.5 12.0 11.5 11.0 10.5 10.0 9.5 9.0 8.5 8.0 7.5 7.0 6.5 6.0 5.5 5.0 4.5 4.0 3.5 3.0 2.5 2.0 1.5 1.0 0.5 0.0
f1 (ppm)
F
O
O
OH
N
N
N
O
O

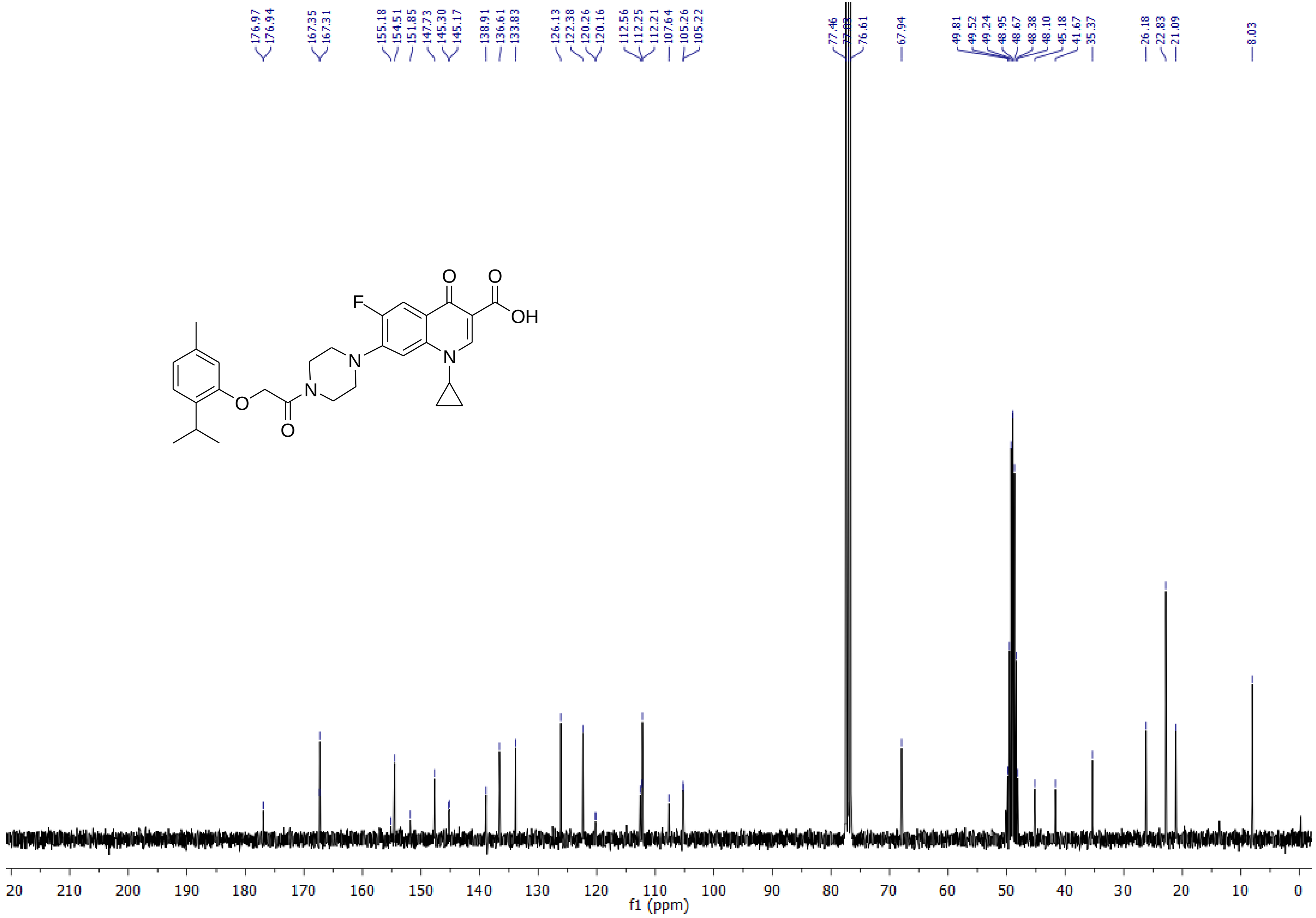

176.97
176.94
167.35
167.31
155.18
154.51
151.85
147.73
145.30
145.17
138.91
136.61
133.83
126.13
122.38
120.26
120.16
112.56
112.25
112.21
107.64
105.26
105.22
77.46
77.03
76.61
67.94
49.81
49.52
49.24
48.95
48.67
48.38
48.10
45.18
41.67
35.37
26.18
22.83
21.09
8.03
F
O
O
OH
N
N
N
O
O
20
210
200
190
180
170
160
150
140
130
120
110
100
90
80
70
60
50
40
30
20
10
0
f1 (ppm)

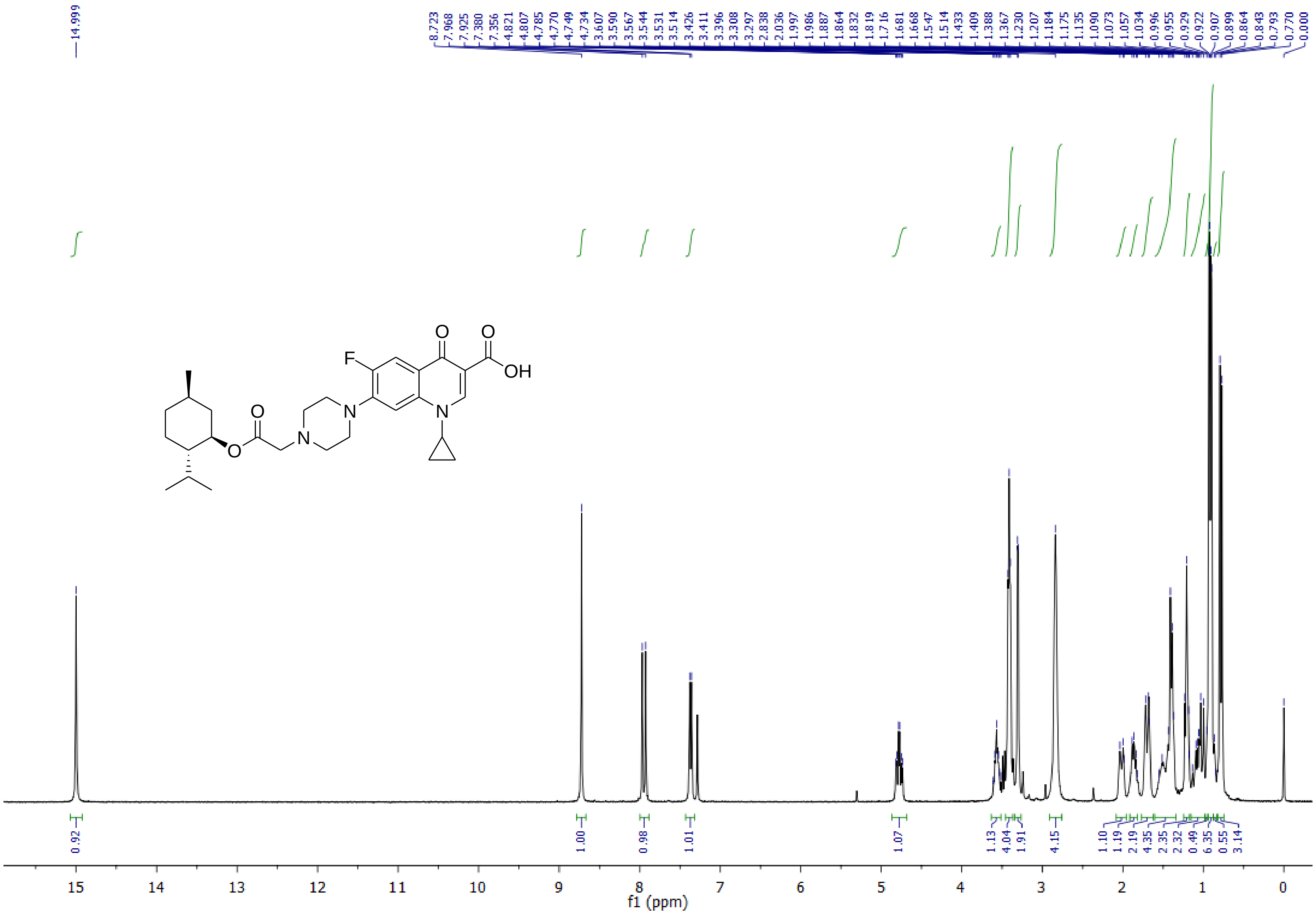
14.999
8.723
7.968
7.925
7.380
7.356
4.821
4.807
4.785
4.770
4.749
4.734
3.607
3.590
3.567
3.544
3.531
3.514
3.426
3.411
3.396
3.308
3.297
2.838
2.036
1.997
1.986
1.887
1.864
1.832
1.819
1.716
1.681
1.668
1.547
1.514
1.433
1.409
1.388
1.367
1.230
1.207
1.184
1.175
1.135
1.090
1.073
1.057
1.034
0.996
0.955
0.929
0.922
0.907
0.899
0.864
0.843
0.793
0.770
0.000
F
O
O
OH
N
N
N
O
O
0.92
1.00
0.98
1.01
1.07
1.13
4.04
1.91
4.15
1.10
1.19
2.19
4.35
2.35
2.32
0.49
6.35
0.55
3.14
15
14
13
12
11
10
9
8
7
6
5
4
3
2
1
0
f1 (ppm)

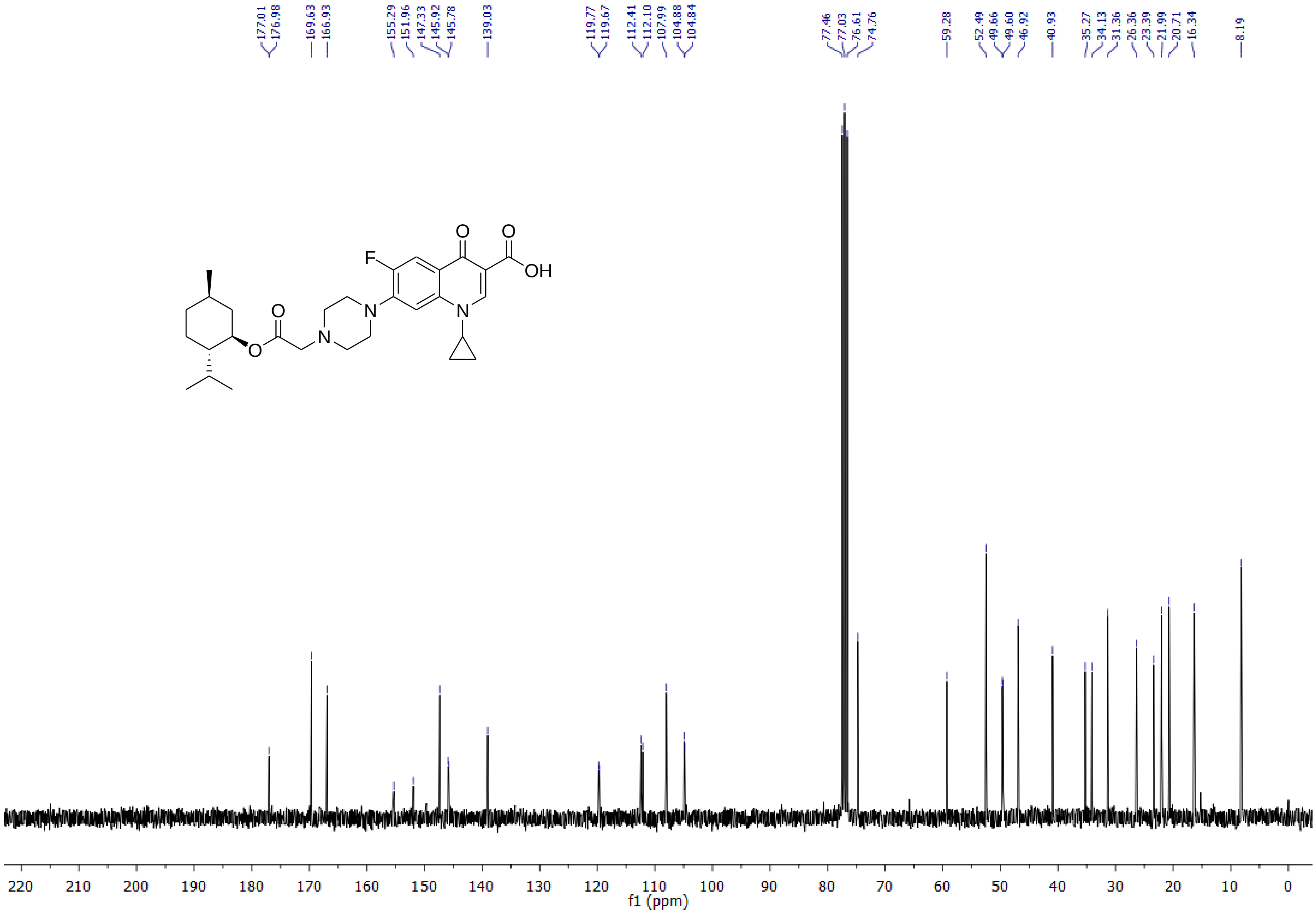
177.01
176.98
169.63
166.93
155.29
151.96
147.33
145.92
145.78
139.03
119.77
119.67
112.41
112.10
107.99
104.88
104.84
77.46
77.03
76.61
74.76
59.28
52.49
49.66
49.60
46.92
40.93
35.27
34.13
31.36
26.36
23.39
21.99
20.71
16.34
8.19
F
O
O
OH
N
N
N
O
O
220 210 200 190 180 170 160 150 140 130 120 110 100 90 80 70 60 50 40 30 20 10 0
f1 (ppm)

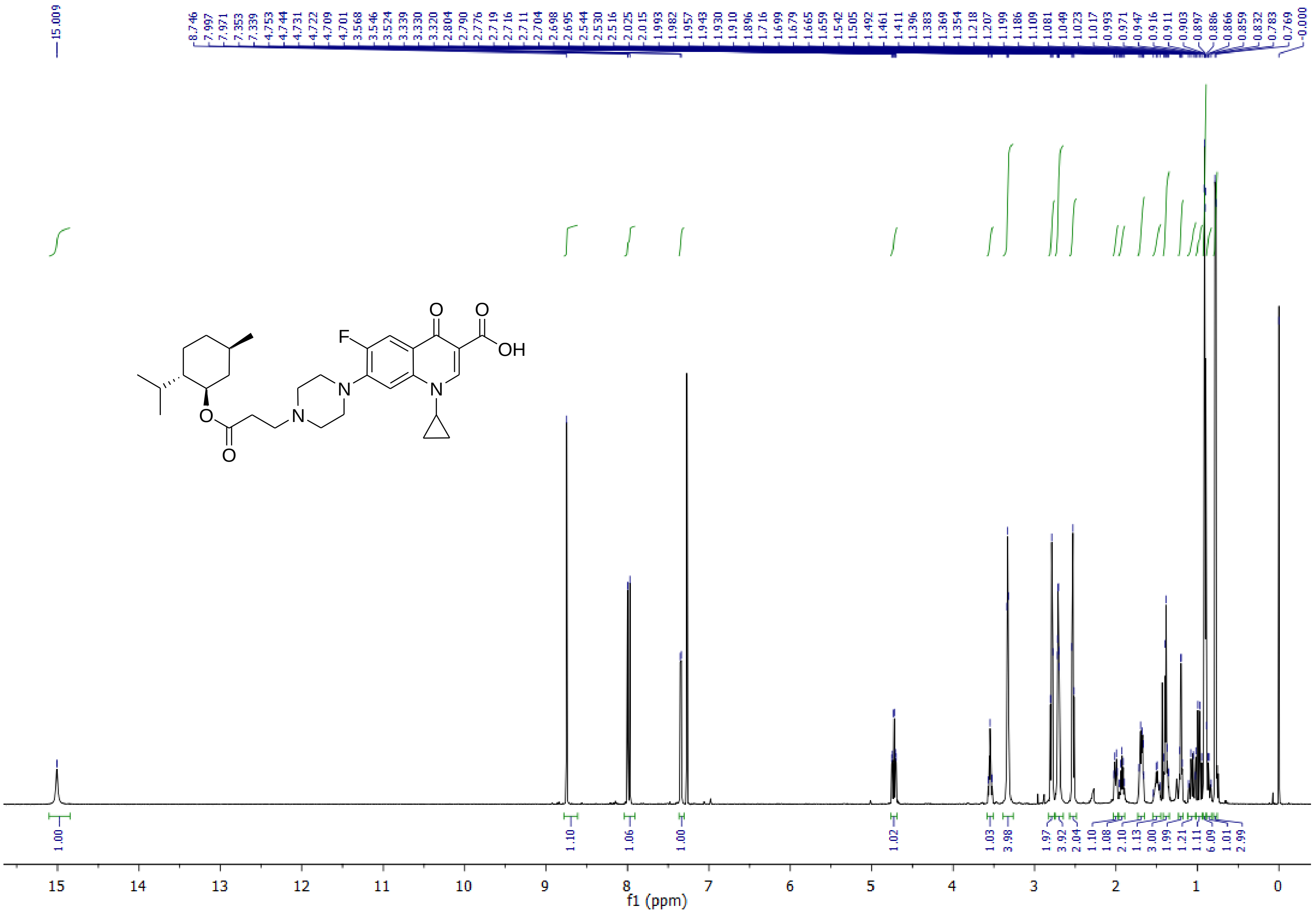

15.009
8.746 7.997 7.971 7.353 7.339 4.753 4.744 4.731 4.722 4.709 4.701 3.568 3.546 3.524 3.339 3.330 3.320 2.804 2.790 2.776 2.719 2.716 2.711 2.704 2.698 2.695 2.544 2.530 2.516 2.025 2.015 1.993 1.982 1.957 1.943 1.930 1.910 1.896 1.716 1.699 1.679 1.665 1.659 1.542 1.505 1.492 1.461 1.411 1.396 1.383 1.369 1.354 1.218 1.207 1.199 1.186 1.109 1.081 1.049 1.023 1.017 0.993 0.971 0.947 0.916 0.911 0.903 0.897 0.886 0.866 0.859 0.832 0.783 0.769 -0.000
F
O
O
OH
N
N
N
O
O
1.00
1.10
1.06
1.00
1.02
1.03
3.98
1.97
3.92
2.04
1.10
1.08
2.10
1.13
3.00
1.99
1.21
1.11
6.09
1.01
2.99
15 14 13 12 11 10 9 8 7 6 5 4 3 2 1 0
f1 (ppm)

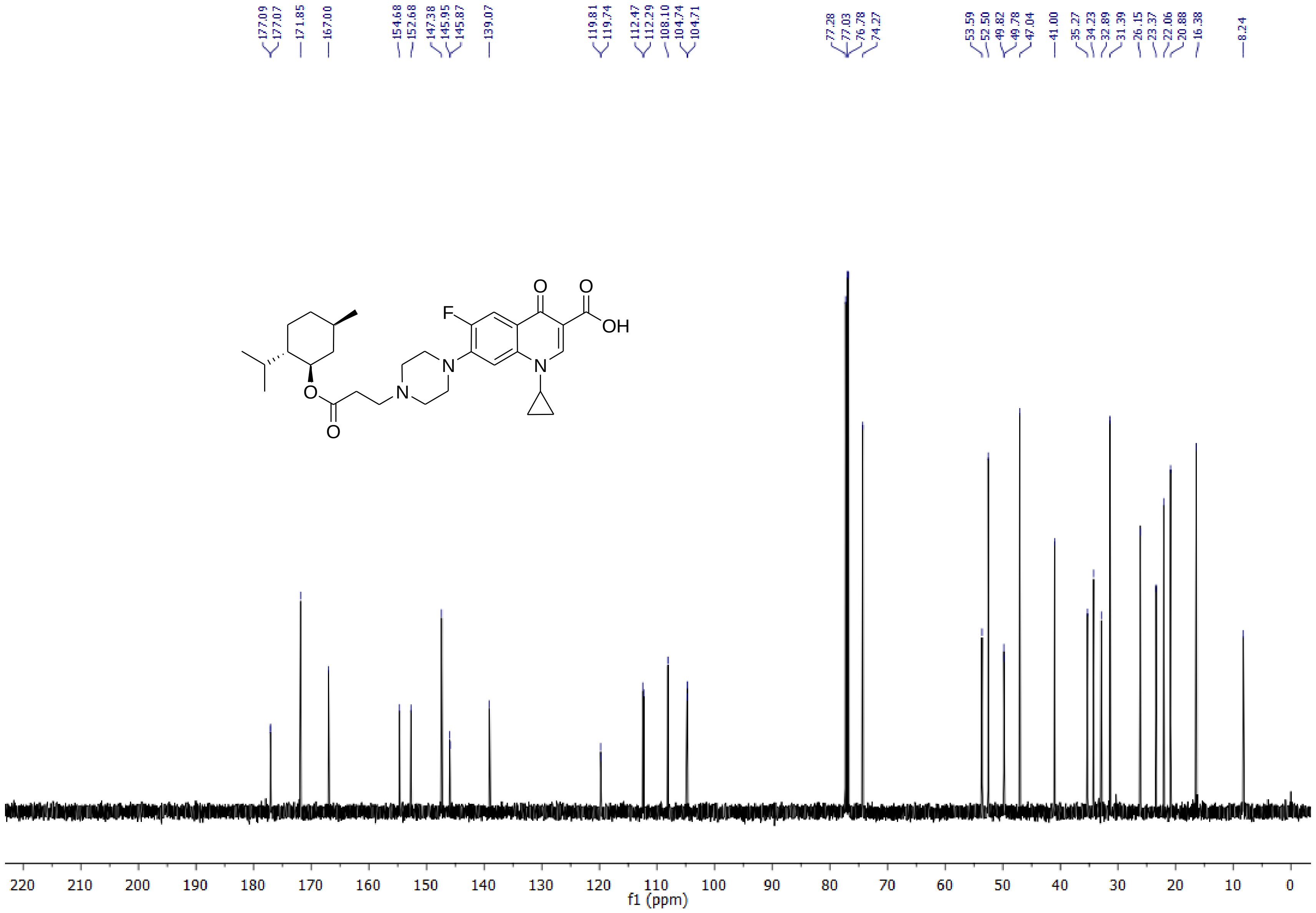
177.09
177.07
171.85
167.00
154.68
152.68
147.38
145.95
145.87
139.07
119.81
119.74
112.47
112.29
108.10
104.74
104.71
77.28
77.03
76.78
74.27
53.59
52.50
49.82
49.78
47.04
41.00
35.27
34.23
32.89
31.39
26.15
23.37
22.06
20.88
16.38
8.24
F
O
O
OH
N
N
N
O
O
220 210 200 190 180 170 160 150 140 130 120 110 100 90 80 70 60 50 40 30 20 10 0
f1 (ppm)

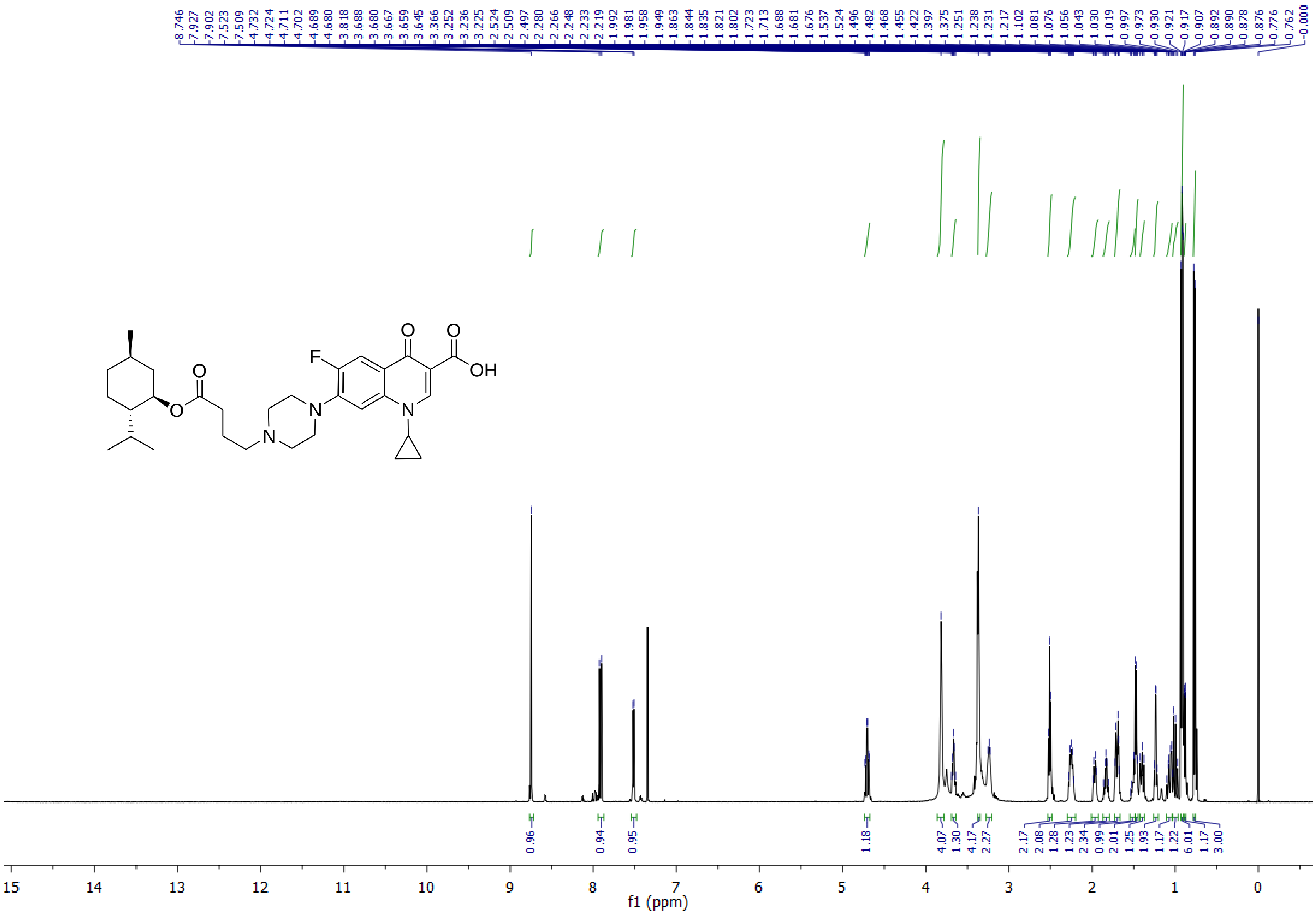

8.746 7.927 7.902 7.523 7.509 4.732 4.724 4.711 4.702 4.689 4.680 3.818 3.688 3.680 3.667 3.659 3.645 3.366 3.252 3.236 3.225 2.524 2.509 2.497 2.280 2.266 2.248 2.233 2.219 1.992 1.981 1.958 1.949 1.863 1.844 1.835 1.821 1.802 1.723 1.713 1.688 1.681 1.676 1.537 1.524 1.496 1.482 1.468 1.455 1.422 1.397 1.375 1.251 1.238 1.231 1.217 1.102 1.081 1.076 1.056 1.043 1.030 1.019 0.997 0.973 0.930 0.921 0.917 0.907 0.892 0.890 0.878 0.876 0.776 0.762 0.000
0.96 0.94 0.95 1.18 4.07 1.30 4.17 2.27 2.17 2.08 1.28 1.23 2.34 0.99 2.01 1.25 1.93 1.17 1.22 6.01 1.17 3.00
F
O
O
OH
O
O
N
N
N
15 14 13 12 11 10 9 8 7 6 5 4 3 2 1 0
f1 (ppm)

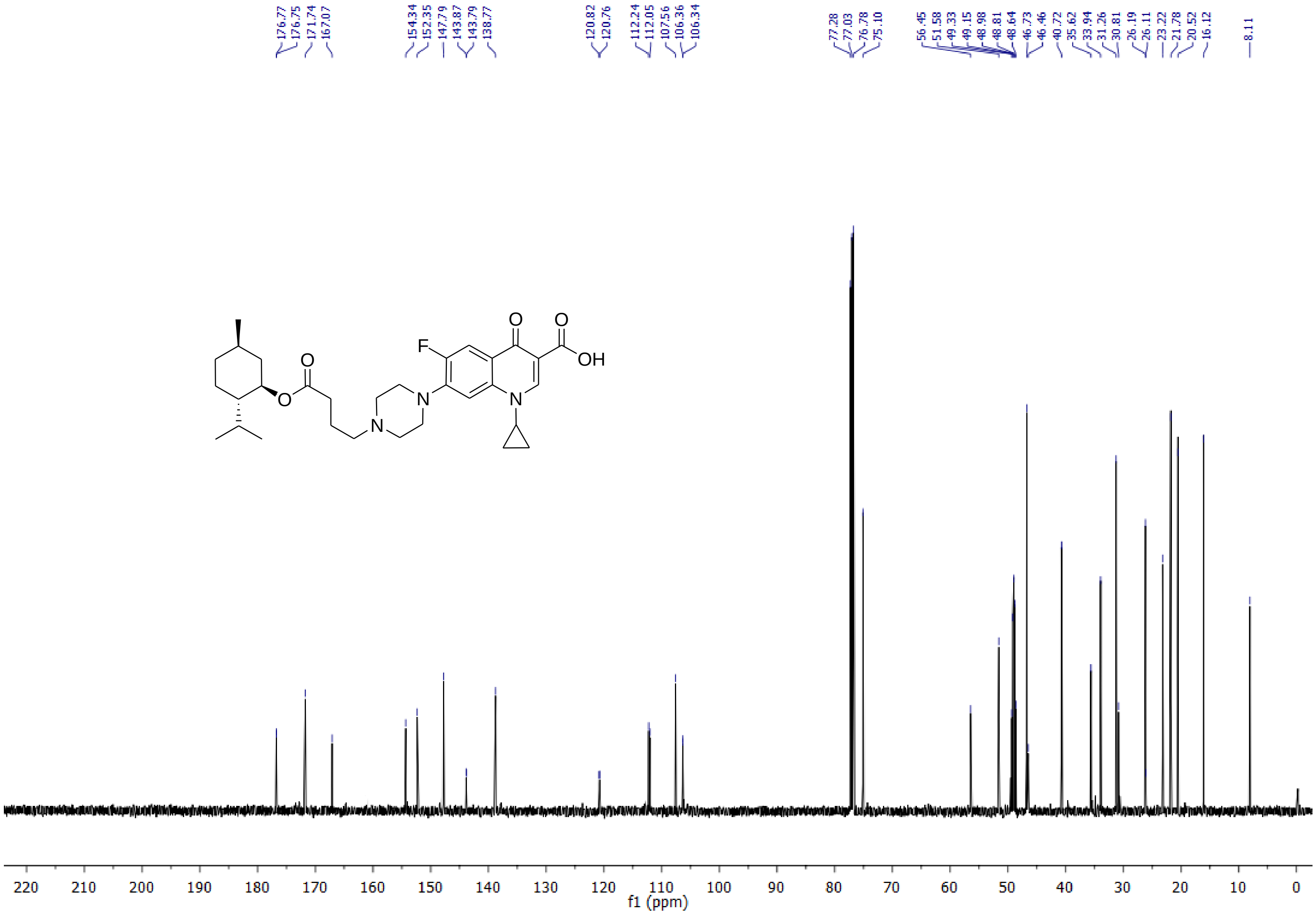
176.77
176.75
171.74
167.07
154.34
152.35
147.79
143.87
143.79
138.77
120.82
120.76
112.24
112.05
107.56
106.36
106.34
77.28
77.03
76.78
75.10
56.45
51.58
49.33
49.15
48.98
48.81
48.64
46.73
46.46
40.72
35.62
33.94
31.26
30.81
26.19
26.11
23.22
21.78
20.52
16.12
8.11
F
O
O
OH
O
O
N
N
N
220 210 200 190 180 170 160 150 140 130 120 110 100 90 80 70 60 50 40 30 20 10 0
f1 (ppm)

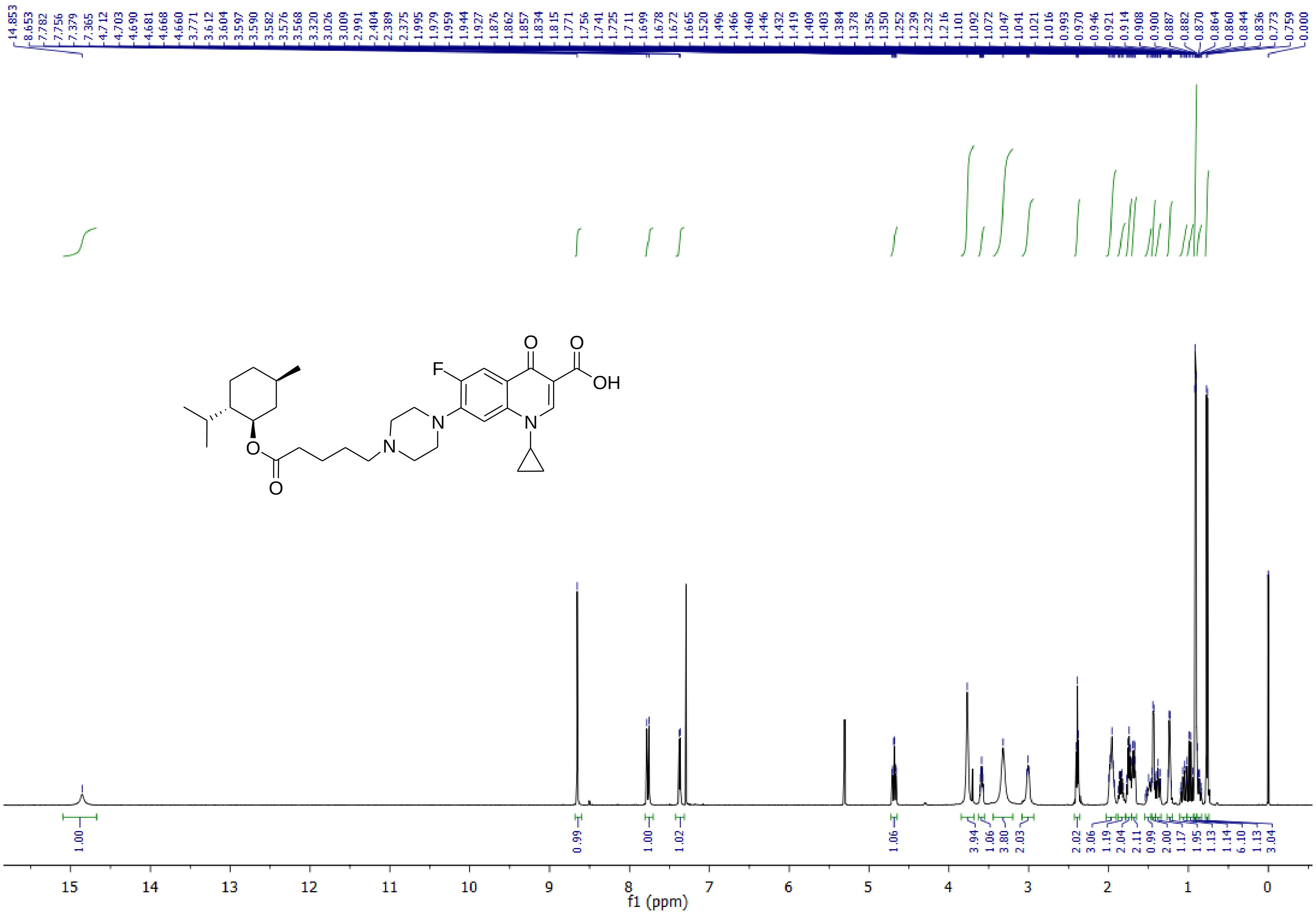
14.853
8.653
7.782
7.756
7.379
7.365
4.712
4.703
4.690
4.681
4.668
4.660
3.771
3.612
3.604
3.597
3.590
3.582
3.576
3.568
3.320
3.026
3.009
2.991
2.404
2.389
2.375
1.995
1.979
1.959
1.944
1.927
1.876
1.862
1.857
1.834
1.815
1.771
1.756
1.741
1.725
1.711
1.699
1.678
1.672
1.665
1.520
1.496
1.466
1.460
1.446
1.432
1.419
1.409
1.403
1.384
1.378
1.356
1.350
1.252
1.239
1.232
1.216
1.101
1.092
1.072
1.047
1.041
1.021
1.016
0.993
0.970
0.946
0.921
0.914
0.908
0.900
0.887
0.882
0.870
0.864
0.860
0.844
0.836
0.773
0.759
0.000
F
O
O
OH
N
N
N
O
O
1.00
0.99
1.00
1.02
1.06
3.94
1.06
3.80
2.03
2.02
3.06
1.19
2.04
2.11
0.99
2.00
1.17
1.95
1.13
1.14
6.10
1.13
3.04
15
14
13
12
11
10
9
8
7
6
5
4
3
2
1
0
f1 (ppm)

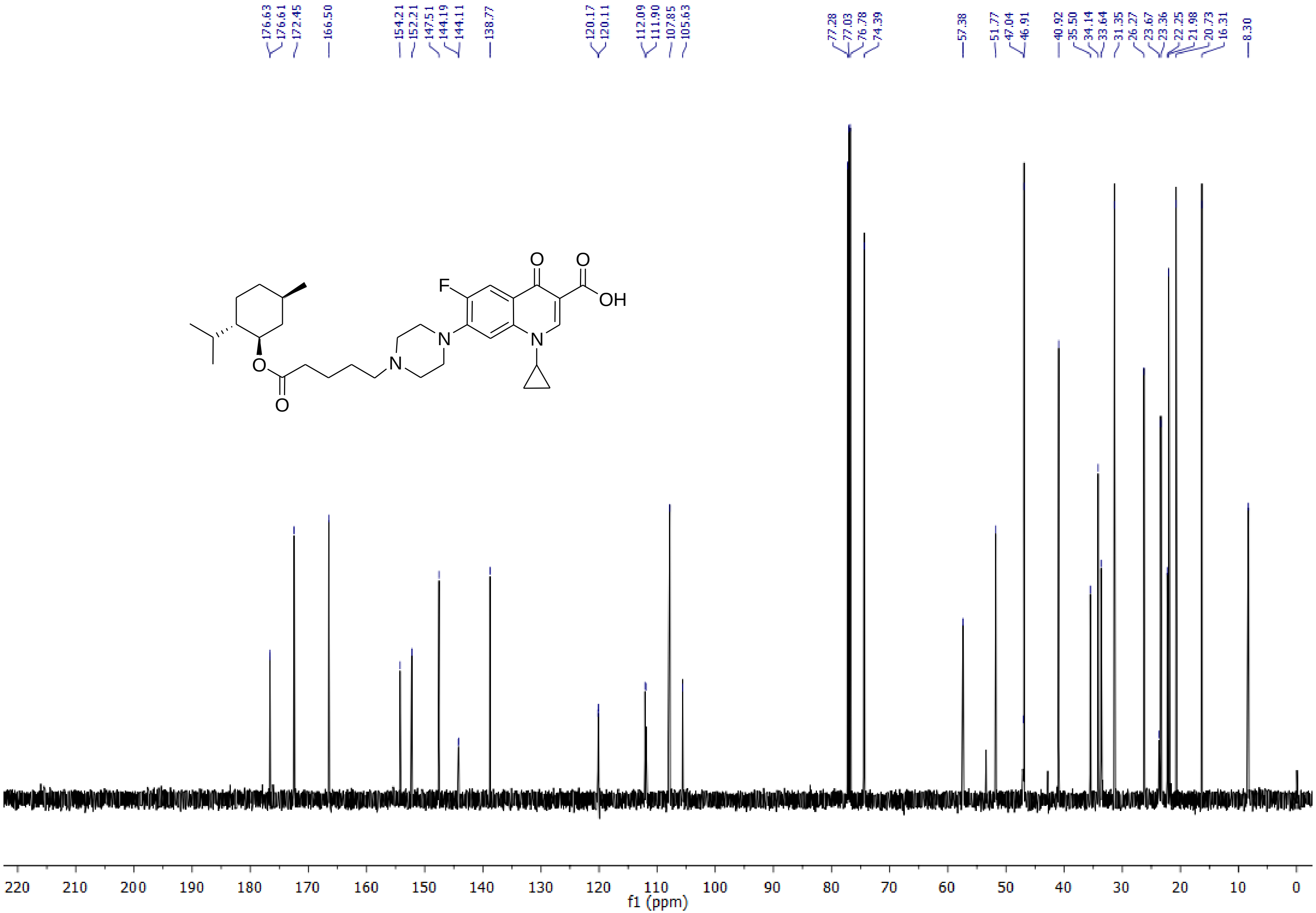

176.63
176.61
172.45
166.50
154.21
152.21
147.51
144.19
144.11
138.77
120.17
120.11
112.09
111.90
107.85
105.63
77.28
77.03
76.78
74.39
57.38
51.77
47.04
46.91
40.92
35.50
34.14
33.64
31.35
26.27
23.67
23.36
22.25
21.98
20.73
16.31
8.30
F
O
O
OH
N
N
N
O
O
220 210 200 190 180 170 160 150 140 130 120 110 100 90 80 70 60 50 40 30 20 10 0
f1 (ppm)

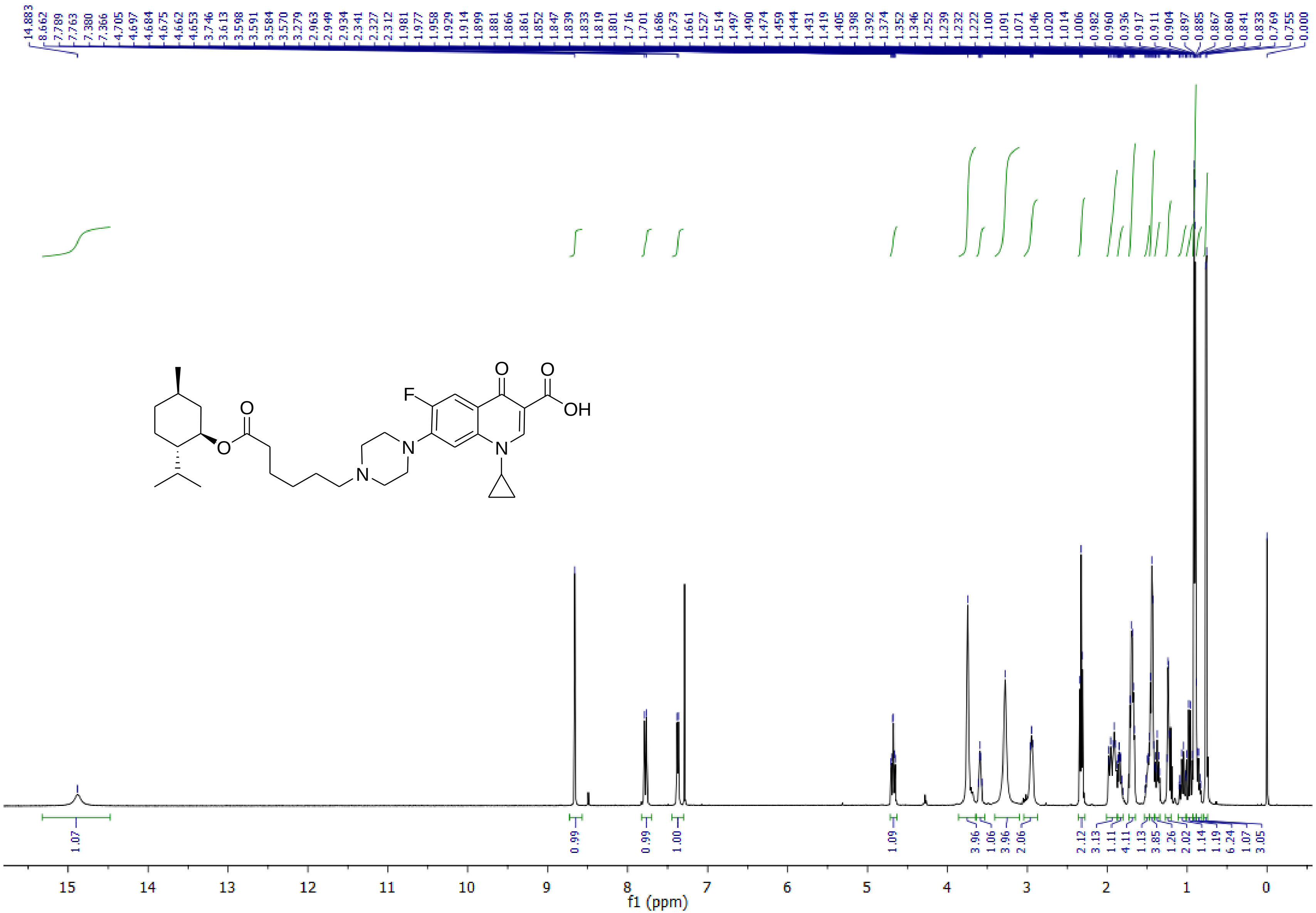
14.883 8.662 7.789 7.763 7.380 7.366 4.705 4.697 4.684 4.675 4.662 4.653 3.746 3.613 3.598 3.591 3.584 3.570 3.279 2.963 2.949 2.934 2.341 2.327 2.312 1.981 1.977 1.958 1.929 1.914 1.899 1.881 1.866 1.861 1.852 1.847 1.839 1.833 1.819 1.801 1.716 1.701 1.686 1.673 1.661 1.527 1.514 1.497 1.490 1.474 1.459 1.444 1.431 1.419 1.405 1.398 1.392 1.374 1.352 1.346 1.252 1.239 1.232 1.222 1.100 1.091 1.071 1.046 1.020 1.014 1.006 0.982 0.960 0.936 0.917 0.911 0.904 0.897 0.885 0.867 0.860 0.841 0.833 0.769 0.755 0.000
F
O
O
OH
N
N
N
O
O
1.07 0.99 0.99 1.00 1.09 3.96 1.06 3.96 2.06 2.12 3.13 1.11 4.11 1.13 3.85 1.26 2.02 1.14 1.19 6.24 1.07 3.05
15 14 13 12 11 10 9 8 7 6 5 4 3 2 1 0
f1 (ppm)

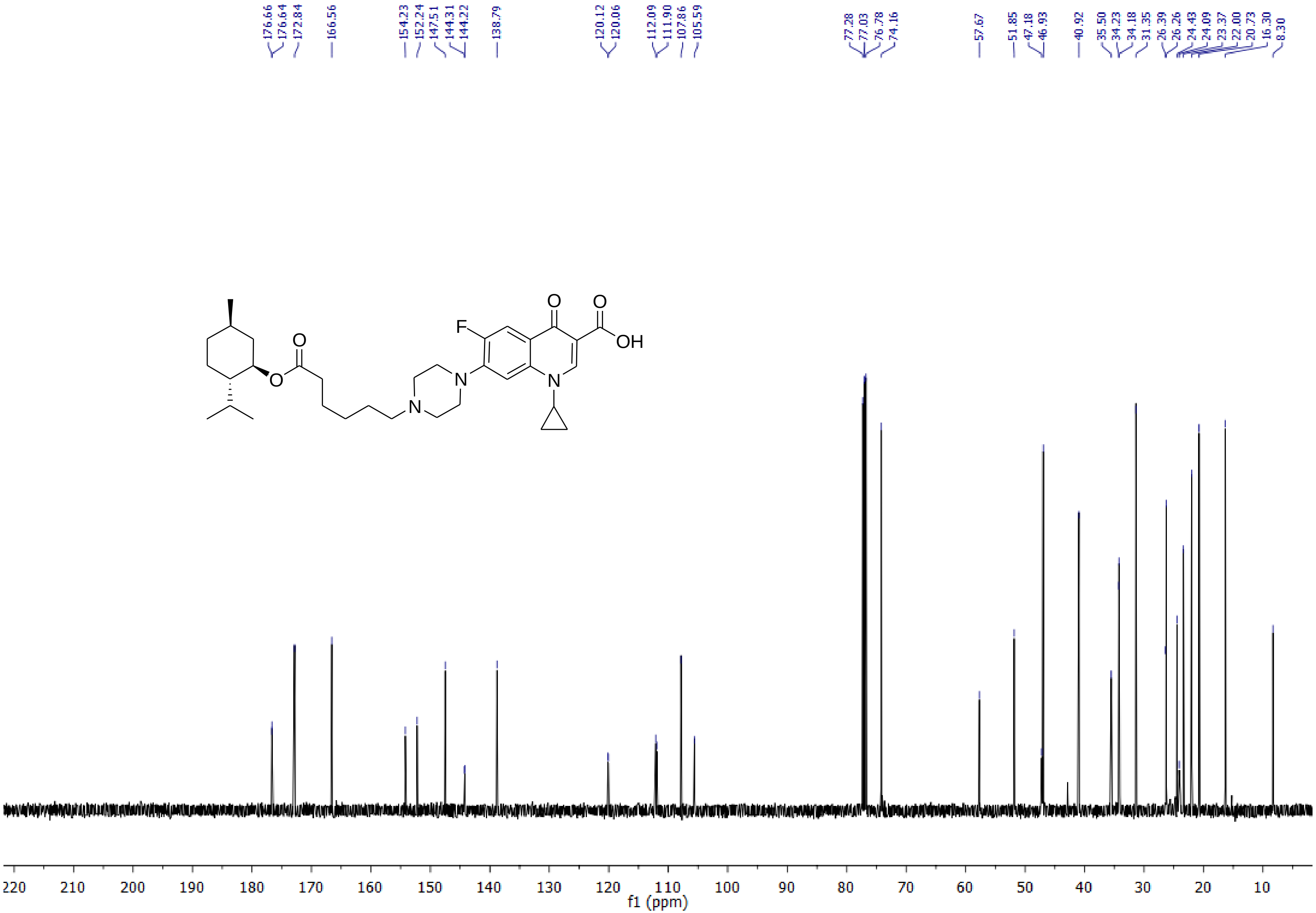
176.66
176.64
172.84
166.56
154.23
152.24
147.51
144.31
144.22
138.79
120.12
120.06
112.09
111.90
107.86
105.59
77.28
77.03
76.78
74.16
57.67
51.85
47.18
46.93
40.92
35.50
34.23
34.18
31.35
26.39
26.26
24.43
24.09
23.37
22.00
20.73
16.30
8.30
F
O
O
OH
O
O
N
N
N
220
210
200
190
180
170
160
150
140
130
120
110
100
90
80
70
60
50
40
30
20
10
f1 (ppm)

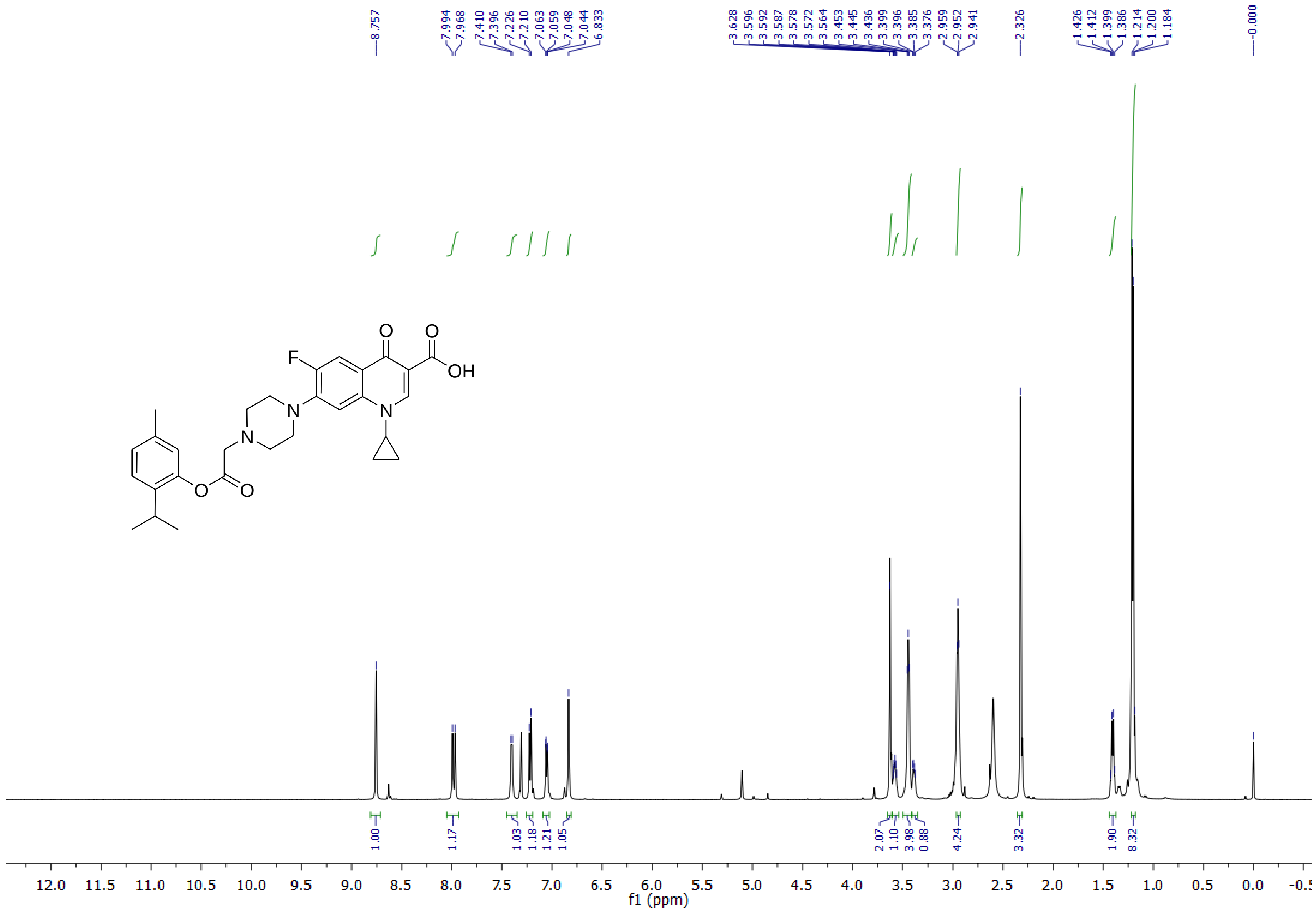
8.757
7.994
7.968
7.410
7.396
7.226
7.210
7.063
7.059
7.048
7.044
6.833
3.628
3.596
3.592
3.587
3.578
3.572
3.564
3.453
3.445
3.436
3.399
3.396
3.385
3.376
2.959
2.952
2.941
2.326
1.426
1.412
1.399
1.386
1.214
1.200
1.184
-0.000
F
O
O
OH
N
N
N
O
O
1.00
1.17
1.03
1.18
1.21
1.05
2.07
1.10
3.98
0.88
4.24
3.32
1.90
8.32
12.0
11.5
11.0
10.5
10.0
9.5
9.0
8.5
8.0
7.5
7.0
6.5
6.0
5.5
5.0
4.5
4.0
3.5
3.0
2.5
2.0
1.5
1.0
0.5
0.0
f1 (ppm)

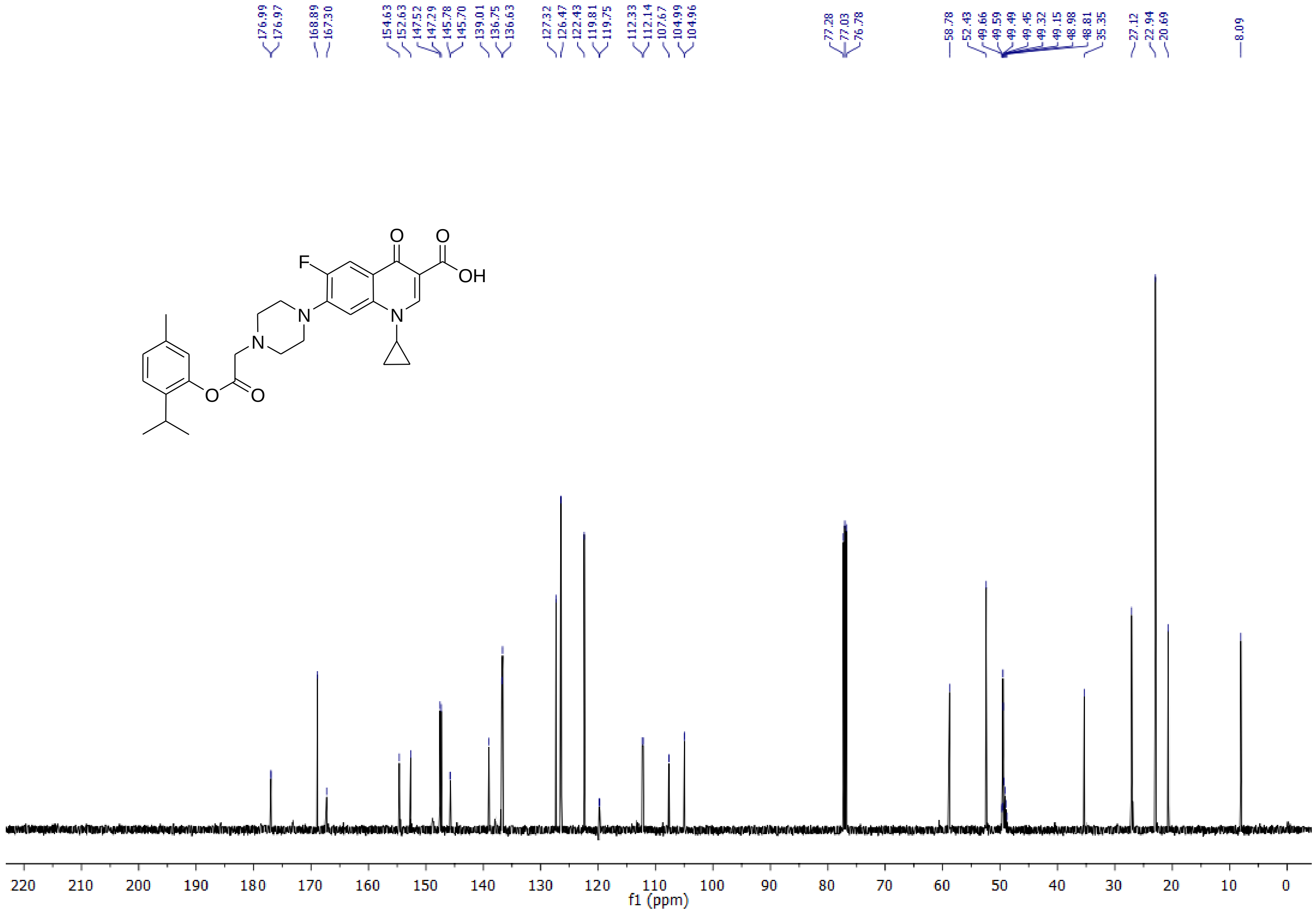

176.99
176.97
168.89
167.30
154.63
152.63
147.52
147.29
145.78
145.70
139.01
136.75
136.63
127.32
126.47
122.43
119.81
119.75
112.33
112.14
107.67
104.99
104.96
77.28
77.03
76.78
58.78
52.43
49.66
49.59
49.49
49.45
49.32
49.15
48.98
48.81
35.35
27.12
22.94
20.69
8.09
F
O
O
OH
N
N
N
O
O
220 210 200 190 180 170 160 150 140 130 120 110 100 90 80 70 60 50 40 30 20 10 0
f1 (ppm)

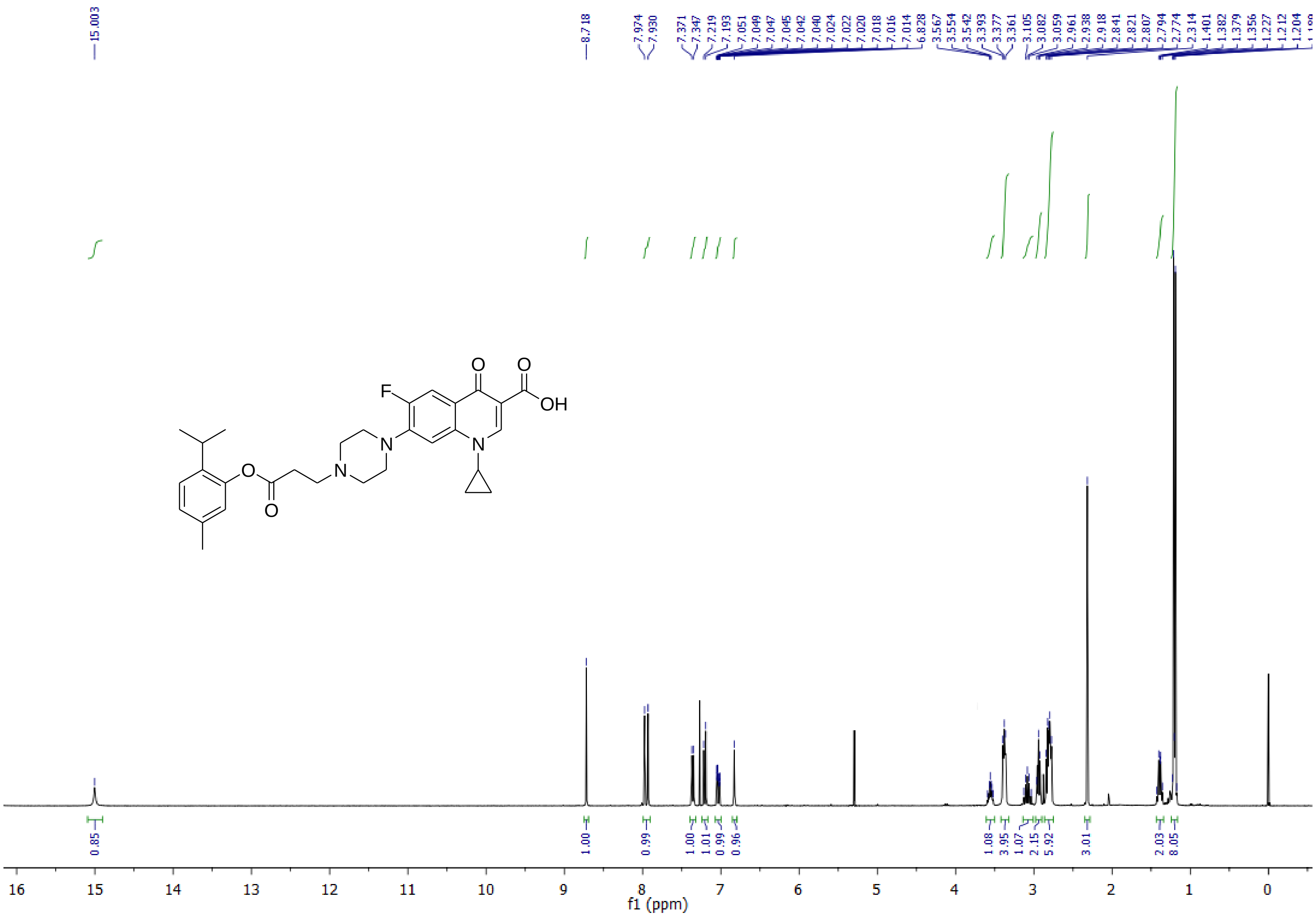
15.003
8.718
7.974
7.930
7.371
7.347
7.219
7.193
7.051
7.049
7.047
7.045
7.042
7.040
7.024
7.022
7.020
7.018
7.016
7.014
6.828
3.567
3.554
3.542
3.393
3.377
3.361
3.105
3.082
3.059
2.961
2.938
2.918
2.841
2.821
2.807
2.794
2.774
2.314
1.401
1.382
1.379
1.356
1.227
1.212
1.204
F
O
O
OH
N
N
N
O
O
0.85
1.00
0.99
1.00
1.01
0.99
0.96
1.08
3.95
1.07
2.15
5.92
3.01
2.03
8.05
16
15
14
13
12
11
10
9
8
7
6
5
4
3
2
1
0
f1 (ppm)

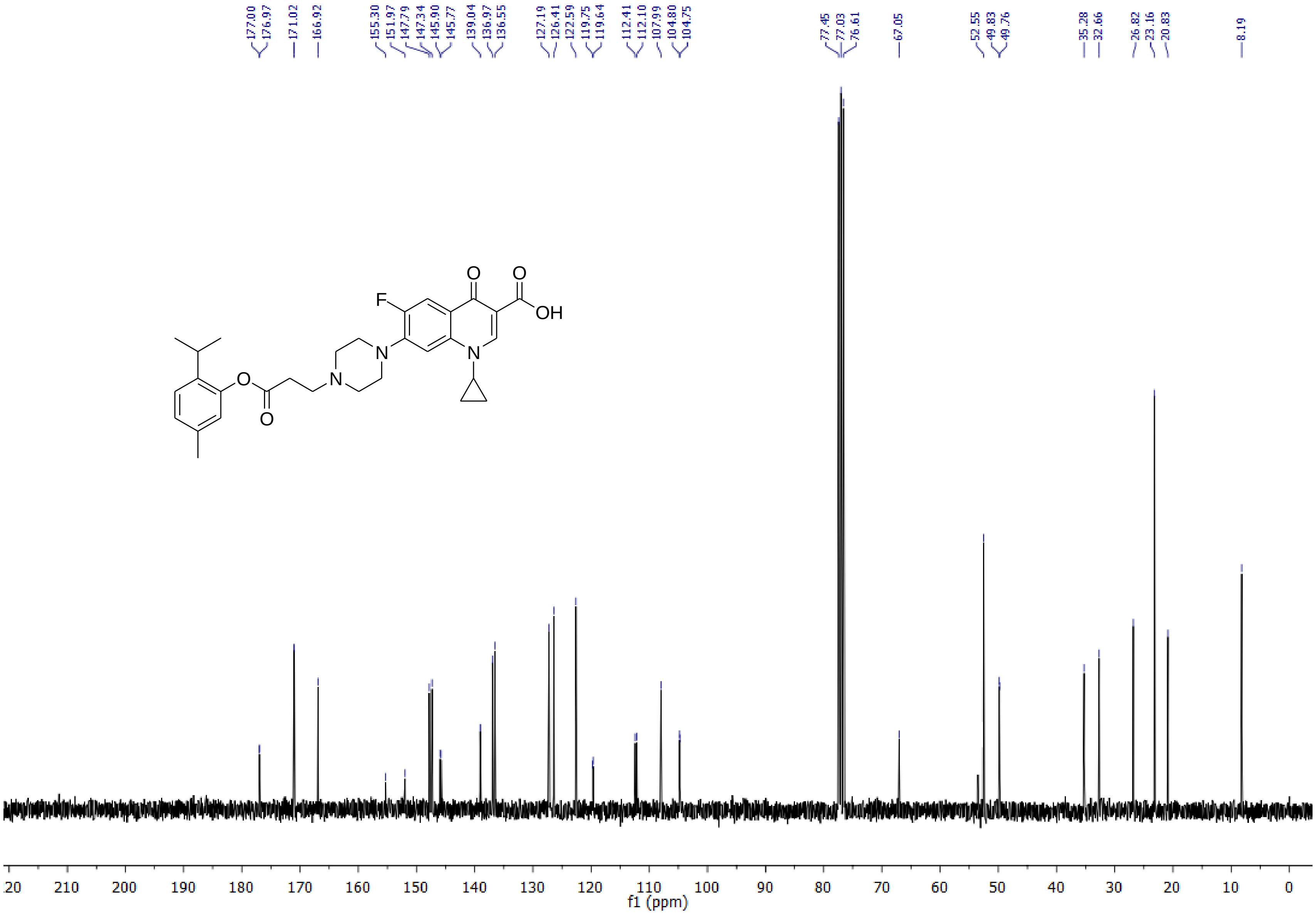
177.00
176.97
171.02
166.92
155.30
151.97
147.79
147.34
145.90
145.77
139.04
136.97
136.55
127.19
126.41
122.59
119.75
119.64
112.41
112.10
107.99
104.80
104.75
77.45
77.03
76.61
67.05
52.55
49.83
49.76
35.28
32.66
26.82
23.16
20.83
8.19
F
O
O
OH
N
N
N
O
O
20
210
200
190
180
170
160
150
140
130
120
110
100
90
80
70
60
50
40
30
20
10
0
f1 (ppm)

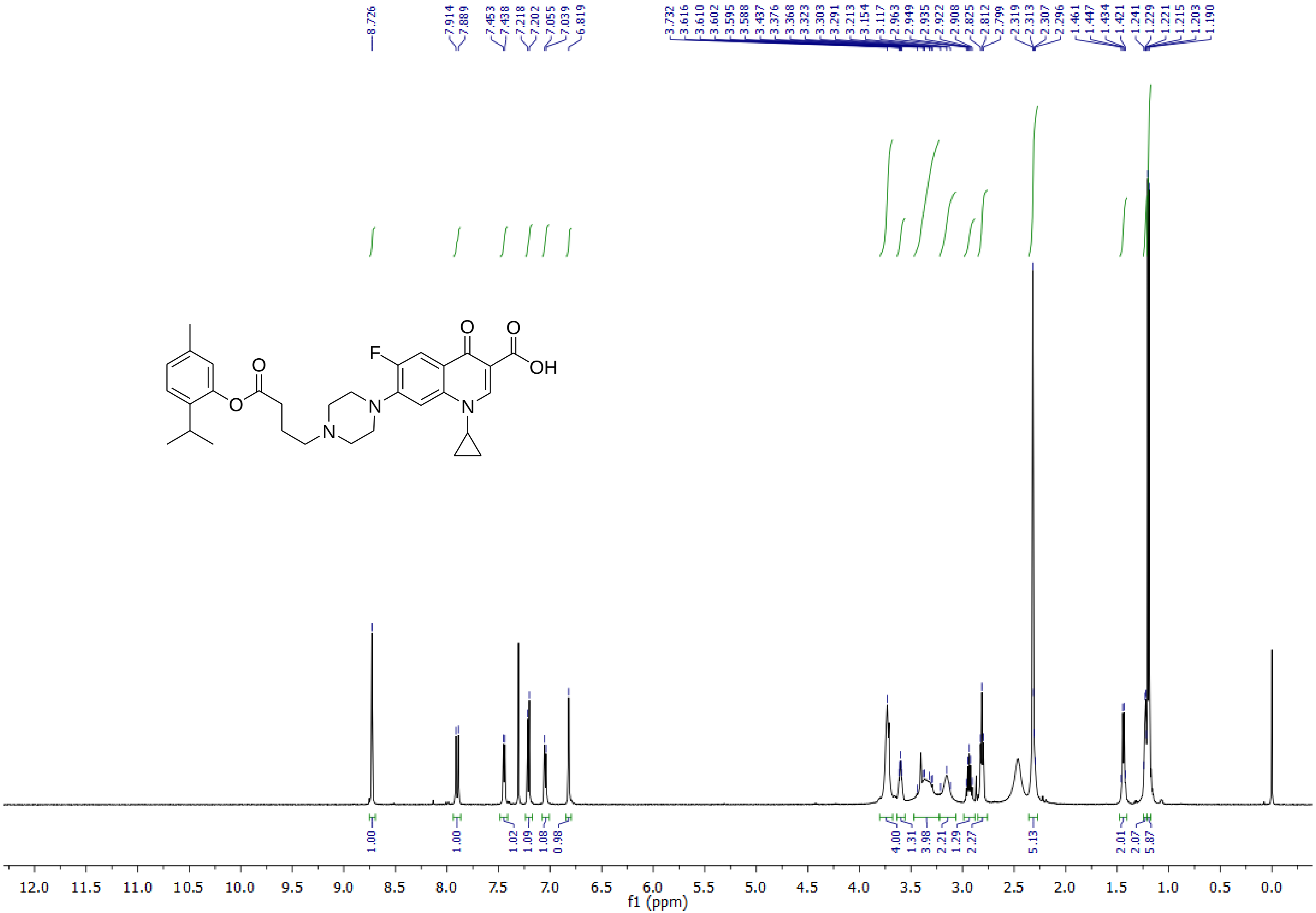
8.726
7.914
7.889
7.453
7.438
7.218
7.202
7.055
7.039
6.819
3.732
3.616
3.610
3.602
3.595
3.588
3.437
3.376
3.368
3.323
3.303
3.291
3.213
3.154
3.117
2.963
2.949
2.935
2.922
2.908
2.825
2.812
2.799
2.319
2.313
2.307
2.296
1.461
1.447
1.434
1.421
1.241
1.229
1.221
1.215
1.203
1.190
1.00
1.00
1.02
1.09
1.08
0.98
4.00
1.31
3.98
2.21
1.29
2.27
5.13
2.01
2.07
5.87
12.0 11.5 11.0 10.5 10.0 9.5 9.0 8.5 8.0 7.5 7.0 6.5 6.0 5.5 5.0 4.5 4.0 3.5 3.0 2.5 2.0 1.5 1.0 0.5 0.0
f1 (ppm)
F
O
O
OH
O
O
N
N
N

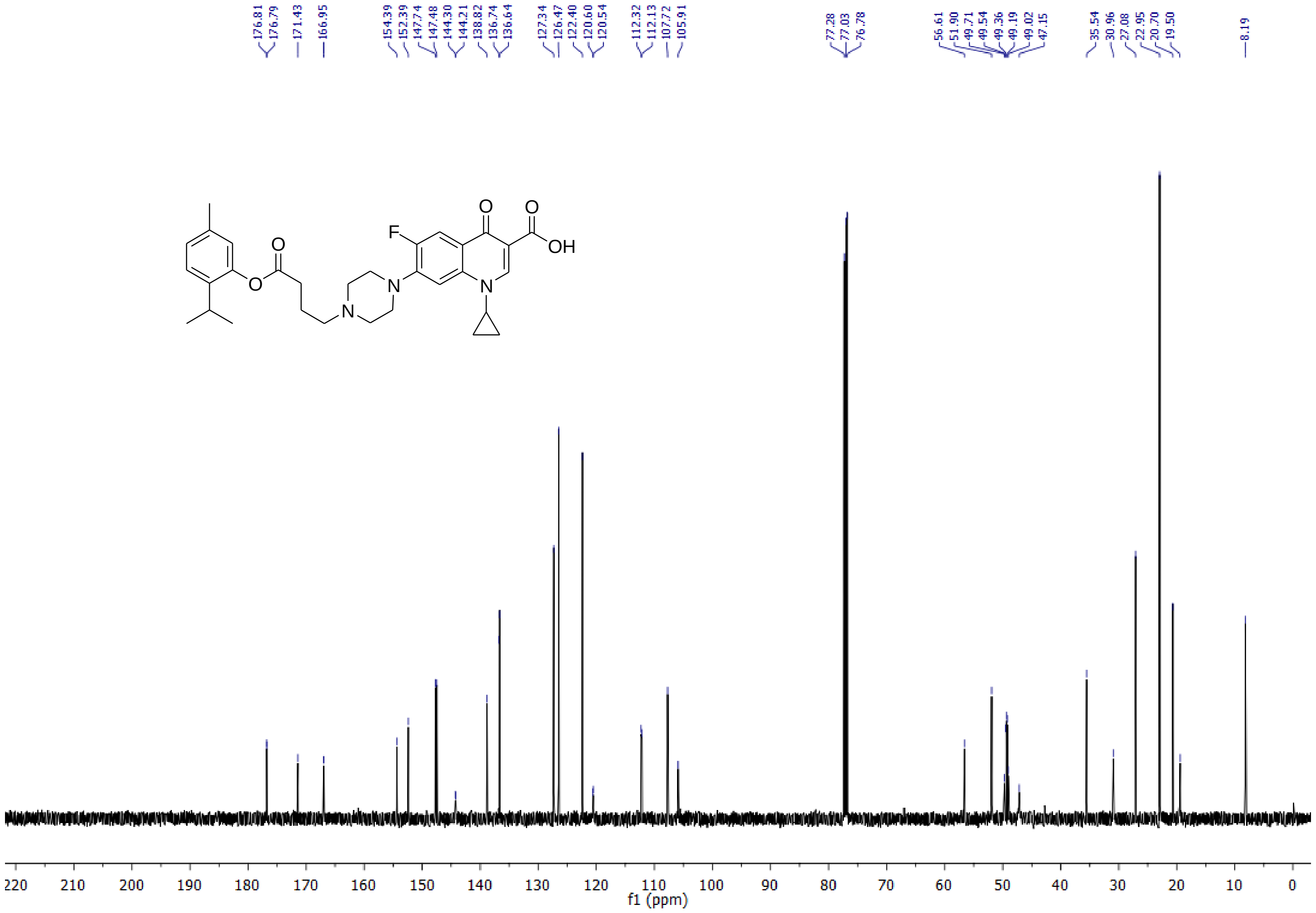

176.81
176.79
171.43
166.95
154.39
152.39
147.74
147.48
144.30
144.21
138.82
136.74
136.64
127.34
126.47
122.40
120.60
120.54
112.32
112.13
107.72
105.91
77.28
77.03
76.78
56.61
51.90
49.71
49.54
49.36
49.19
49.02
47.15
35.54
30.96
27.08
22.95
20.70
19.50
8.19
F
O
O
OH
O
O
N
N
N
220
210
200
190
180
170
160
150
140
130
120
110
100
90
80
70
60
50
40
30
20
10
0
f1 (ppm)

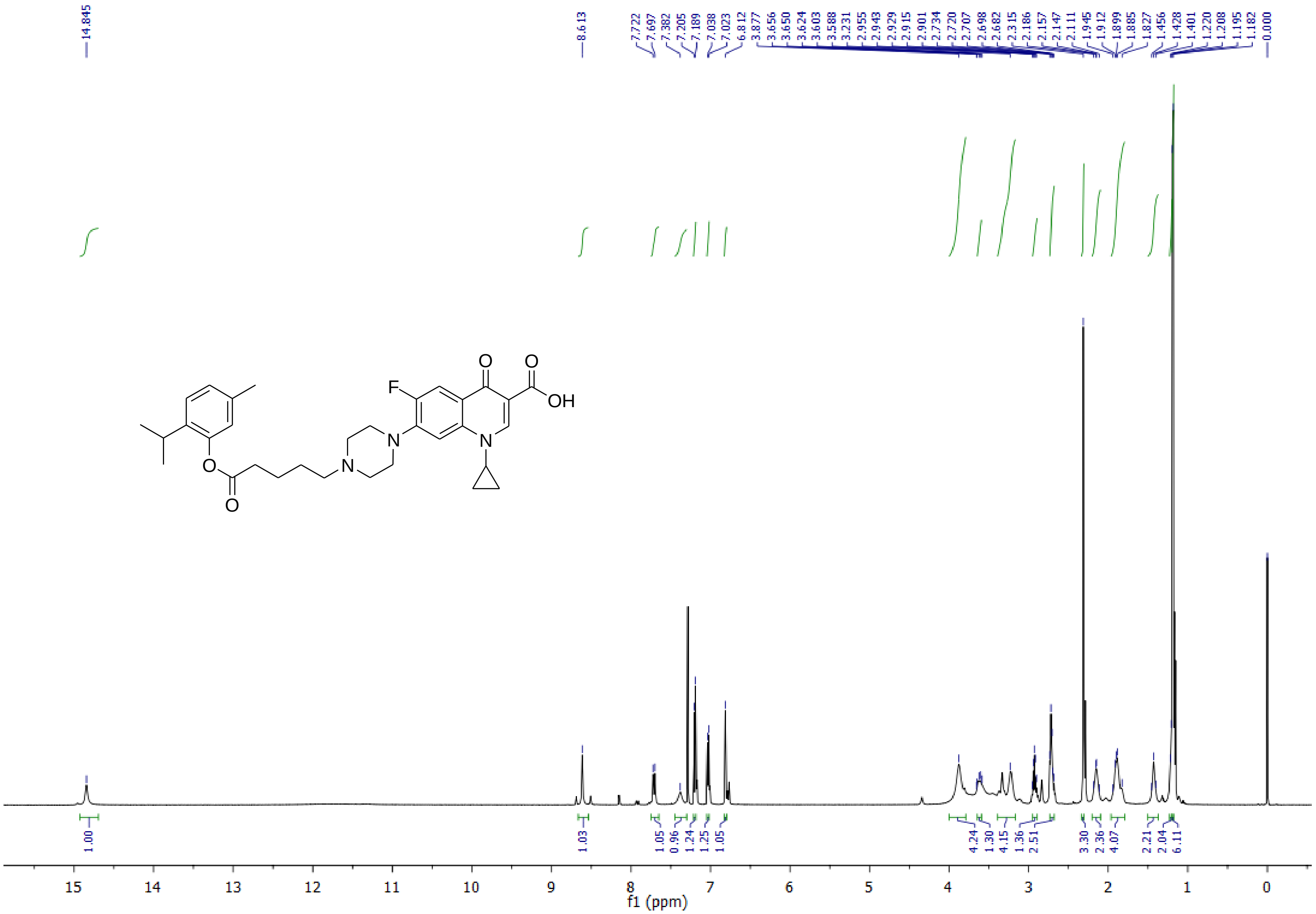

14.845
8.613
7.722
7.697
7.382
7.205
7.189
7.038
7.023
6.812
3.877
3.656
3.650
3.624
3.603
3.588
3.231
2.955
2.943
2.929
2.915
2.901
2.734
2.720
2.707
2.698
2.682
2.315
2.186
2.157
2.147
2.111
1.945
1.912
1.899
1.885
1.827
1.456
1.428
1.401
1.220
1.208
1.195
1.182
0.000
F
O
O
OH
N
N
N
O
O
1.00
1.03
1.05
0.96
1.24
1.25
1.05
4.24
1.30
4.15
1.36
2.51
3.30
2.36
4.07
2.21
2.04
6.11
15
14
13
12
11
10
9
8
7
6
5
4
3
2
1
0
f1 (ppm)

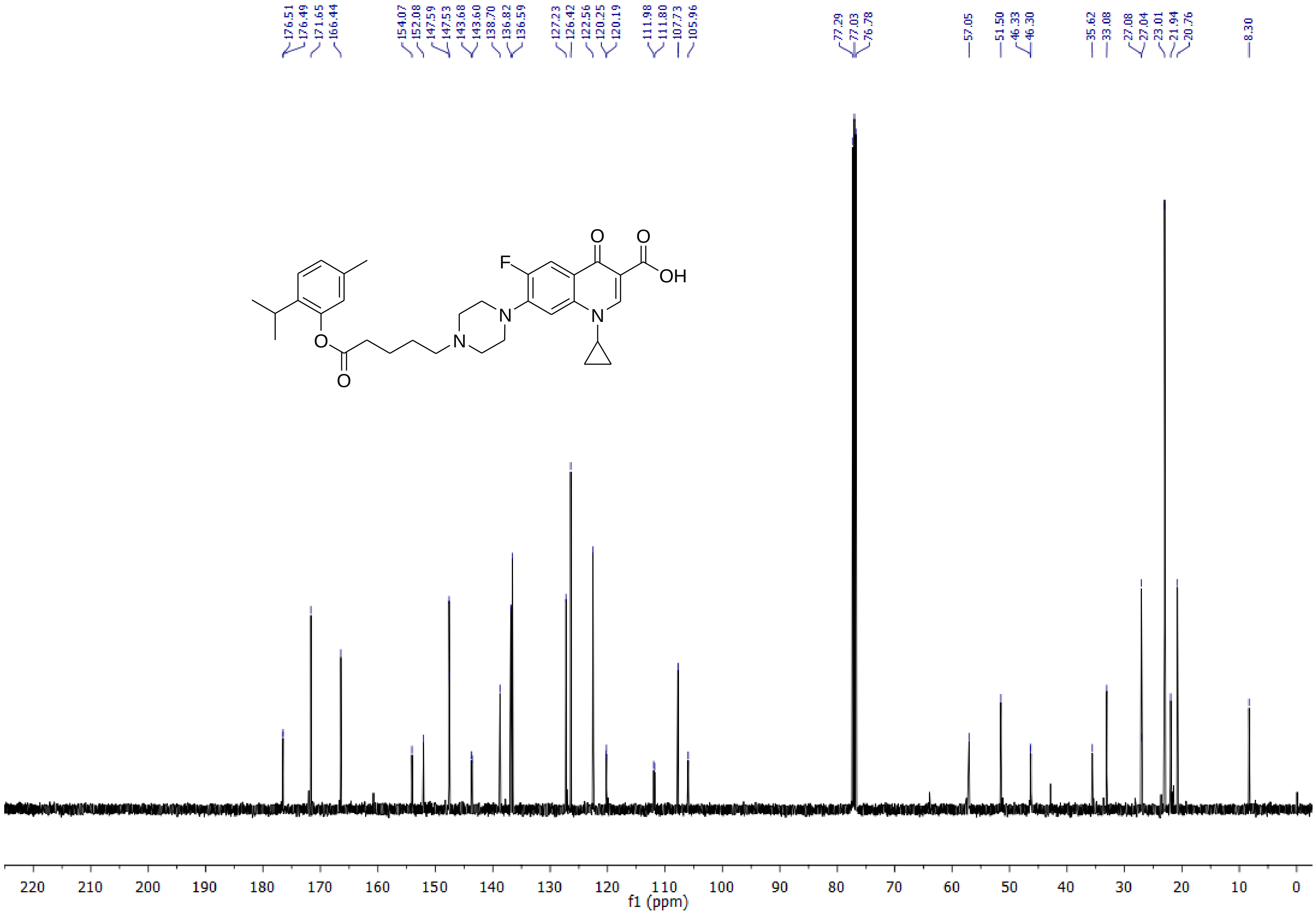
176.51
176.49
171.65
166.44
154.07
152.08
147.59
147.53
143.68
143.60
138.70
136.82
136.59
127.23
126.42
122.56
120.25
120.19
111.98
111.80
107.73
105.96
77.29
77.03
76.78
57.05
51.50
46.33
46.30
35.62
33.08
27.08
27.04
23.01
21.94
20.76
8.30
F
O
O
OH
N
N
N
O
O
220 210 200 190 180 170 160 150 140 130 120 110 100 90 80 70 60 50 40 30 20 10 0
f1 (ppm)

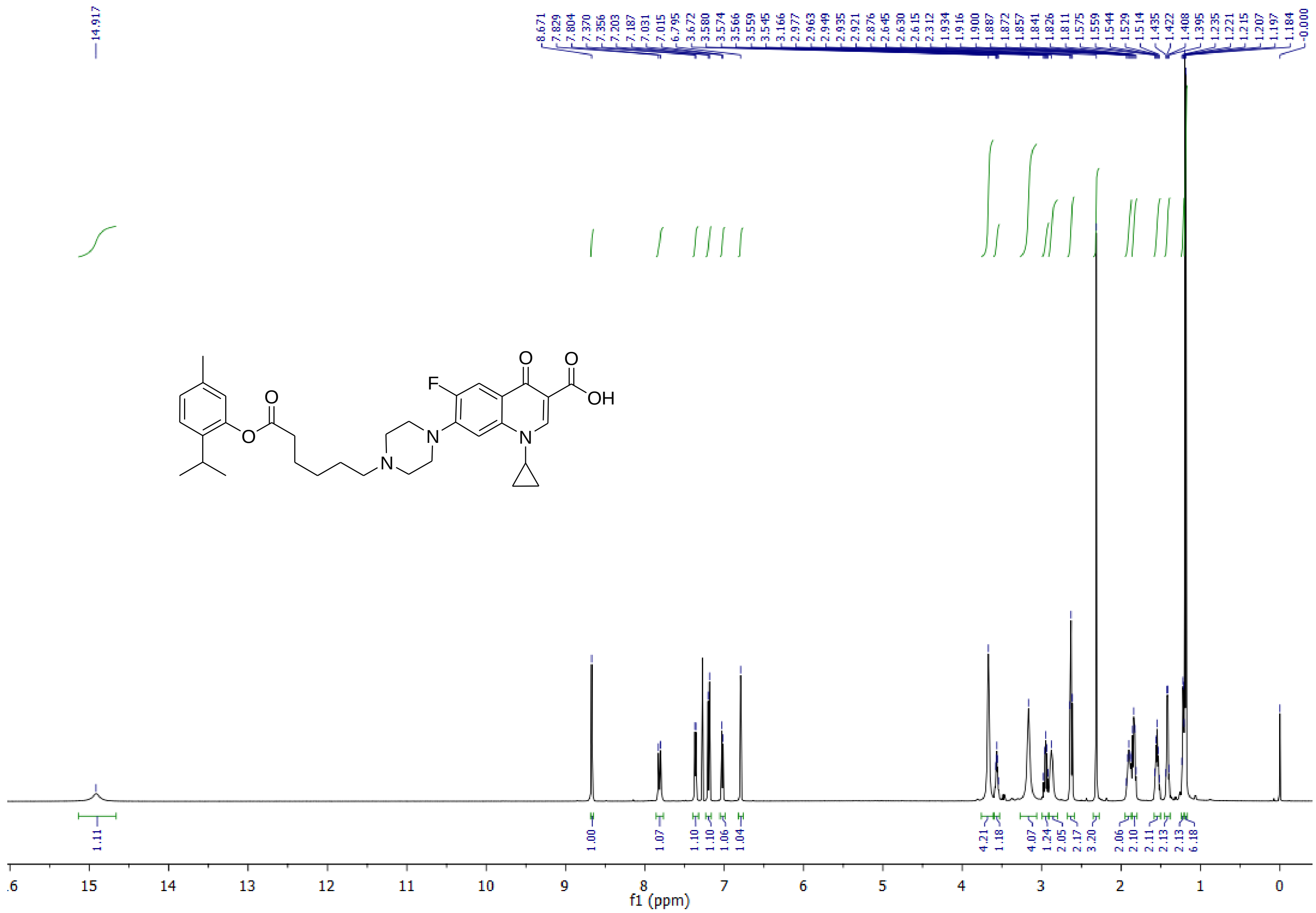

14.917
8.671
7.829
7.804
7.370
7.356
7.203
7.187
7.031
7.015
6.795
3.672
3.580
3.574
3.566
3.559
3.545
3.166
2.977
2.963
2.949
2.935
2.921
2.876
2.645
2.630
2.615
2.312
1.934
1.916
1.900
1.887
1.872
1.857
1.841
1.826
1.811
1.575
1.559
1.544
1.529
1.514
1.435
1.422
1.408
1.395
1.235
1.221
1.215
1.207
1.197
1.184
-0.000
F
O
O
OH
O
O
N
N
N
1.11
1.00
1.07
1.10
1.10
1.06
1.04
4.21
1.18
4.07
1.24
2.05
2.17
3.20
2.06
2.10
2.11
2.13
2.13
6.18
f1 (ppm)

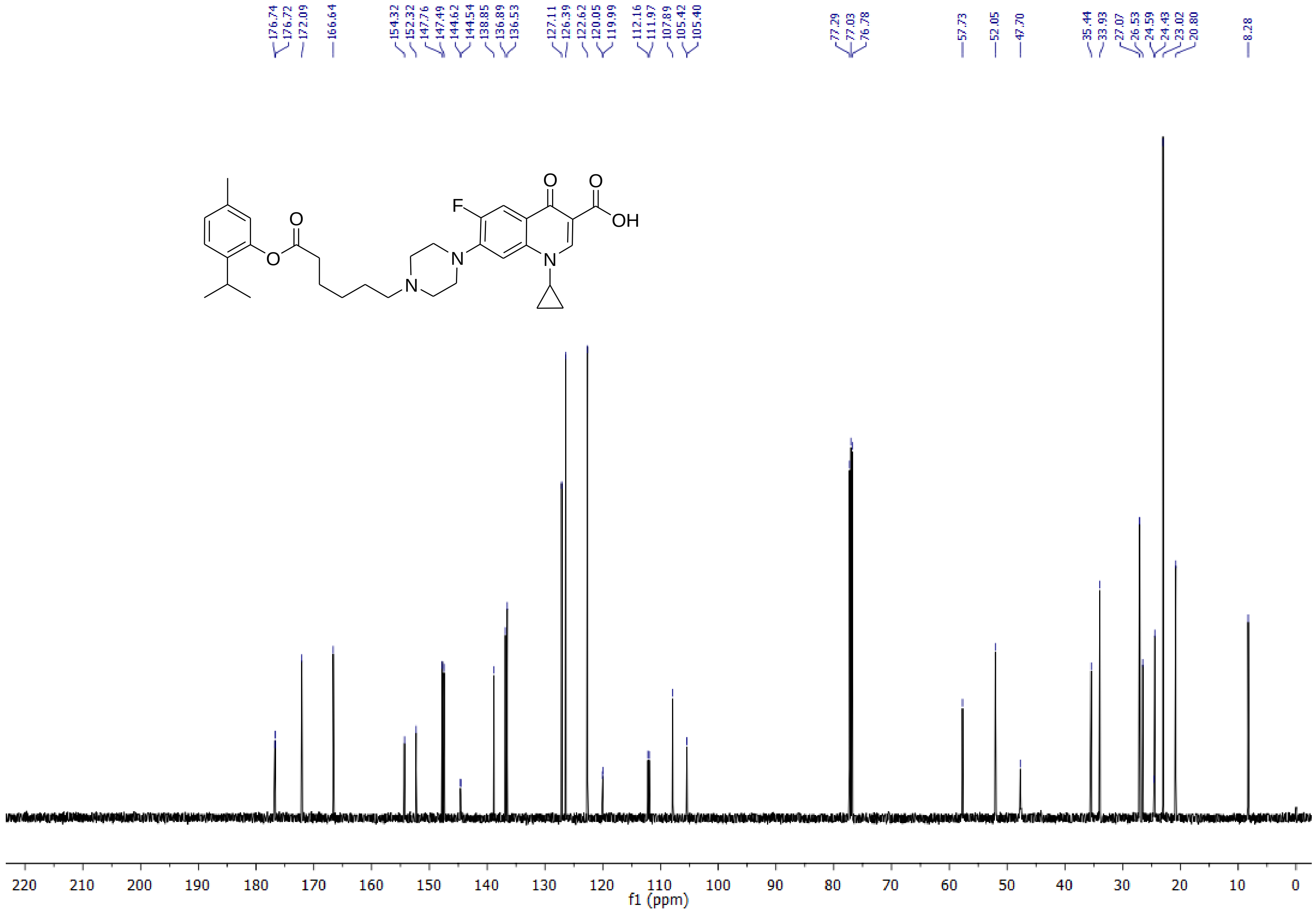
176.74
176.72
172.09
166.64
154.32
152.32
147.76
147.49
144.62
144.54
138.85
136.89
136.53
127.11
126.39
122.62
120.05
119.99
112.16
111.97
107.89
105.42
105.40
77.29
77.03
76.78
57.73
52.05
47.70
35.44
33.93
27.07
26.53
24.59
24.43
23.02
20.80
8.28
F
O
O
OH
O
O
N
N
N
220 210 200 190 180 170 160 150 140 130 120 110 100 90 80 70 60 50 40 30 20 10 0
f1 (ppm)

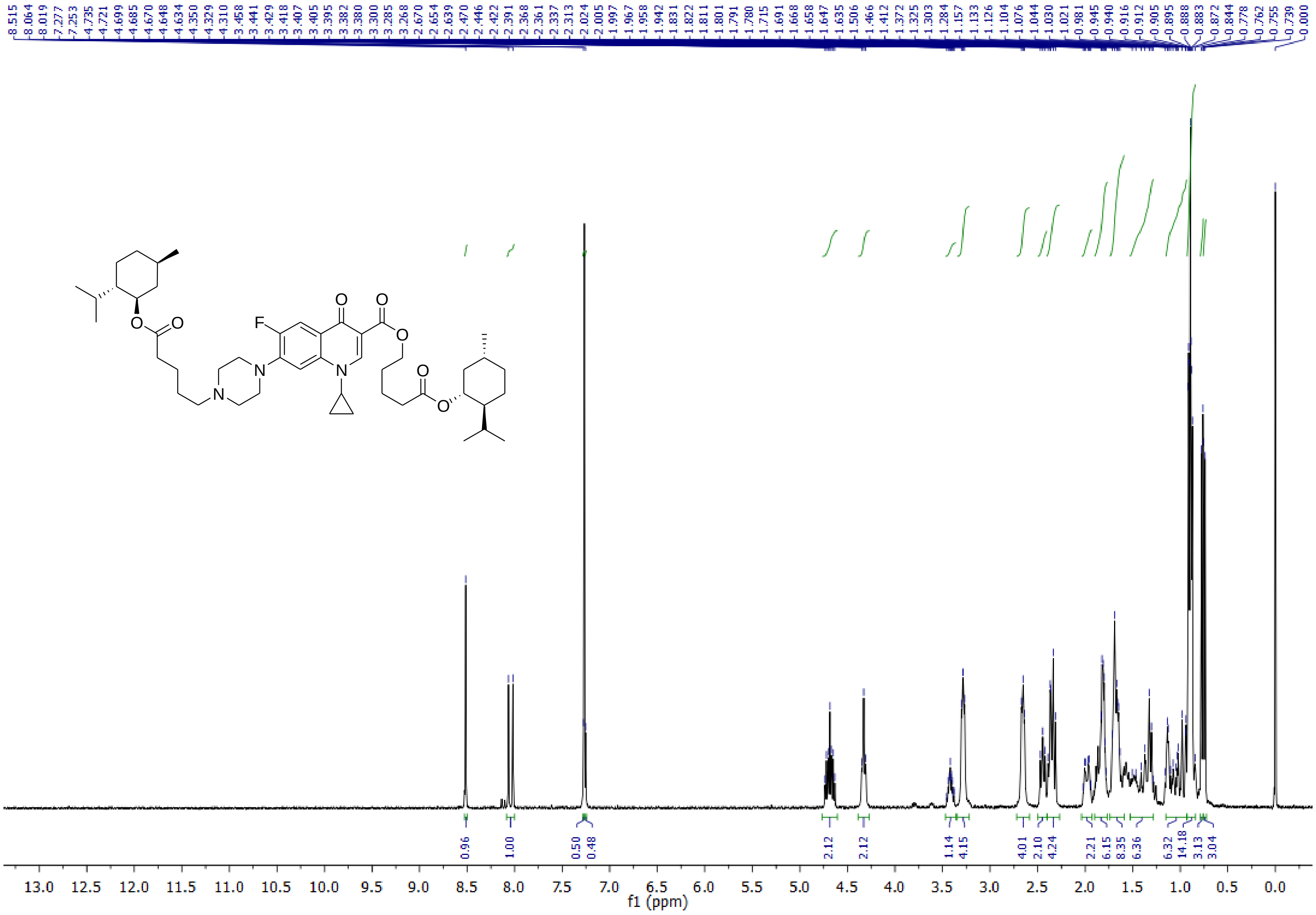

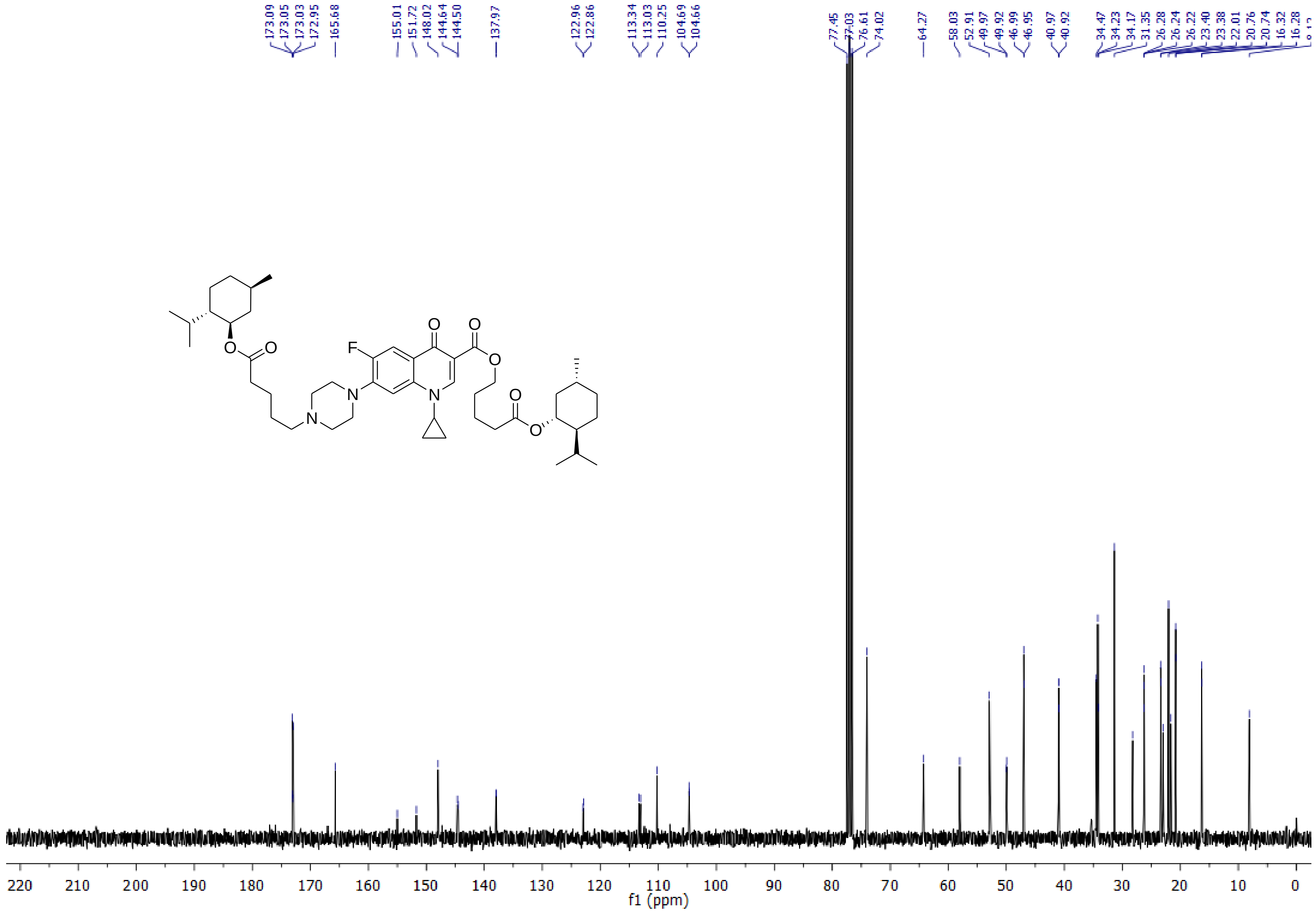
173.09
173.05
173.03
172.95
165.68
155.01
151.72
148.02
144.64
144.50
137.97
122.96
122.86
113.34
113.03
110.25
104.69
104.66
77.45
77.03
76.61
74.02
64.27
58.03
52.91
49.97
49.92
46.99
46.95
40.97
40.92
34.47
34.23
34.17
31.35
26.28
26.24
26.22
23.40
23.38
22.01
20.76
20.74
16.32
16.28
F
O
N
220 210 200 190 180 170 160 150 140 130 120 110 100 90 80 70 60 50 40 30 20 10 0
f1 (ppm)

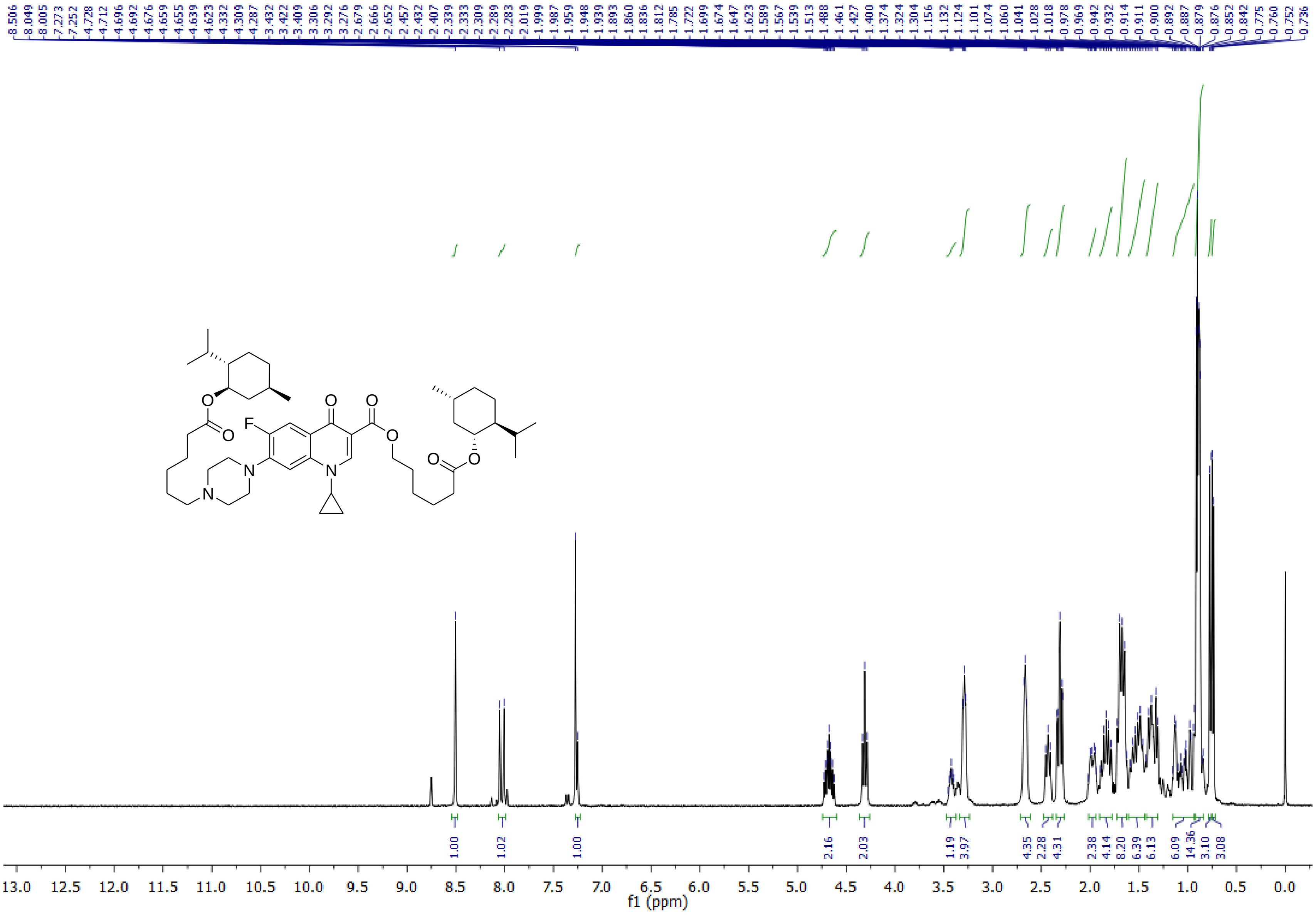
8.506 8.049 8.005 7.273 7.252 4.728 4.712 4.696 4.692 4.676 4.659 4.655 4.639 4.623 4.332 4.309 4.287 3.432 3.422 3.409 3.306 3.292 3.276 2.679 2.666 2.652 2.457 2.432 2.407 2.339 2.333 2.309 2.289 2.283 2.019 1.999 1.987 1.959 1.948 1.939 1.893 1.860 1.836 1.812 1.785 1.722 1.699 1.674 1.647 1.623 1.589 1.567 1.539 1.513 1.488 1.461 1.427 1.400 1.374 1.324 1.304 1.156 1.132 1.124 1.101 1.074 1.060 1.041 1.028 1.018 0.978 0.969 0.942 0.932 0.914 0.911 0.900 0.892 0.887 0.879 0.876 0.852 0.842 0.775 0.760 0.752 0.736
1.00
1.02
1.00
2.16
2.03
1.19
3.97
4.35
2.28
4.31
2.38
4.14
8.20
6.39
6.13
6.09
14.36
3.10
3.08
13.0 12.5 12.0 11.5 11.0 10.5 10.0 9.5 9.0 8.5 8.0 7.5 7.0 6.5 6.0 5.5 5.0 4.5 4.0 3.5 3.0 2.5 2.0 1.5 1.0 0.5 0.0
f1 (ppm)

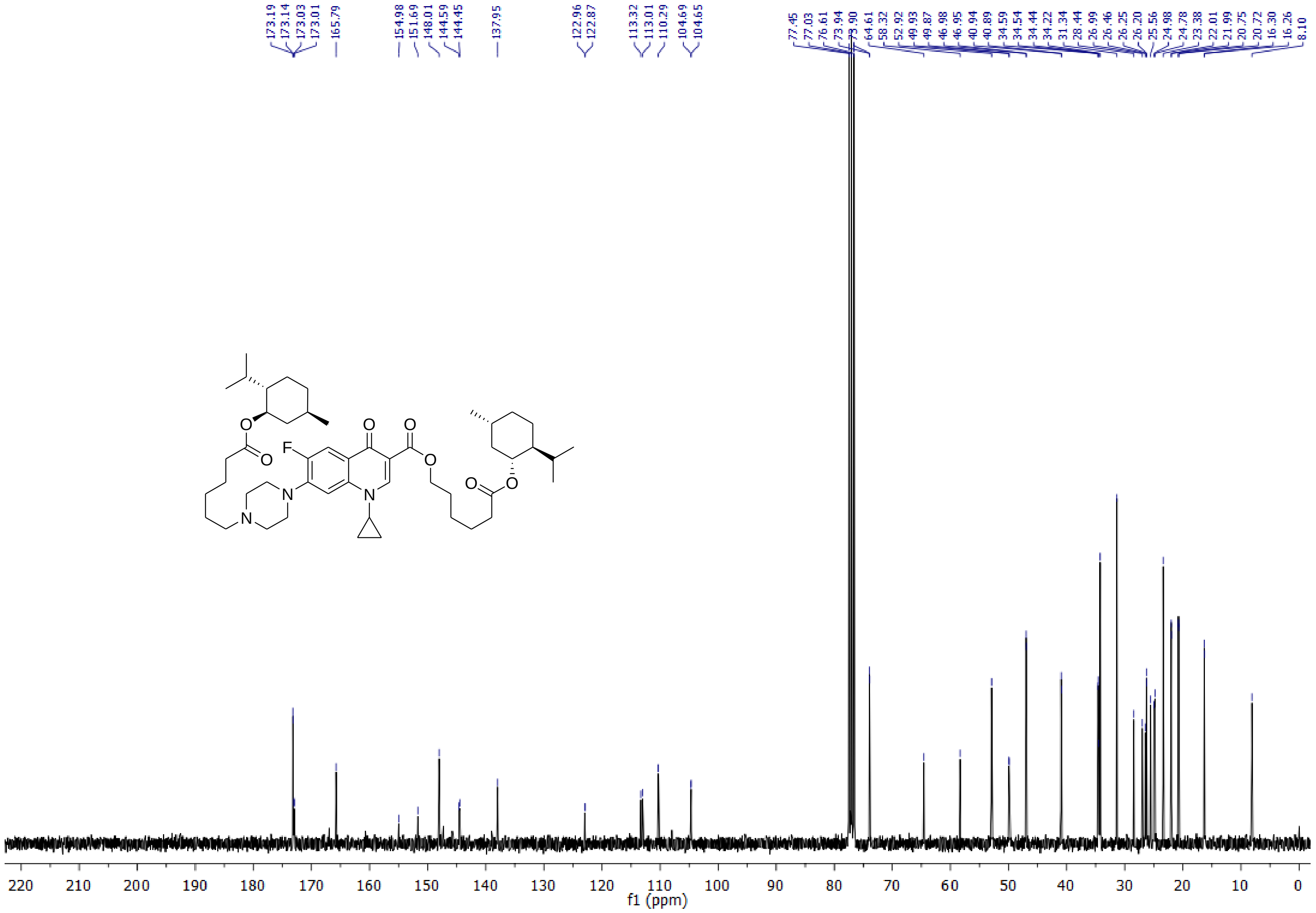
173.19
173.14
173.03
173.01
165.79
154.98
151.69
148.01
144.59
144.45
137.95
122.96
122.87
113.32
113.01
110.29
104.69
104.65
77.45
77.03
76.61
73.94
73.90
64.61
58.32
52.92
49.93
49.87
46.98
46.95
40.94
40.89
34.59
34.54
34.44
34.22
31.34
28.44
26.99
26.46
26.25
26.20
25.56
24.98
24.78
23.38
22.01
21.99
20.75
20.72
16.30
16.26
8.10
220 210 200 190 180 170 160 150 140 130 120 110 100 90 80 70 60 50 40 30 20 10 0
f1 (ppm)

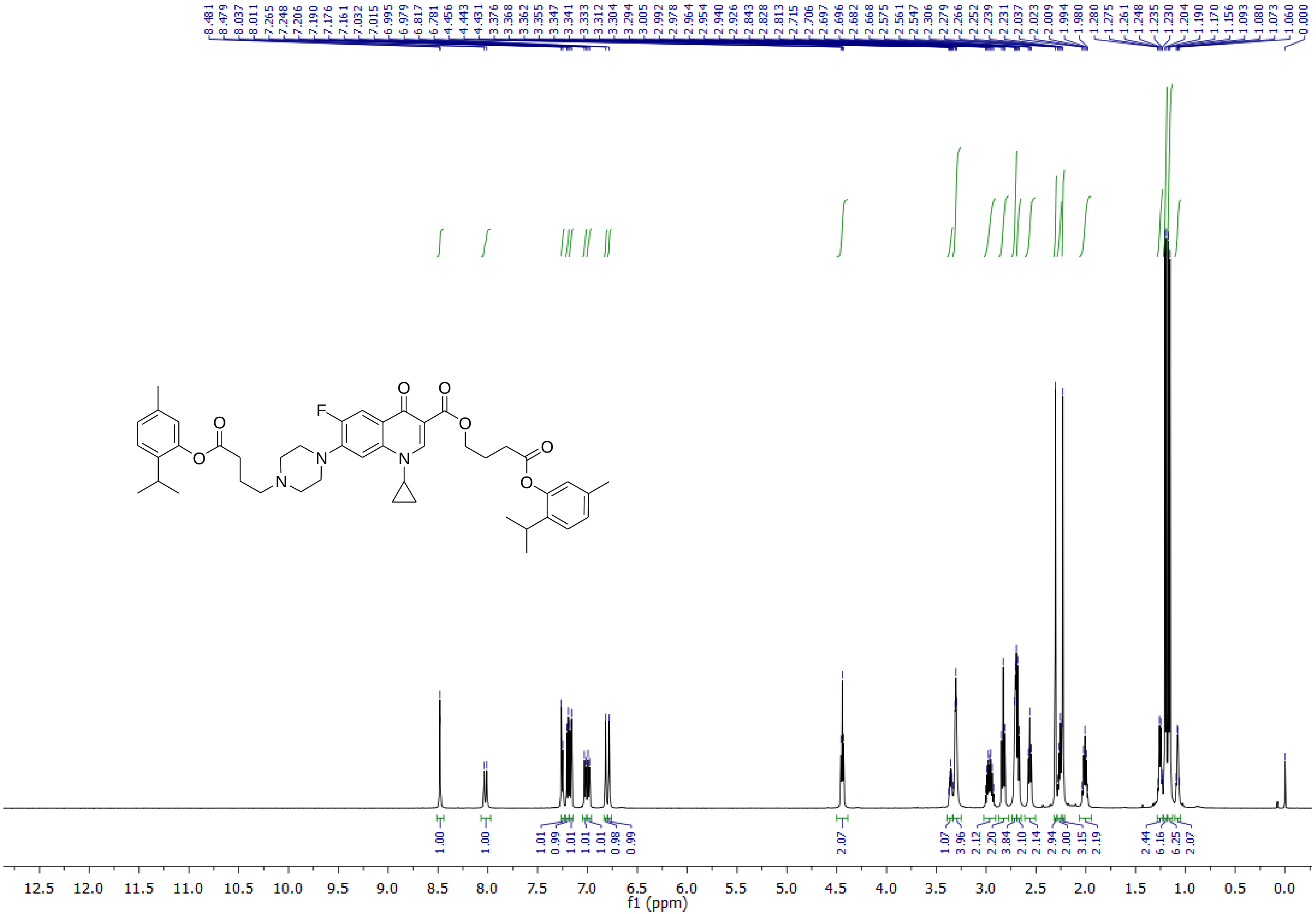
8.481 8.479 8.037 8.011 7.265 7.248 7.206 7.190 7.176 7.161 7.032 7.015 6.995 6.979 6.817 6.781 4.456 4.443 4.431 3.376 3.368 3.362 3.355 3.347 3.341 3.333 3.312 3.304 3.294 3.005 2.992 2.978 2.964 2.954 2.940 2.926 2.843 2.828 2.813 2.715 2.706 2.697 2.696 2.682 2.668 2.575 2.561 2.547 2.306 2.279 2.266 2.252 2.239 2.231 2.037 2.023 2.009 1.994 1.980 1.280 1.275 1.261 1.248 1.235 1.230 1.204 1.190 1.170 1.156 1.093 1.080 1.073 1.060 0.000
F
O
N
1.00 1.00 1.01 0.99 1.01 1.01 1.01 0.98 0.99 2.07 1.07 3.96 2.12 2.20 3.84 2.10 2.14 2.94 2.00 3.15 2.19 2.44 6.16 6.25 2.07
12.5 12.0 11.5 11.0 10.5 10.0 9.5 9.0 8.5 8.0 7.5 7.0 6.5 6.0 5.5 5.0 4.5 4.0 3.5 3.0 2.5 2.0 1.5 1.0 0.5 0.0
f1 (ppm)

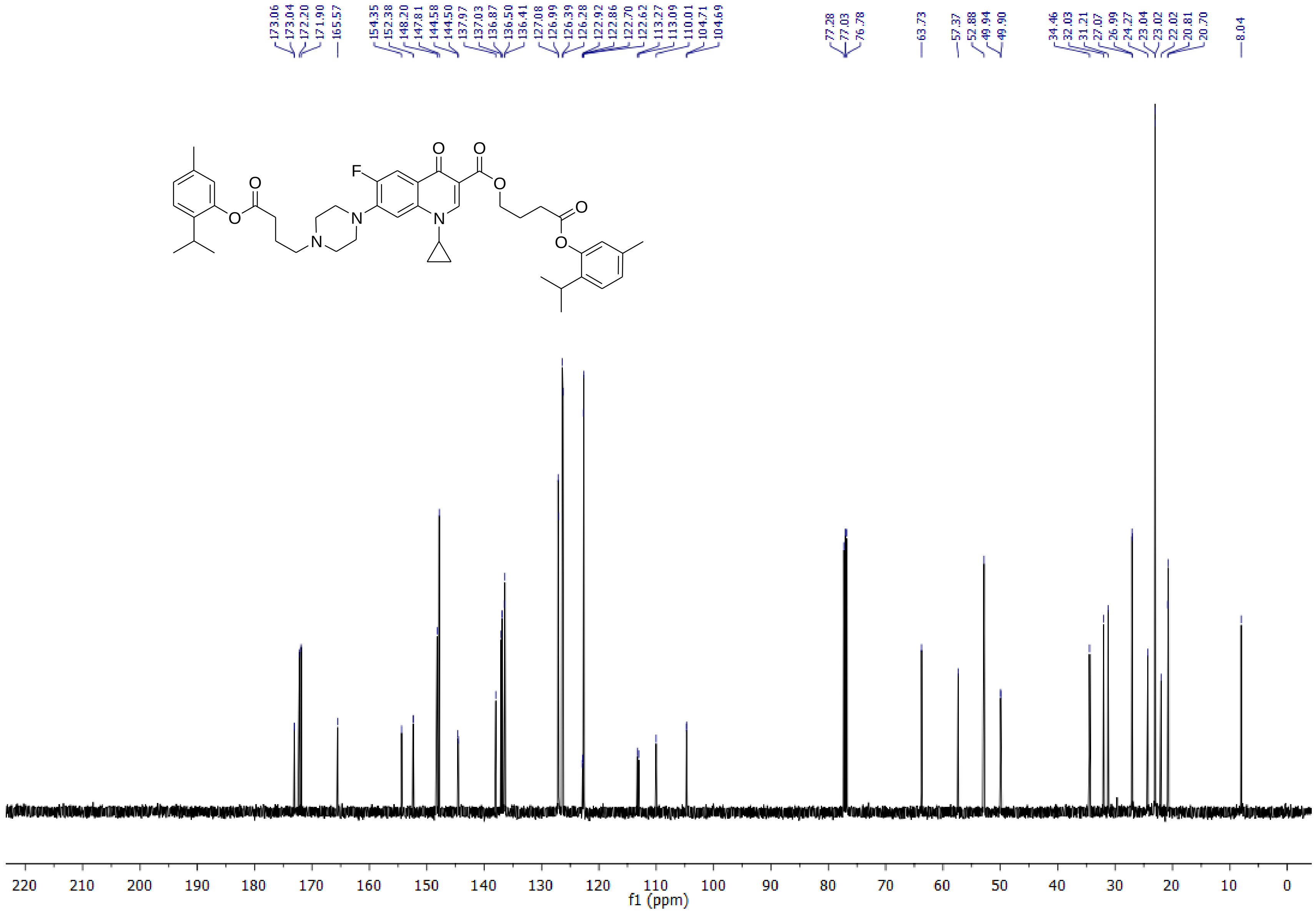
173.06
173.04
172.20
171.90
165.57
154.35
152.38
148.20
147.81
144.58
144.50
137.97
137.03
136.87
136.50
136.41
127.08
126.99
126.39
126.28
122.92
122.86
122.70
122.62
113.27
113.09
110.01
104.71
104.69
77.28
77.03
76.78
63.73
57.37
52.88
49.94
49.90
34.46
32.03
31.21
27.07
26.99
24.27
23.04
23.02
22.02
20.81
20.70
8.04
F
O
N
220 210 200 190 180 170 160 150 140 130 120 110 100 90 80 70 60 50 40 30 20 10 0
f1 (ppm)

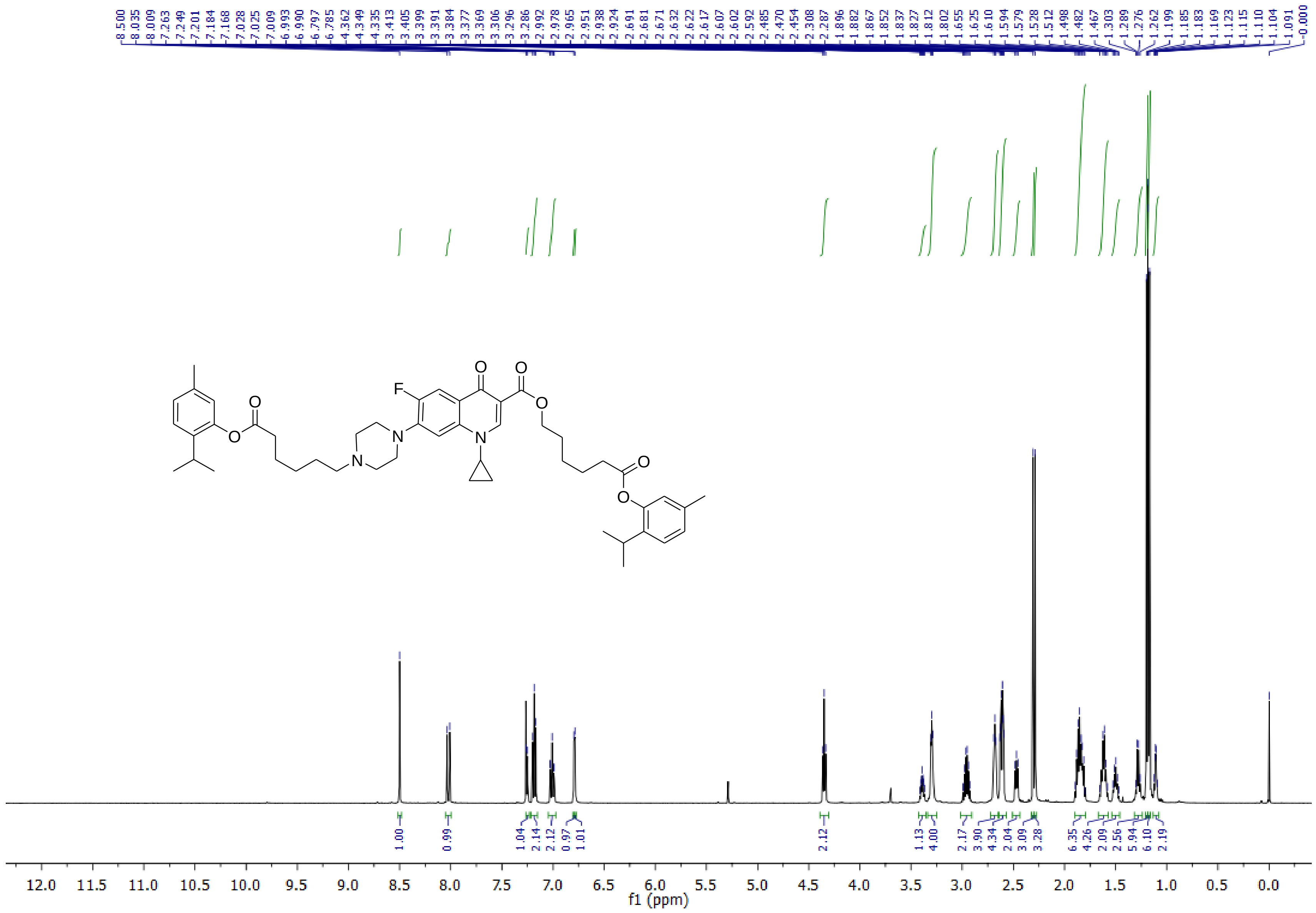
8.500 8.035 8.009 7.263 7.249 7.201 7.184 7.168 7.028 7.025 7.009 6.993 6.990 6.797 6.785 4.362 4.349 4.335 3.413 3.405 3.399 3.391 3.384 3.377 3.369 3.306 3.296 3.286 2.992 2.978 2.965 2.951 2.938 2.924 2.691 2.681 2.671 2.632 2.622 2.617 2.607 2.602 2.592 2.485 2.470 2.454 2.308 2.287 1.896 1.882 1.867 1.852 1.837 1.827 1.812 1.802 1.655 1.625 1.610 1.594 1.579 1.528 1.512 1.498 1.482 1.467 1.303 1.289 1.276 1.262 1.199 1.185 1.183 1.169 1.123 1.115 1.110 1.104 1.091 -0.000
1.00 0.99 1.04 2.14 2.12 0.97 1.01 2.12 1.13 4.00 2.17 3.90 4.34 2.04 3.09 3.28 6.35 4.26 2.09 2.56 5.94 6.10 2.19
12.0 11.5 11.0 10.5 10.0 9.5 9.0 8.5 8.0 7.5 7.0 6.5 6.0 5.5 5.0 4.5 4.0 3.5 3.0 2.5 2.0 1.5 1.0 0.5 0.0
f1 (ppm)

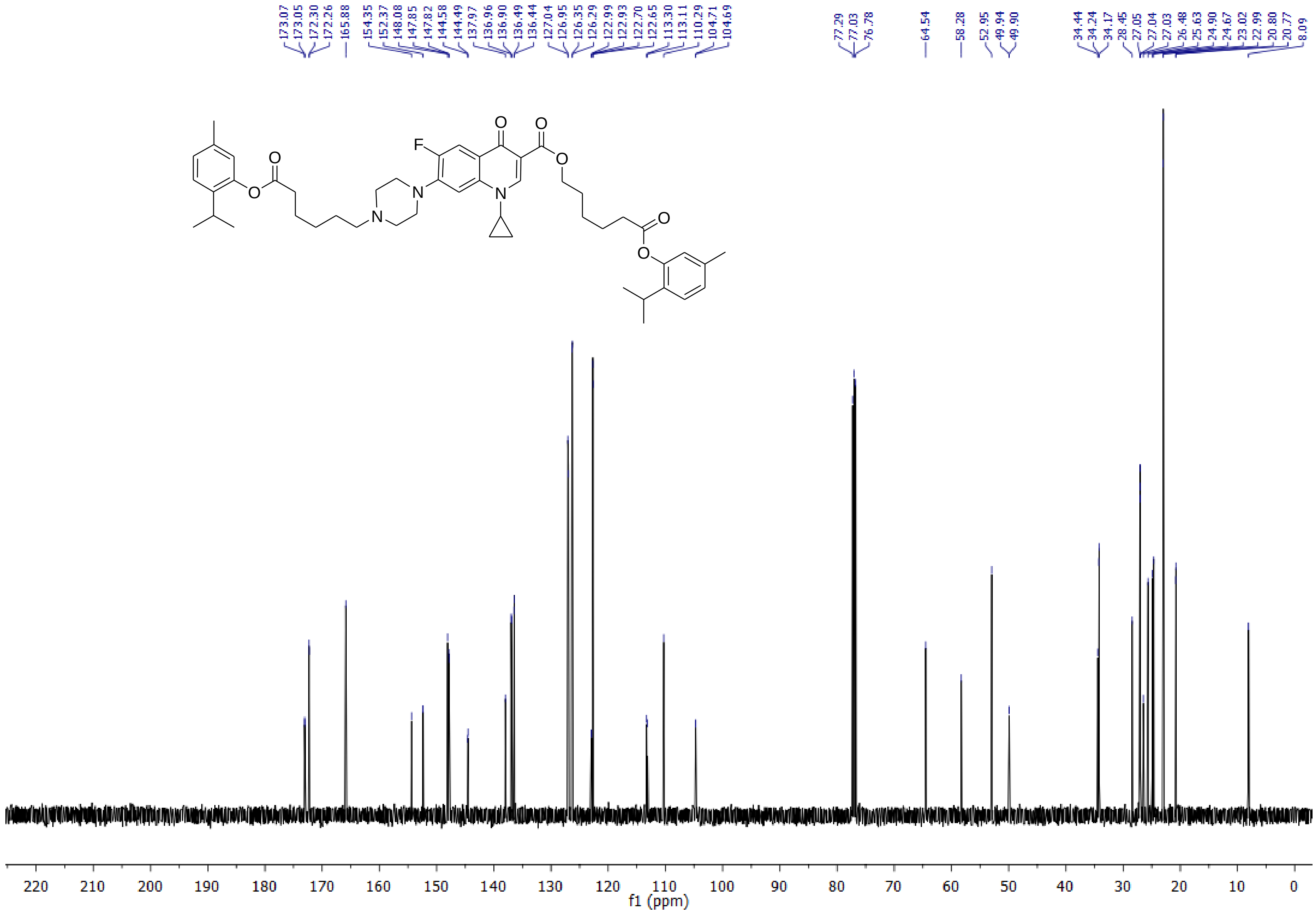
173.07
173.05
172.30
172.26
165.88
154.35
152.37
148.08
147.85
147.82
144.58
144.49
137.97
136.96
136.90
136.49
136.44
127.04
126.95
126.35
126.29
122.99
122.93
122.70
122.65
113.30
113.11
110.29
104.71
104.69
77.29
77.03
76.78
64.54
58.28
52.95
49.94
49.90
34.44
34.24
34.17
28.45
27.05
27.04
27.03
26.48
25.63
24.90
24.67
23.02
22.99
20.80
20.77
8.09
220 210 200 190 180 170 160 150 140 130 120 110 100 90 80 70 60 50 40 30 20 10 0
f1 (ppm)

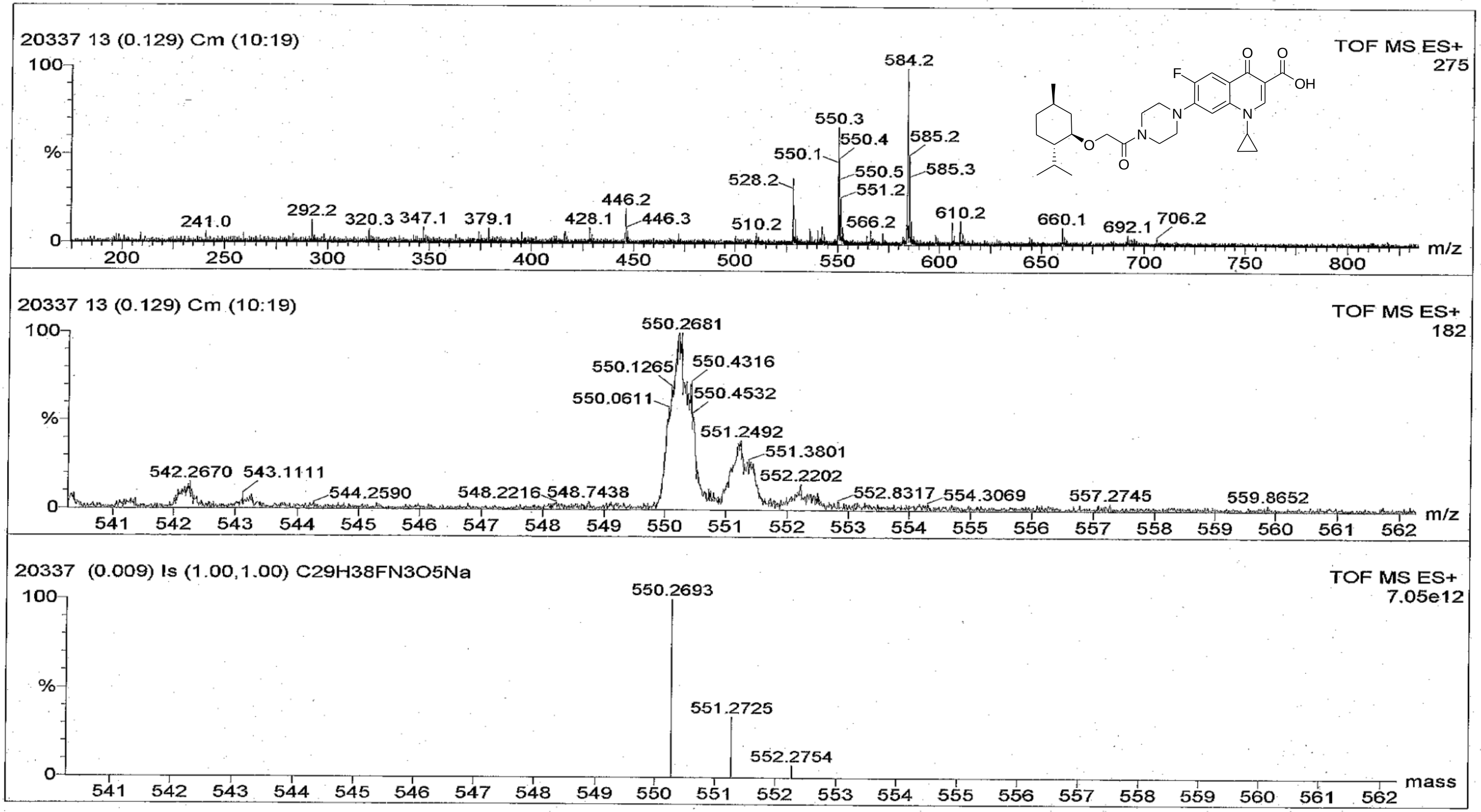

20337 13 (0.129) Cm (10:19)
TOF MS ES+
275
100
%
0
584.2
550.3
550.4
550.1
585.2
550.5
585.3
528.2
551.2
446.2
292.2
241.0
320.3
347.1
379.1
428.1
446.3
510.2
566.2
610.2
660.1
692.1
706.2
m/z
200
250
300
350
400
450
500
550
600
650
700
750
800
F
O
O
OH
N
N
N
O
O
20337 13 (0.129) Cm (10:19)
TOF MS ES+
182
550.2681
550.1265
550.4316
550.0611
550.4532
551.2492
551.3801
552.2202
542.2670
543.1111
544.2590
548.2216
548.7438
552.8317
554.3069
557.2745
559.8652
m/z
20337 (0.009) Is (1.00,1.00) C29H38FN3O5Na
TOF MS ES+
7.05e12
550.2693
551.2725
552.2754
mass
541
542
543
544
545
546
547
548
549
550
551
552
553
554
555
556
557
558
559
560
561
562

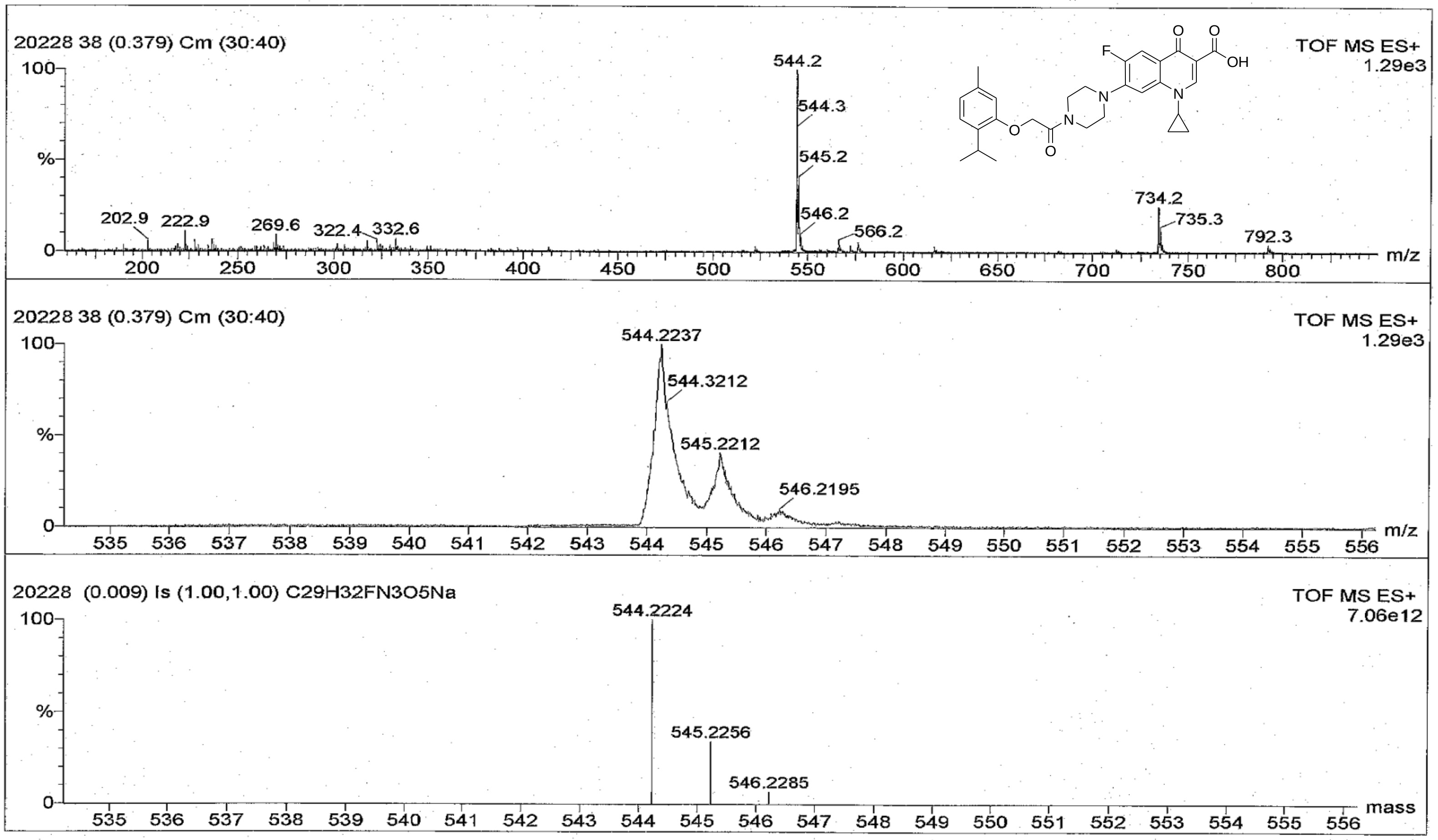
20228 38 (0.379) Cm (30:40)
TOF MS ES+
1.29e3
544.2
544.3
545.2
546.2
566.2
202.9
222.9
269.6
322.4
332.6
734.2
735.3
792.3
m/z
F
O
O
OH
N
N
N
O
O
20228 38 (0.379) Cm (30:40)
TOF MS ES+
1.29e3
544.2237
544.3212
545.2212
546.2195
m/z
20228 (0.009) Is (1.00,1.00) C29H32FN3O5Na
TOF MS ES+
7.06e12
544.2224
545.2256
546.2285
mass

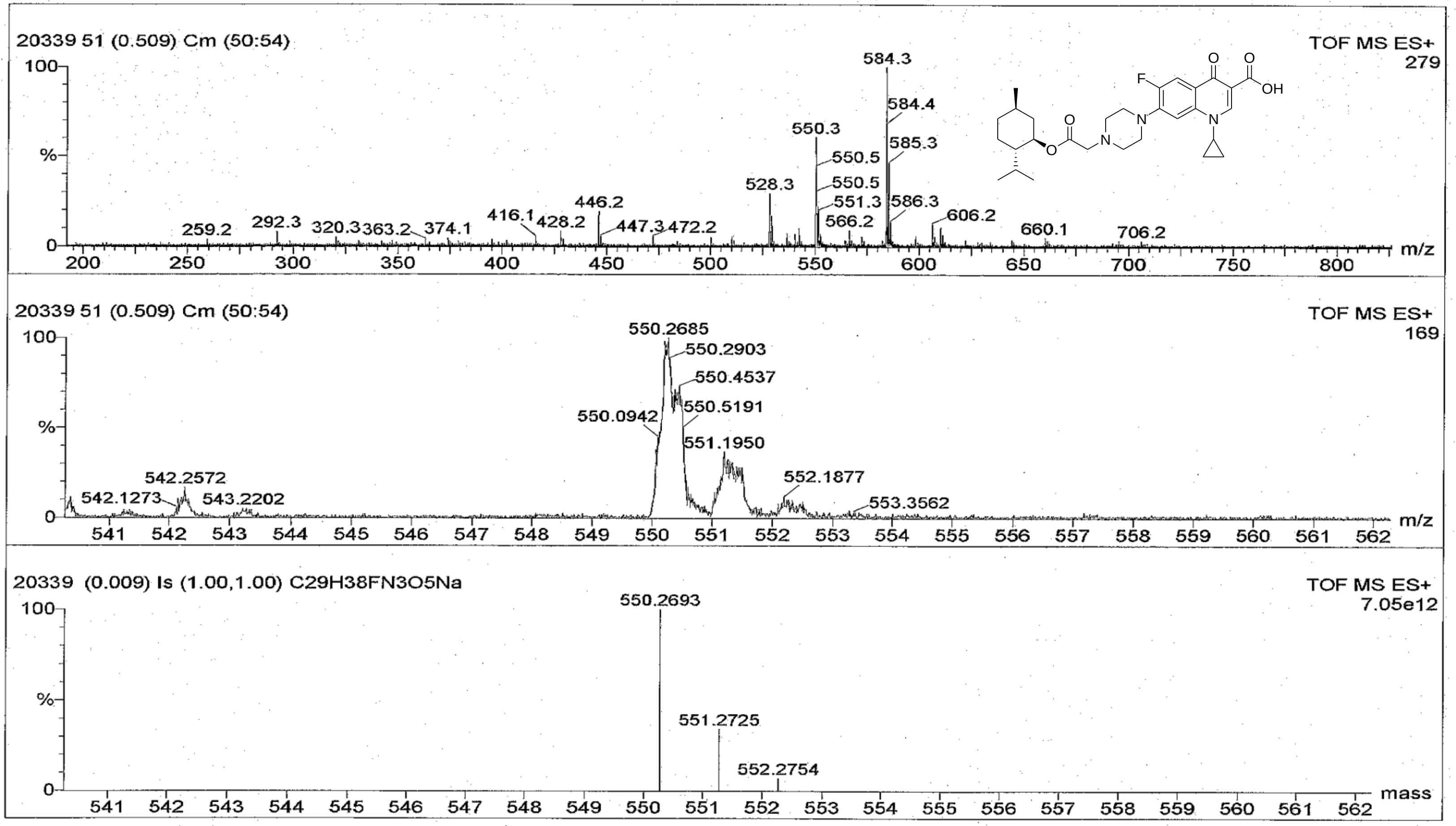

20339 51 (0.509) Cm (50:54)
TOF MS ES+
279
584.3
584.4
585.3
550.3
550.5
550.5
551.3
528.3
586.3
566.2
606.2
446.2
416.1
428.2
447.3
472.2
259.2
292.3
320.3
363.2
374.1
660.1
706.2
F
O
O
OH
N
N
N
O
O
m/z
20339 51 (0.509) Cm (50:54)
TOF MS ES+
169
550.2685
550.2903
550.4537
550.0942
550.5191
551.1950
542.2572
542.1273
543.2202
552.1877
553.3562
20339 (0.009) Is (1.00,1.00) C29H38FN3O5Na
TOF MS ES+
7.05e12
550.2693
551.2725
552.2754
mass

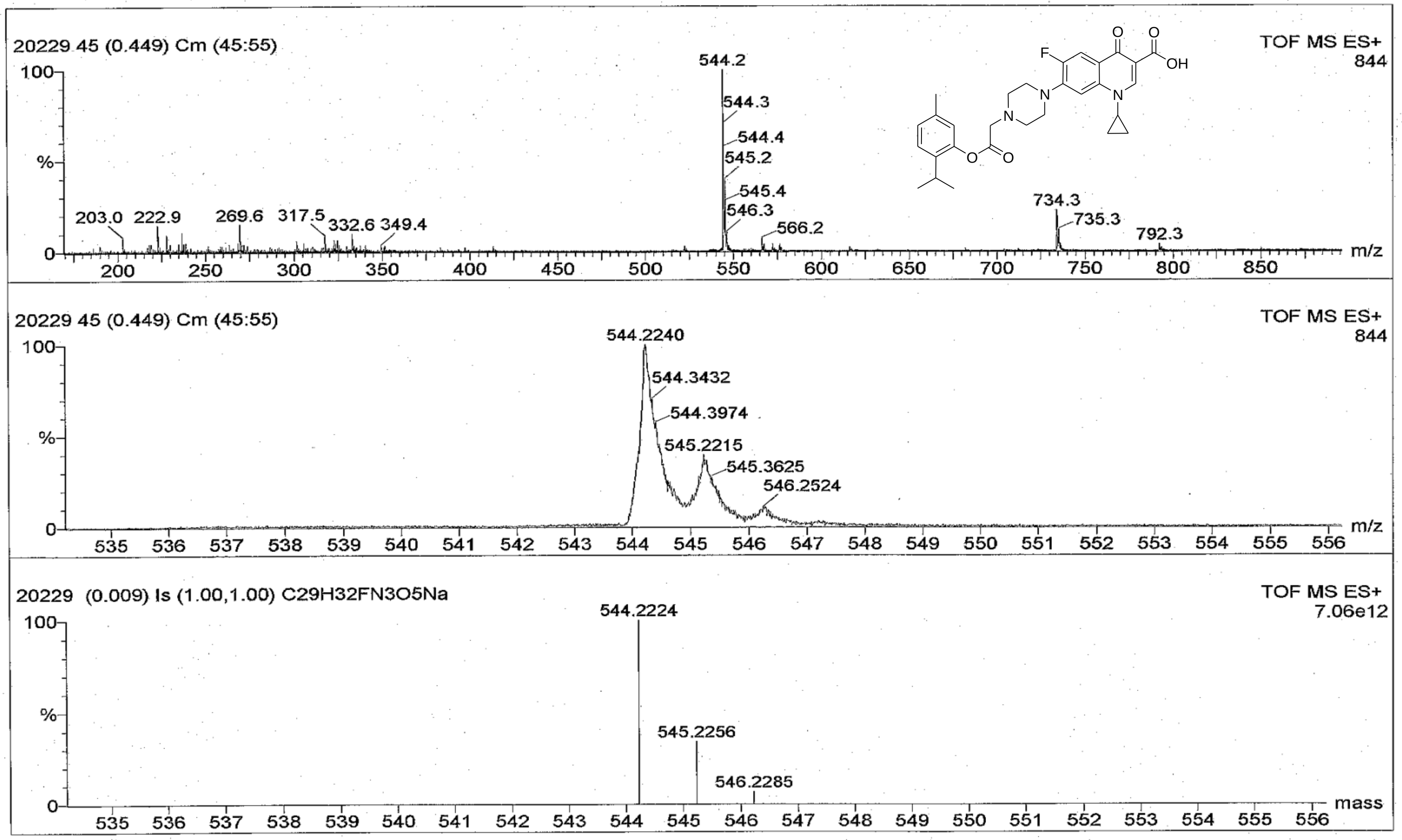
20229 45 (0.449) Cm (45:55)
TOF MS ES+
844
544.2
544.3
544.4
545.2
545.4
546.3
566.2
203.0
222.9
269.6
317.5
332.6
349.4
734.3
735.3
792.3
m/z
F
O
O
OH
N
N
N
O
O
20229 45 (0.449) Cm (45:55)
TOF MS ES+
844
544.2240
544.3432
544.3974
545.2215
545.3625
546.2524
m/z
20229 (0.009) Is (1.00,1.00) C29H32FN3O5Na
TOF MS ES+
7.06e12
544.2224
545.2256
546.2285
mass

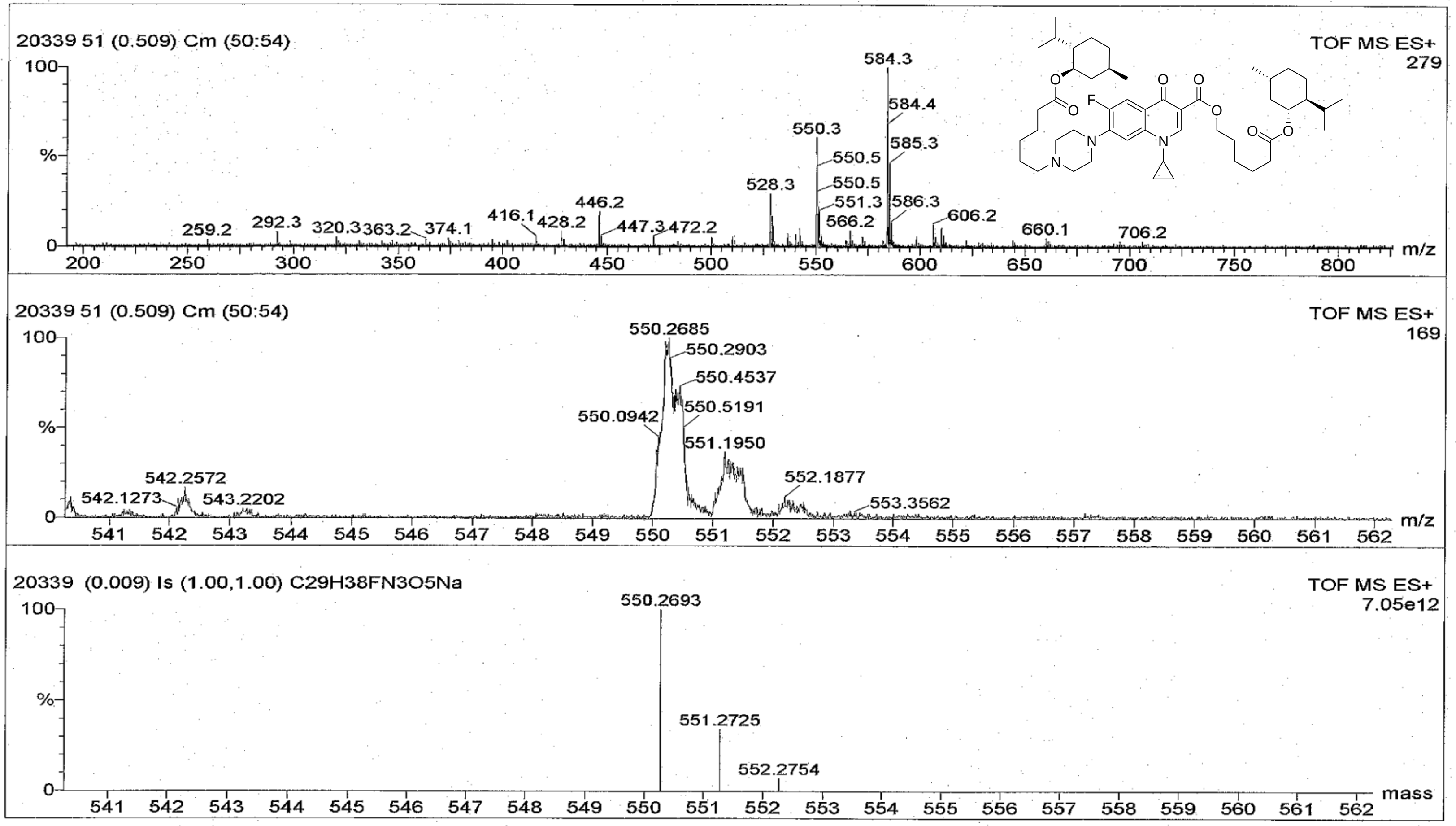
20339 51 (0.509) Cm (50:54)
TOF MS ES+
279
584.3
584.4
585.3
586.3
550.3
550.5
550.5
551.3
566.2
528.3
606.2
660.1
706.2
446.2
447.3
472.2
416.1
428.2
259.2
292.3
320.3
363.2
374.1
m/z
20339 51 (0.509) Cm (50:54)
TOF MS ES+
169
550.2685
550.2903
550.4537
550.5191
550.0942
551.1950
552.1877
553.3562
542.2572
542.1273
543.2202
m/z
20339 (0.009) Is (1.00,1.00) C29H38FN3O5Na
TOF MS ES+
7.05e12
550.2693
551.2725
552.2754
mass

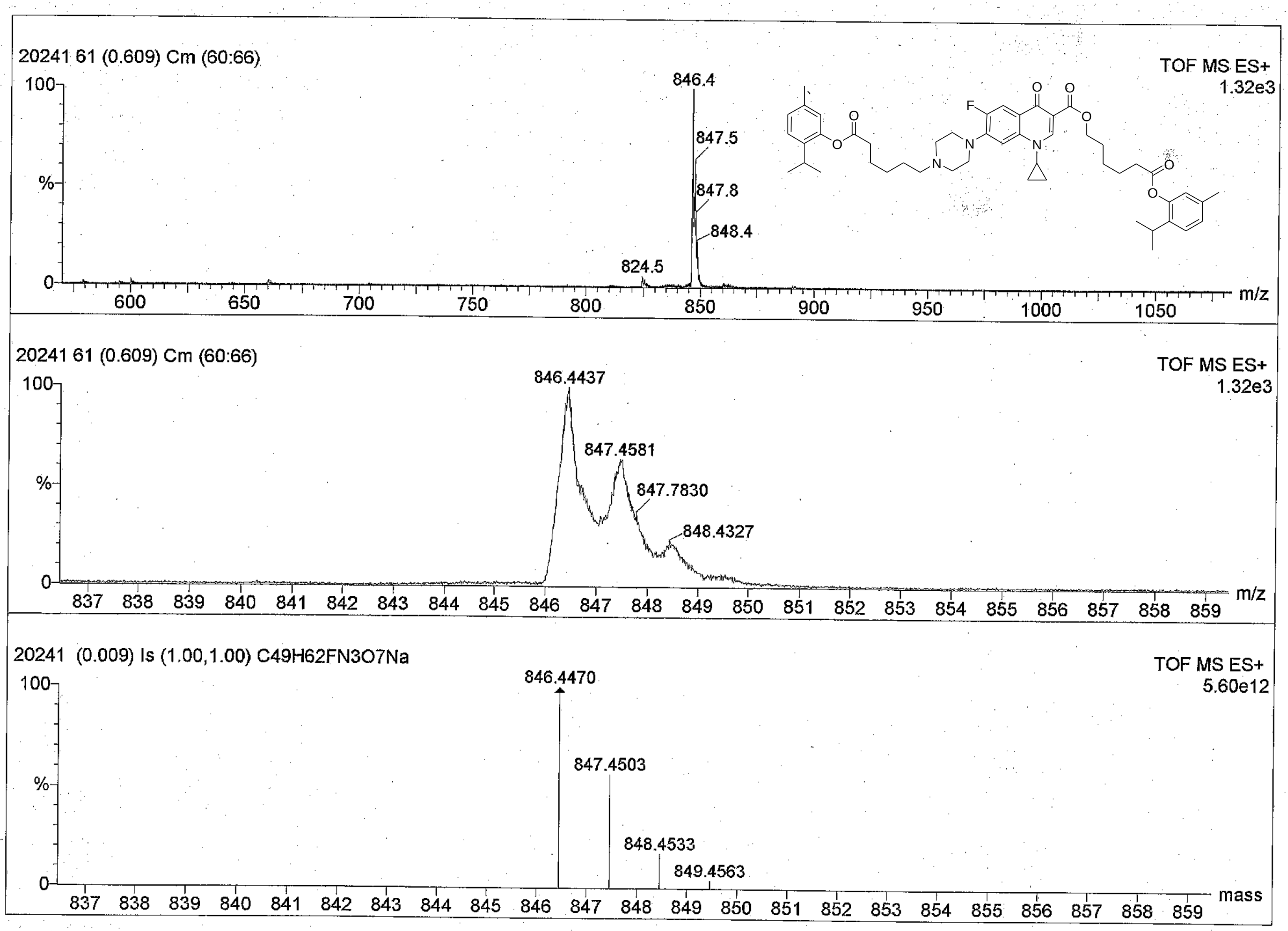
20241 61 (0.609) Cm (60:66)
TOF MS ES+
1.32e3
846.4
847.5
847.8
848.4
824.5
m/z
F
O
N
20241 61 (0.609) Cm (60:66)
TOF MS ES+
1.32e3
846.4437
847.4581
847.7830
848.4327
m/z
20241 (0.009) Is (1.00,1.00) C49H62FN3O7Na
TOF MS ES+
5.60e12
846.4470
847.4503
848.4533
849.4563
mass
